# Comparative Effectiveness of Nutritional Strategies for Sarcopenia-Related Outcomes in Older Adults: A Systematic Review and Network Meta-Analysis

**DOI:** 10.3390/nu18203368

**Published:** 2026-10-14

**Authors:** Xinxin Zhang, Simin Fan, Junru Zan, Min Chen, Xueyi Chen

**Affiliations:** 1 School of Physical Education, Shaanxi Normal University, Xi’an 710119, China; zhangxx9606@snnu.edu.cn (X.Z.); 15647213017@163.com (J.Z.); 2 School of Physical Education and Art, East China University of Technology, Nanchang 330013, China; 2025216067@ecut.edu.cn

**Keywords:** sarcopenia, nutritional supplementation, network meta-analysis, muscle mass, muscle strength, physical performance

## Abstract

**Objective:** This study compared the relative effects of distinct nutritional strategies on muscle mass, muscle strength, and physical performance in older adults with sarcopenia or at high risk of sarcopenia. **Methods:** PubMed, Embase, the Cochrane Library, Web of Science, and CNKI were systematically searched for randomized controlled trials (RCTs). Random-effects pairwise meta-analysis and network meta-analysis (NMA) were performed. The surface under the cumulative ranking curve (SUCRA) was used to summarize relative rankings, and CINeMA was applied to assess the certainty of evidence. **Results:** Twenty RCTs involving 2160 participants were included, forming 17 network nodes. Nutritional interventions collectively improved appendicular skeletal muscle mass index (ASMI), grip strength, and gait speed, whereas the pooled effect on the five-times sit-to-stand test (5STS) was not statistically significant. In the NMA, whey protein + vitamin D + leucine improved ASMI (SMD = 0.29, 95% CI: 0.06 to 0.52) and grip strength (SMD = 0.82, 95% CI: 0.13 to 1.50), and it reduced 5STS completion time (SMD = −1.41, 95% CI: −2.63 to −0.18). Omega-3 fatty acids + leucine + Lactobacillus paracasei PS23 improved ASMI (SMD = 0.67, 95% CI: 0.10 to 1.25) and grip strength (SMD = 1.84, 95% CI: 0.39 to 3.28), although this node was supported by a single RCT. Leucine + vitamin D + medium-chain triglycerides improved gait speed (SMD = 1.28, 95% CI: 0.13 to 2.43), although this node was also supported by a single RCT. Sensitivity analyses indicated that the magnitude and precision of some formulation-specific estimates varied with the exclusion of individual trials or restriction to participants with confirmed sarcopenia. The certainty of evidence for the principal comparisons ranged from low to very low. **Conclusions:** Nutritional supplementation showed overall benefits for ASMI, grip strength, and gait speed, while formulation-specific effects varied across outcomes. Whey protein + vitamin D + leucine showed favorable effects across several outcomes, whereas omega-3 fatty acids + leucine + Lactobacillus paracasei PS23 and leucine + vitamin D + medium-chain triglycerides showed additional formulation-specific signals based on single-study nodes. Given the sparse network and very low certainty of evidence for the principal comparisons, these findings should be regarded as candidate signals rather than evidence of a definitive treatment hierarchy.

## 1. Introduction

Global population aging continues to accelerate, and age-related declines in skeletal muscle mass and function have become major threats to independent living and healthy longevity in older adults [1,2]. Sarcopenia is characterized by progressive, generalized loss of skeletal muscle mass and function. It is closely associated with falls, mobility limitations, hospitalization, reduced quality of life, and increased mortality risk [3,4]. Muscle mass, muscle strength, and physical performance are key dimensions in diagnosing and grading sarcopenia severity, and each is differentially associated with the ability to perform activities of daily living and adverse health outcomes [5]. Accordingly, identifying feasible and sustainable interventions that can simultaneously improve these outcomes remains an important clinical issue in geriatric nutrition and sarcopenia management.

Nutritional intervention constitutes an important component of non-pharmacological sarcopenia management. Protein and essential amino acids provide substrates for muscle protein synthesis; leucine participates in the regulation of anabolic signaling; vitamin D is related to muscle metabolism and the maintenance of muscle function; and omega-3 fatty acids, β-hydroxy-β-methylbutyrate (HMB), and probiotics have also been investigated for their potential to modulate inflammation, anabolic resistance, or the gut–muscle axis [6,7,8]. However, nutrients differ considerably in their biological targets, doses, formulations, and combinations with exercise, leading to inconsistent findings across clinical studies. Previous meta-analyses have mainly evaluated individual components or relatively broad nutritional categories, such as vitamin D combined with protein, omega-3 fatty acids, leucine, whey protein, or protein supplementation [9,10,11,12]. Although these analyses can determine whether a given nutritional category is effective, they are less able to directly compare the relative effects of specific formulations.

Network meta-analysis can integrate direct and indirect evidence within a unified framework, provided that the assumptions of transitivity and consistency are met [13]. In recent years, studies have compared exercise and protein-based interventions or included both nutritional and pharmacological treatments in network comparisons [14,15]. In the nutritional management of sarcopenia, however, grouping distinct formulations into broad nutritional categories may obscure differences in their core nutrient composition. Moreover, muscle mass, grip strength, gait speed, and sit-to-stand performance represent different physiological dimensions, and the effects of nutritional strategies may therefore not be consistent across outcomes. In addition, identifying the best intervention solely based on ranking results, without adequately considering single-study nodes, network sparsity, and certainty of evidence, may lead to overinterpretation of clinical effects [16].

Accordingly, this systematic review of the twenty published controlled clinical trials included adults aged ≥60 years with diagnosed sarcopenia or at high risk of sarcopenia, defined formulations with different core nutrient compositions as separate network nodes, and systematically compared the relative effects of nutritional strategies on appendicular skeletal muscle mass index (ASMI), grip strength, gait speed, and five-times sit-to-stand test (5STS). In addition to conventional pairwise meta-analysis, this investigation performed network meta-analysis (NMA), assessed transitivity and inconsistency, generated the surface under the cumulative ranking curve (SUCRA)-based relative rankings, conducted sensitivity analyses of sparsely represented nodes, and evaluated the certainty of evidence using CINeMA. This study did not seek to establish a definitive nutritional hierarchy independent of evidence quality. Instead, it aimed to identify potentially advantageous strategies by jointly considering effect magnitude, ranking stability, and certainty of evidence, while explicitly characterizing the uncertainty of the current evidence. This approach was intended to provide formulation-level evidence for future nutritional intervention research and clinical decision-making.

## 2. Methods

This study was conducted in accordance with the methodological guidance of the Cochrane Handbook for Systematic Reviews of Interventions and was reported according to the Preferred Reporting Items for Systematic Reviews and Meta-Analyses (PRISMA 2020) and its extension for network meta-analyses (PRISMA-NMA) [17,18,19]. The review protocol was registered prospectively with the International Prospective Register of Systematic Reviews (PROSPERO; registration number: CRD420261498361).

### 2.1. Data Sources and Search Strategy

PubMed, Embase, the Cochrane Library, Web of Science, and the China National Knowledge Infrastructure (CNKI) were systematically searched from database inception to 15 June 2026, without language restrictions. Search strategies were tailored to each database and combined controlled vocabulary with free-text terms across two conceptual domains. The first domain concerned sarcopenia and related risk states, including terms such as sarcopenia, probable sarcopenia, and low muscle mass [20]. The second domain concerned nutritional interventions, including nutritional supplementation, protein, amino acids, leucine, vitamin D, omega-3 fatty acids, HMB, probiotics, and other relevant nutrients or nutrient combinations. The two conceptual domains were combined using AND, whereas related terms within each domain were combined using OR. In addition, the reference lists of included studies and relevant systematic reviews were hand-searched to identify any potentially eligible studies that may have been missed.

### 2.2. Eligibility Criteria

Studies were eligible if they met the following criteria. First, the study design had to be a randomized controlled trial (RCT). Second, participants had to be adults aged ≥60 years who met diagnostic criteria for sarcopenia or were considered to be at high risk of sarcopenia. Sarcopenia was diagnosed according to recognized criteria, such as those of the Asian Working Group for Sarcopenia (AWGS) or the European Working Group on Sarcopenia in Older People (EWGSOP) [21,22]. Studies that did not use these criteria were included only when the original investigators explicitly defined participants as having sarcopenia, probable sarcopenia, low muscle mass, or another high-risk condition closely related to sarcopenia. The population definition used in the original study was recorded [23]. The primary analysis included both participants with confirmed sarcopenia and those identified as being at high risk of sarcopenia to capture the available randomized evidence on nutritional interventions across the broader spectrum of sarcopenia-related impairment. Because differences in diagnostic definitions and baseline sarcopenia status could contribute to clinical heterogeneity and potentially modify responses to nutritional supplementation, these factors were explicitly considered in subgroup, transitivity, and sensitivity analyses [21,22,23]. Third, interventions had to involve nutritional supplementation, including single nutrients or combinations of multiple nutrients, such as protein, amino acids, vitamin D, omega-3 fatty acids, HMB, probiotics, and other relevant nutritional supplementation strategies. If a study also included exercise or another non-nutritional intervention, it was eligible only when the same co-intervention was applied in the same manner to all randomized groups. This condition helped ensure, as far as possible, that the main between-group difference was attributable to nutritional supplementation. Fourth, the comparator had to be placebo, usual diet, or no additional nutritional supplementation. Fifth, at least one of the following outcomes had to be reported: ASMI, grip strength, gait speed, or 5STS [24].

Studies were excluded if they met any of the following criteria: nonrandomized studies, animal studies, case reports, observational studies, reviews, study protocols, theses, or conference abstracts; muscle loss or functional decline primarily secondary to malignancy, severe organ failure, or another specific disease; non-nutritional co-interventions imbalanced between groups, preventing isolation of the independent effect of the nutritional intervention; or unavailable full text or outcome data that could not be obtained, extracted, or calculated.

### 2.3. Study Selection

All records identified through the searches were imported into EndNote X9 for management and deduplication [25]. Two reviewers (XC and SF) independently screened the records according to the prespecified inclusion and exclusion criteria, first by title and abstract and then by full-text assessment for potentially eligible studies. Disagreements were resolved through discussion. If consensus could not be reached, a third reviewer (XZ) participated in the discussion and made the final decision. Full-text exclusions and their primary reasons were recorded, and the study selection process was presented in a PRISMA flow diagram.

### 2.4. Data Extraction

Two reviewers (XC and MC) independently extracted data using a prespecified data extraction form. Extracted information included first author, year of publication, and country or region; participant characteristics, including sample size, sex, and age; sarco-penia diagnostic criteria and baseline risk status; type and detailed composition of the nutritional intervention, dose, intervention frequency, intervention duration, background co-interventions, comparator, and adherence; and outcome measures. Disagreements during data extraction were resolved through discussion. If consensus could not be reached, a third reviewer (XZ) participated in the discussion and made the final decision.

For multi-arm trials containing more than one eligible intervention group, each eligible intervention arm was extracted separately. In pairwise meta-analysis, when two or more eligible intervention groups shared the same control group, the sample size of the shared control group was divided by the number of eligible comparisons, while its mean and standard deviation were left unchanged. This approach was used to avoid double-counting shared control participants and the resulting unit-of-analysis error. In NMA, each eligible intervention arm was assigned to the corresponding prespecified intervention node according to its specific nutritional composition, and the original multi-arm trial structure was retained [17]. In Cramer et al., the sarcopenia and at-risk sarcopenia subgroups represented mutually exclusive participant populations and were therefore extracted separately; no participant was counted more than once within the same outcome analysis. When a study reported outcomes at multiple assessment time points, data at the prespecified end of the intervention were preferentially extracted to improve comparability of outcome timing across studies. Detailed nutritional composition, participant age and sex, sarcopenia diagnostic criteria, baseline population characteristics, intervention dose and frequency, co-interventions, intervention duration, and adherence are presented in Supplementary Table S1.

### 2.5. Risk-of-Bias Assessment

The Cochrane Risk of Bias 2 tool (RoB 2) was used to assess the risk of bias in the included randomized controlled trials. Five domains were evaluated: bias arising from the randomization process; bias due to deviations from intended interventions; bias due to missing outcome data; bias in measurement of the outcome; and bias in selection of the reported result. Each domain was judged as low risk, some concerns, or high risk [26].

Overall risk of bias was determined by integrating the judgments across domains. A study was judged to have an overall low risk of bias if all domains were rated as low risk. A study was judged to have some concerns if at least one domain raised some concerns, but none was rated as high risk. A study was judged to have a high risk of bias if at least one domain was rated as high risk or if concerns across multiple domains were sufficient to reduce confidence in the study results substantially. Two reviewers (XC and JZ) independently completed the risk-of-bias assessment. Disagreements were first resolved through discussion. If consensus could not be reached, a third reviewer (XZ) participated in the discussion and made the final decision.

### 2.6. Data Processing and Statistical Analysis

All outcomes included in this study were continuous variables. Because measure-ment devices, assessment protocols, and reporting scales varied across studies for some outcomes, intervention effects were expressed as standardized mean differences (SMDs) with 95% confidence intervals (CIs) to improve comparability of effect sizes across stud-ies [27]. For ASMI, grip strength, and gait speed, an SMD > 0 indicated improvement in the intervention group. For 5STS, because a shorter completion time indicates better physical performance, an SMD < 0 indicated benefit in the intervention group. Subsequent NMA and ranking analyses were interpreted consistently according to the direction of clinical improvement for each outcome.

Changes from baseline to the end of the intervention and their standard deviations were preferentially extracted. When change scores were not directly reported, mean changes were calculated from baseline and post-intervention values. When the standard deviation of the change score was missing, it was estimated from the baseline standard deviation, post-intervention standard deviation, and the correlation coefficient between baseline and post-intervention measurements. The correlation coefficient was calculated from available data in the original study whenever possible. When it could not be derived directly, a prespecified correlation coefficient (r = 0.5) was used and applied consistently across all applicable studies [17].

#### 2.6.1. Pairwise Meta-Analysis

Pairwise meta-analyses were performed using Review Manager 5.4. These analyses were primarily used to estimate the overall effects of nutritional supplementation com-pared with control conditions and served as complementary analyses to the NMA. Given the potential clinical and methodological heterogeneity across studies in participant characteristics, nutritional composition, dose, intervention duration, and study design, effect sizes were pooled using random-effects models. Between-study heterogeneity was assessed using the *I*^2^ statistic and interpreted together with the direction of effects, 95% CIs, and clinical and methodological differences among studies. *I*^2^ values of approximately 25%, 50%, and 75% were used as reference thresholds for low, moderate, and high heterogeneity, respectively [27].

For outcomes with substantial heterogeneity, subgroup analyses were conducted according to prespecified potential effect modifiers, including intervention duration and sarcopenia diagnosis or risk status, to explore possible sources of heterogeneity [28]. For outcomes with at least 10 studies and substantial heterogeneity, random-effects meta-regression was further used to examine the relationship between intervention duration and effect size [29]. Intervention duration was entered as a continuous variable in actual weeks, and between-study variance was estimated using restricted maximum likelihood (REML).

A comparison-level leave-one-out sensitivity analysis was conducted to evaluate the influence of each individual intervention-control comparison on the pooled effect esti-mate and between-study heterogeneity. The unit of analysis was each intervention-control comparison included in the pairwise meta-analysis. For multi-arm trials contributing more than one eligible comparison, each comparison was omitted separately, consistent with the way effect sizes were constructed in the pairwise meta-analysis [17]. In addition, a broader-classification sensitivity analysis was conducted. Nutritional interventions were regrouped into broader categories according to their core nutritional composition: protein-based interventions, protein + vitamin D-based interventions, and other nutritional interventions, with control retained as the reference category. Pairwise meta-analyses were then repeated for each outcome using these broader intervention categories, and the pooled effect estimates, 95% CIs, and *I*^2^ values were compared to those from the primary pairwise meta-analyses. A population-restriction sensitivity analysis was also conducted to assess whether the inclusion of participants at high risk of sarcopenia influenced the pooled estimates. This analysis was restricted to studies or study subgroups enrolling participants with confirmed sarcopenia, excluding those identified only as having probable sarcopenia, low muscle mass, or another high-risk condition without a confirmed diagnosis. Pairwise meta-analyses were repeated for each outcome using the same random-effects models as in the primary analyses, and changes in the pooled effect estimates, 95% CIs, and *I*^2^ values were examined relative to the primary analyses.

#### 2.6.2. Network Meta-Analysis

Stata 15.1 was used to construct evidence networks and perform NMA separately for ASMI, grip strength, gait speed, and 5STS using the network suite of commands. Continuous outcome data were set up using the network setup command with the smd option, and random-effects consistency models were fitted using network meta c within a multivariate meta-regression framework. Between-study heterogeneity was estimated using restricted maximum likelihood (REML), with a common between-study heterogeneity variance assumed across treatment comparisons within each outcome-specific network. Relative treatment effects were expressed as SMDs with 95% CIs, calculated using the normal approximation based on the model-estimated standard errors. Convergence of the REML estimation was verified from the iterative optimization output by confirming that the estimation reached a stable final log-likelihood and returned the complete set of model parameter estimates.

Network nodes were prespecified according to the specific composition of each nutritional intervention. Formulations with different core nutrient compositions were treated as separate intervention nodes to preserve the clinical interpretability of distinct nutritional strategies and to avoid masking potential effect differences through excessive pooling. Control groups receiving placebo, usual diet, or no additional nutritional supplementation were combined into a single Control node, provided that background conditions other than the target nutritional supplementation were identical or broadly comparable across randomized groups within each study. This node definition was based on the shared characteristic that no additional target nutritional supplement was provided and was used to maintain comparability and connectivity of control conditions across the network. The specific form of the control condition was also considered in the assessment of transitivity. The composition, dose and frequency, and contributing studies for each intervention node are presented in Supplementary Table S2. The transitivity assumption was assessed by examining the comparability of clinical and methodological characteristics across studies and intervention nodes [13,30]. Prespecified potential effect modifiers included participant age, sarcopenia status, intervention duration, and background exercise. Their distributions were examined across treatment comparisons within each outcome-specific network to identify potential systematic differences that could affect the validity of indirect comparisons. Detailed assessments of these effect modifiers are presented in Supplementary Tables S3–S6. Sex distribution, supplement dose, administration frequency, adherence, and the specific form of the control condition were additionally reviewed at the study level using Supplementary Table S1 and were considered when evaluating residual clinical heterogeneity. Global inconsistency was assessed using an inconsistency model implemented with network meta i, and local inconsistency was examined using node-splitting analyses with network sidesplit all. For networks containing closed loops, loop-specific inconsistency was additionally examined using inconsistency-factor plots. Treatment ranking probabilities were estimated using network rank max with 10,000 repetitions. The resulting cumulative ranking probabilities were used to calculate the surface under the cumulative ranking curve (SUCRA) [31]. Because treatment rankings may be unstable in sparse networks, SUCRA values were interpreted as exploratory and considered together with effect estimates, 95% CIs, network structure, and certainty of evidence rather than as evidence of a definitive treatment hierarchy.

First, a sensitivity analysis excluding interventions represented by only one study within an outcome-specific network was conducted, and the NMA was repeated using the original formulation-specific node definitions. Second, a trial-level leave-one-out analysis was conducted for principal comparisons supported by more than one independent trial [32]. Each contributing parent trial was sequentially removed and the network was re-estimated, with changes in the effect estimates and 95% CIs examined for the corresponding principal comparisons. When a single parent trial contributed more than one mutually exclusive eligible subgroup, all subgroups from that trial were treated as originating from the same trial for the purpose of the influence analysis. Comparisons involving nodes supported by only one independent parent trial were not subjected to this analysis because removal of that trial would eliminate the corresponding node from the network. Third, because the definition and grouping of intervention nodes may influence NMA estimates, an alternative node-classification sensitivity analysis was performed using a broader lumping approach [15]. Clinically comparable nutritional interventions with similar core nutritional characteristics were regrouped into broader intervention categories; the networks were re-estimated using these alternative node definitions, and changes in the direction, magnitude, and uncertainty of the effect estimates were compared with those from the primary formulation-specific NMA. Finally, a population-restriction sensitivity analysis was conducted to assess the influence of population definition on the network estimates. For each outcome, the analysis was restricted to studies or study subgroups enrolling participants with confirmed sarcopenia, excluding populations identified only as having probable sarcopenia, low muscle mass, or another high-risk condition without a confirmed diagnosis. The outcome-specific networks were reconstructed using the original formulation-specific node definitions. Restricted NMAs were performed when the remaining evidence formed a connected network that permitted estimation of relative treatment effects. Where estimation was feasible, changes in the direction, magnitude, and uncertainty of the principal network estimates were compared with those from the primary NMA. If restriction to confirmed sarcopenia resulted in insufficient network connectivity, a restricted NMA was not performed for that outcome.

### 2.7. Publication Bias

For outcomes with at least 10 included studies, comparison-adjusted funnel plots were used to explore potential small-study effects or publication bias [17].

### 2.8. Certainty of Evidence

The CINeMA (Confidence in Network Meta-Analysis) framework was used to assess the certainty of evidence for the NMA results for each outcome. CINeMA evaluates six domains: within-study bias, reporting bias, indirectness, imprecision, heterogeneity, and incoherence. Each domain was rated as no concerns, some concerns, or major concerns. After integrating judgments across domains, the overall certainty for each network comparison was rated as very low, low, moderate, or high, and the specific reasons for downgrading were recorded [33].

Indirectness was judged primarily according to the extent to which study popula-tions, interventions, comparators, outcomes, and potential effect modifiers matched the target research question. The absence of direct head-to-head comparisons between key interventions was not, by itself, considered equivalent to indirectness. Instead, network sparsity, limited evidence for key nodes, and the resulting wide confidence intervals were considered primarily as factors affecting imprecision and the stability of network estimates. Two reviewers (XC and SF) independently assessed the certainty of evidence. Disagreements were resolved through discussion. If consensus could not be reached, a third reviewer (XZ) participated in the discussion and made the final decision.

## 3. Results

### 3.1. Study Selection

**Figure 1:**
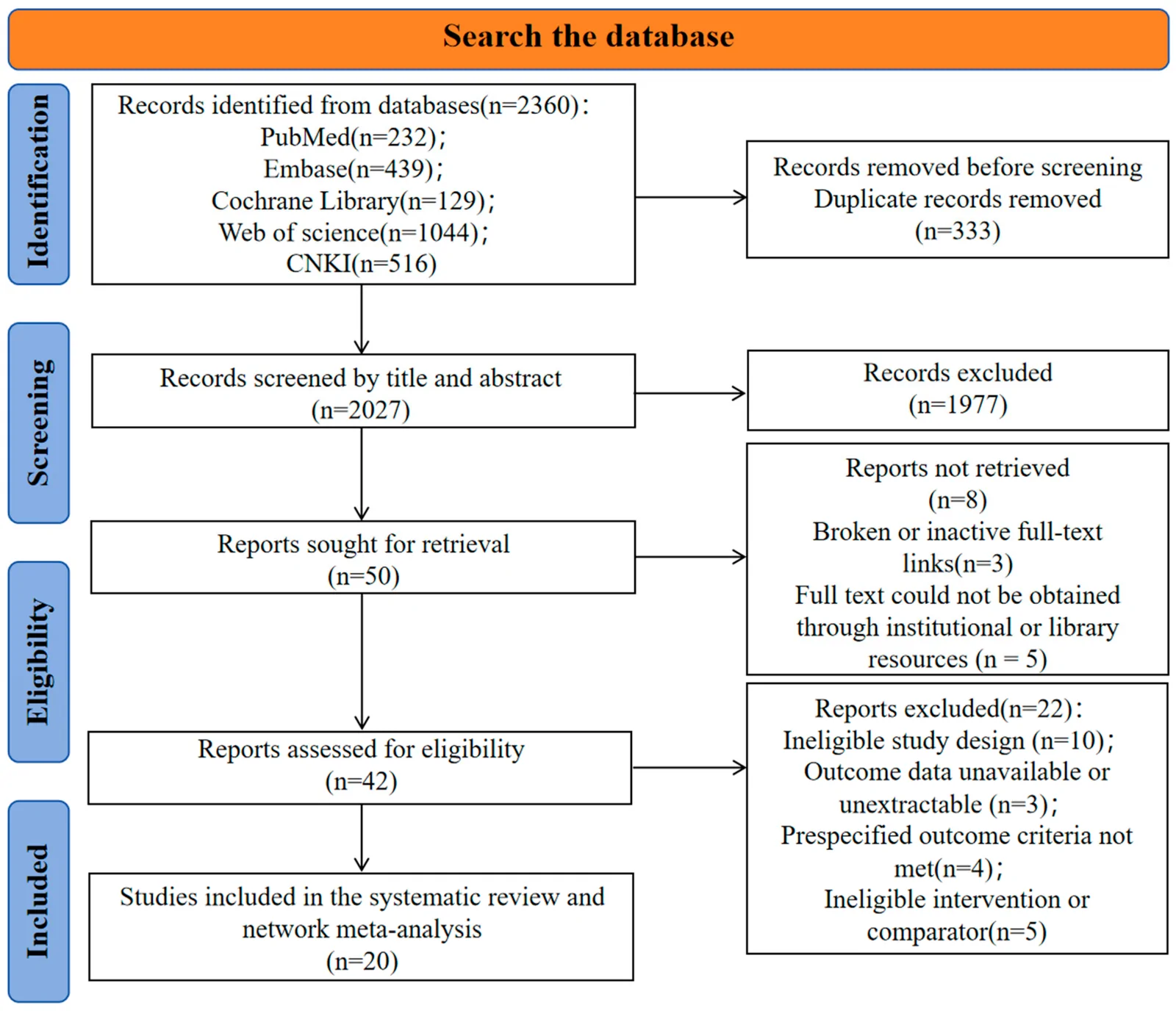
PRISMA flow diagram of study selection.

Database searches and supplementary searches identified 2360 records. After re-moval of 333 duplicates, 2027 records underwent title and abstract screening, of which 1977 were excluded. Full-text retrieval was attempted for 50 reports; 8 could not be re-trieved, leaving 42 reports for full-text eligibility assessment. After full-text assessment, 22 reports were excluded, mainly because of an ineligible study design (*n* = 10), unavailable or non-extractable outcome data (*n* = 3), failure to meet prespecified outcome criteria (*n* = 4), or ineligible intervention or comparator conditions (*n* = 5). Ultimately, 20 randomized controlled trials (RCTs) were included in the systematic review and meta-analysis [34,35,36,37,38,39,40,41,42,43,44,45,46,47,48,49,50,51,52,53]. The study selection process is shown in Figure 1.

### 3.2. Characteristics of Included Studies

A total of 20 RCTs involving 2160 participants were included. Five studies were multi-arm randomized controlled trials, and the remainder were two-arm trials. All in-cluded studies enrolled adults aged ≥60 years and included both participants with confirmed sarcopenia and populations at high risk of sarcopenia as defined by the original studies. Sarcopenia or the related risk status was primarily determined according to AWGS, EWGSOP, or prespecified criteria used in the original study. Intervention dura-tion ranged from 6 to 48 weeks.

Based on the specific nutritional composition, 17 network nodes were defined, in-cluding 16 active nutritional intervention strategies and one control node. Active inter-ventions mainly involved protein or whey protein, leucine, vitamin D, omega-3 fatty acids, HMB, calcium, probiotics, and various multi-nutrient combinations. Control conditions included placebo, usual diet, or no additional nutritional supplementation. The basic characteristics of the included studies are presented in Table 1. Detailed nutritional composition, participant age and sex, sarcopenia diagnostic criteria, baseline population characteristics, intervention dose and frequency, co-interventions, intervention duration, and adherence are provided in Supplementary Table S1. The composition, dose and fre-quency, and contributing studies for each intervention node are provided in Supplementary Table S2. Studies in Table 1 and Supplementary Table S1 are arranged chronologically by publication year.

### 3.3. Risk of Bias

**Figure 2:**
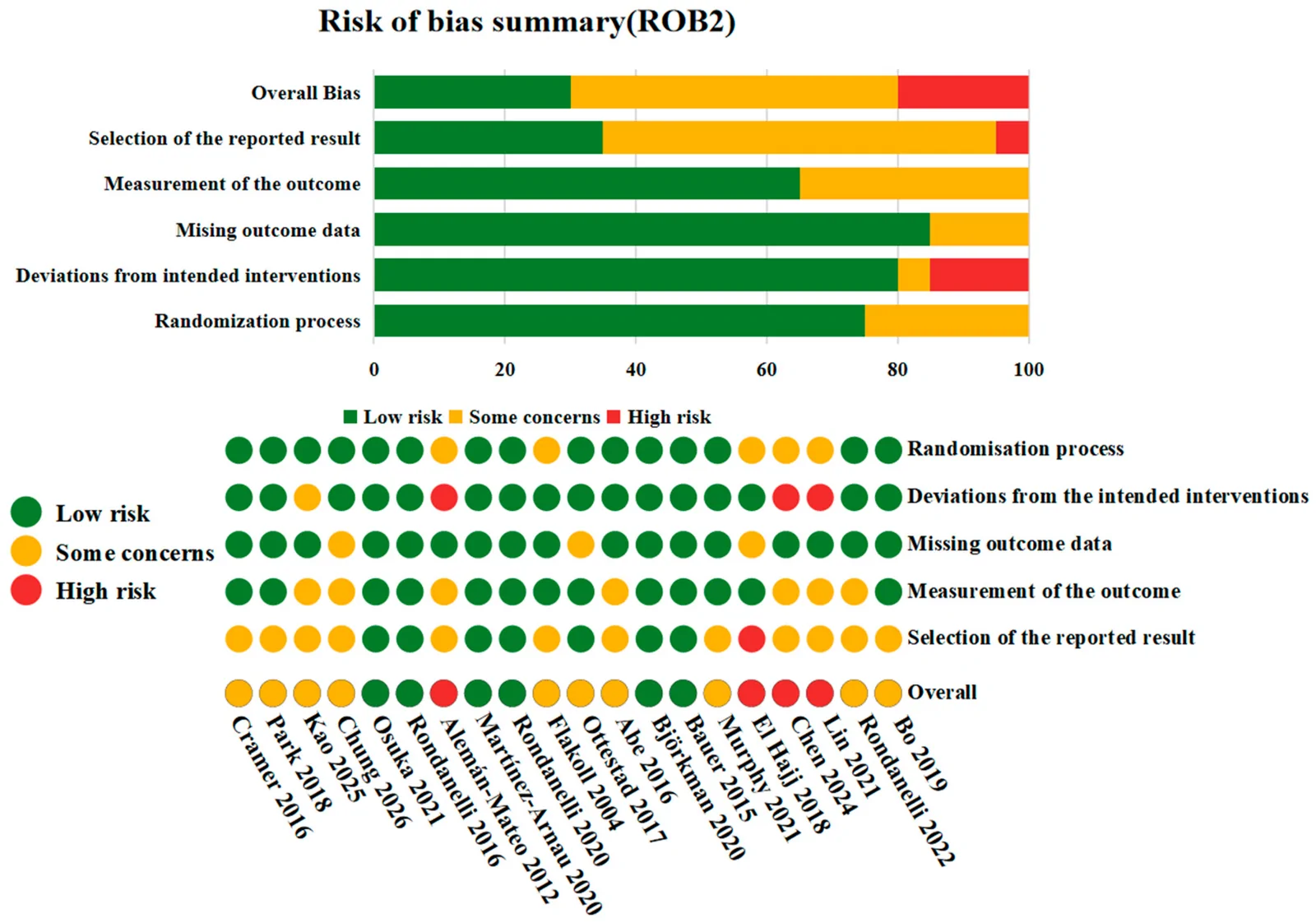
Risk-of-bias assessment of the included randomized controlled trials [34,35,36,37,38,39,40,41,42,43,44,45,46,47,48,49,50,51,52,53].

Risk of bias was assessed using the Cochrane RoB 2 tool. For the randomization process, five studies raised some concerns [36,37,39,41,44]. For deviations from intended interventions, three studies were judged to be at high risk [36,37,39], and one study raised some concerns [48]. For missing outcome data, three studies raised some concerns [41,42,46]. For outcome measurement, seven studies raised some concerns [36,37,38,39,42,48,49]. For selection of the reported result, one study was judged to be at high risk [41], and 12 studies raised some concerns [36,37,38,40,42,43,44,47,48,49,50,51]. Overall, six studies were judged to have a low risk of bias [34,35,43,45,52,53], 10 studies raised some concerns [38,40,42,44,46,47,48,49,50,51], and four studies were judged to have a high risk of bias [36,37,39,41]. Detailed assessments are shown in Figure 2.

### 3.4. Pairwise Meta-Analysis

For muscle mass, pooled results from nine studies showed that, compared to control, nutritional interventions significantly improved ASMI (SMD = 0.25, 95% CI: 0.10 to 0.40, *p* = 0.001), with low between-study heterogeneity (*I*^2^ = 0%). For muscle strength, pooled results from 17 studies showed that nutritional interventions significantly improved grip strength (SMD = 0.42, 95% CI: 0.15 to 0.68, *p* = 0.002), although between-study heterogeneity was high (*I*^2^ = 85%). For physical performance, 11 studies showed a significant improvement in gait speed (SMD = 0.27, 95% CI: 0.08 to 0.46, *p* = 0.006, *I*^2^ = 65%), whereas pooled results from eight studies showed no statistically significant effect on 5STS (SMD = −0.31, 95% CI: −0.81 to 0.20, *p* = 0.23, *I*^2^ = 91%). Detailed results are presented in Supplementary Figures S1–S4.

In subgroup analyses by intervention duration, grip strength improved significantly in the <12-week and 12-week subgroups, whereas no significant difference was observed in the >12-week subgroup. Gait speed and 5STS showed larger effects in the <12-week subgroup, but each subgroup was supported by only one study, limiting the stability and generalizability of the findings. Further subgroup analyses according to sarcopenia diagnostic criteria and baseline risk status showed no clear differences between subgroups, and between-study heterogeneity was not consistently reduced. Given the small and uneven numbers of studies in some subgroups, these findings were interpreted as exploratory. Detailed results are shown in Supplementary Figures S2–S4.

For grip strength and gait speed, both of which included at least 10 studies and showed substantial heterogeneity, random-effects meta-regression was further used to explore the relationship between intervention duration and effect size. No significant association was found between intervention duration and the effect size for grip strength (*p* = 0.072) or gait speed (*p* = 0.388) (Supplementary Tables S7 and S8).

### 3.5. Network Meta-Analysis

#### 3.5.1. Evidence Network

Based on Table 1 and Supplementary Tables S1 and S2, the distribution of potential effect modifiers across different direct comparisons was assessed. Overall, age, sarcopenia diagnosis or baseline risk status, and intervention duration were broadly comparable across many treatment comparisons, although the degree of overlap varied across outcome-specific networks. Background exercise or rehabilitation, nutritional supplement dose, administration frequency, and adherence showed greater variation across studies, and some factors were inadequately reported. Sex distribution was additionally reviewed at the study level and did not indicate an obvious systematic imbalance across the evidence base. Accordingly, the transitivity assumption was considered reasonably plausible rather than established, with residual clinical heterogeneity and uncertainty remaining. Further assessment of prespecified potential effect modifiers across indirect comparisons showed broadly comparable participant ages and overlapping distributions of sarcopenia status, intervention duration, and background exercise across intervention nodes, although the degree of overlap varied among comparisons (Supplementary Tables S3–S6). Accordingly, the transitivity assumption was considered reasonably plausible, although residual clinical heterogeneity and uncertainty remained.

**Figure 3:**
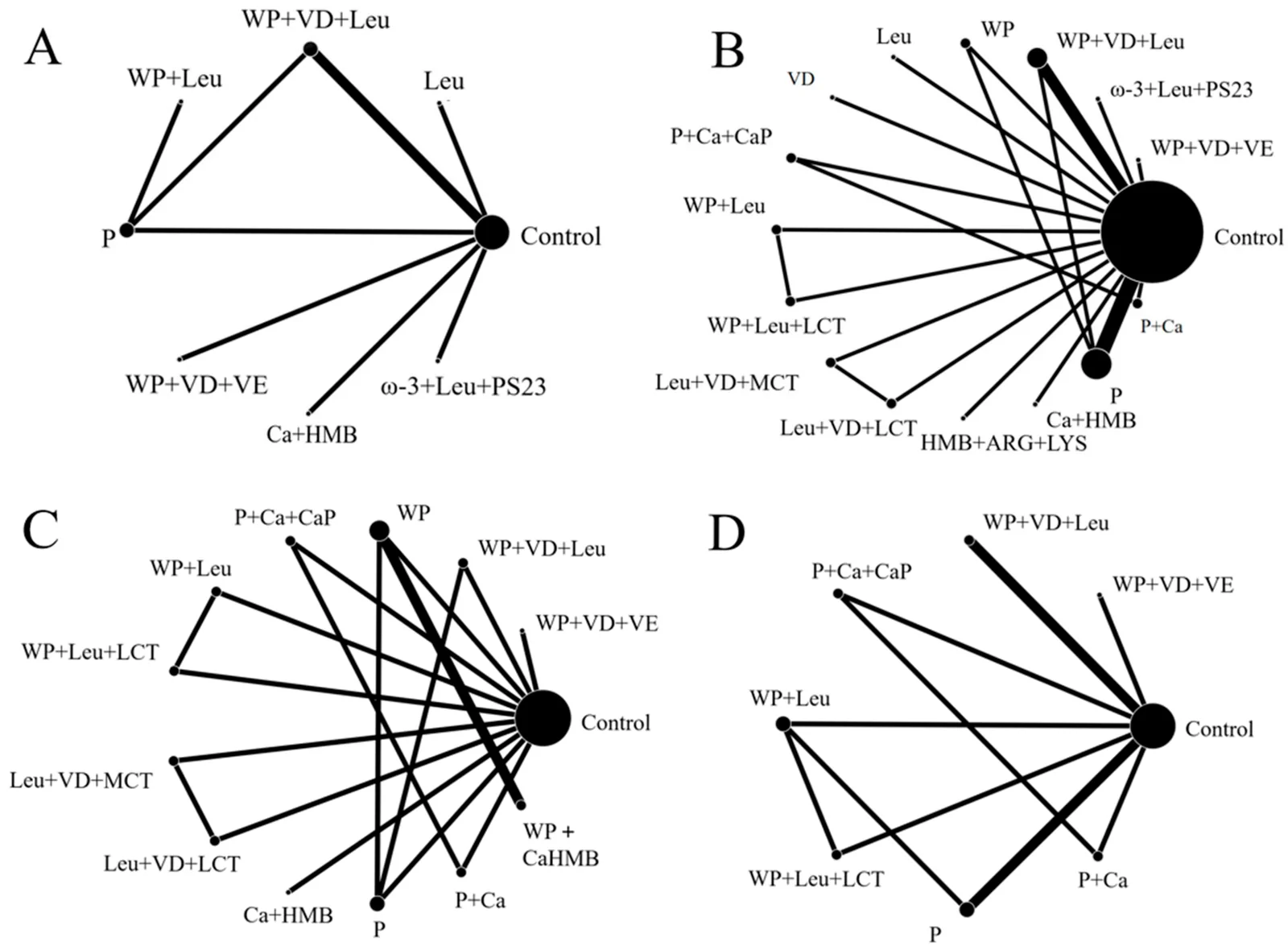
Evidence networks for outcomes. Notes: Panel (**A**) for appendicular skeletal muscle mass index (ASMI), Panel (**B**) for grip strength, Panel (**C**) for gait speed, and Panel (**D**) for five-times sit-to-stand test (5STS). Each node represents a specific nutritional formulation or combination as defined a priori in Supplementary Table S2, with node size proportional to the sample size. Lines connecting circles indicate direct comparisons between two strategies, with line thickness proportional to the number of studies. Control represents the control group receiving placebo, usual diet, or no additional nutritional supplementation. WP+VD+VE, whey protein + vitamin D + vitamin E; ω-3+Leu+PS23, ω-3 fatty acids + leucine + Lactobacillus paracasei PS23; WP+VD+Leu, whey protein + vitamin D + leucine; WP, whey protein; Leu, leucine; VD, vitamin D; P+Ca, protein + calcium; P+Ca+CaP, protein + calcium + calcium-containing phosphates; WP+Leu, whey protein + leucine; WP+Leu+LCT, whey protein + leucine + long-chain lipid formulation containing *n*-3 PUFA; Leu+VD+MCT, leucine + vitamin D + medium-chain triglycerides; Leu+VD+LCT, leucine + vitamin D + long-chain triglycerides; HMB+ARG+LYS, β-hydroxy-β-methylbutyrate + arginine + lysine; Ca+HMB, calcium + β-hydroxy-β-methylbutyrate; P, protein; WP+CaHMB, whey protein + calcium β-hydroxy-β-methylbutyrate.

Global inconsistency tests detected no statistically significant inconsistency in the networks for ASMI (χ^2^ = 0.0001, *df* = 1, *p* = 0.9999), grip strength (χ^2^ = 1.46, *df* = 2, *p* = 0.483), gait speed (χ^2^ = 0.09, *df* = 1, *p* = 0.758), or 5STS (χ^2^ = 0.19, *df* = 1, *p* = 0.663). Loop-specific analyses likewise showed no statistically significant inconsistency for ASMI (IF = 0.000, *p* = 1.000), grip strength (IF = 0.550, 95% CI: −0.22 to 1.32, *p* = 0.256; IF = 0.083, *p* = 0.792), gait speed (IF = 0.064, *p* = 0.865), or 5STS (IF = 0.710, *p* = 0.108). In addition, node-splitting analyses yielded *p* > 0.05 for the relevant comparisons in all outcome networks, with no statistically significant local inconsistency detected. Because some networks were sparse, these nonsignificant findings cannot completely exclude potential inconsistency. Therefore, the main network effects were estimated using a random-effects consistency model under the assumption of reasonably plausible transitivity and were interpreted cautiously in light of the network structure. Figure 3 displays the network plots for each outcome.

#### 3.5.2. ASMI

Nine studies involving 685 participants reported ASMI. NMA showed that, compared to control, whey protein + vitamin D + leucine significantly improved ASMI (SMD = 0.29, 95% CI: 0.06 to 0.52), and omega-3 fatty acids + leucine + Lactobacillus paracasei PS23 also showed a significant improvement (SMD = 0.67, 95% CI: 0.10 to 1.25). No statistically significant differences were observed between the other nutritional strategies and control. Detailed network estimates are shown in Supplementary Figure S5.

**Figure 4:**
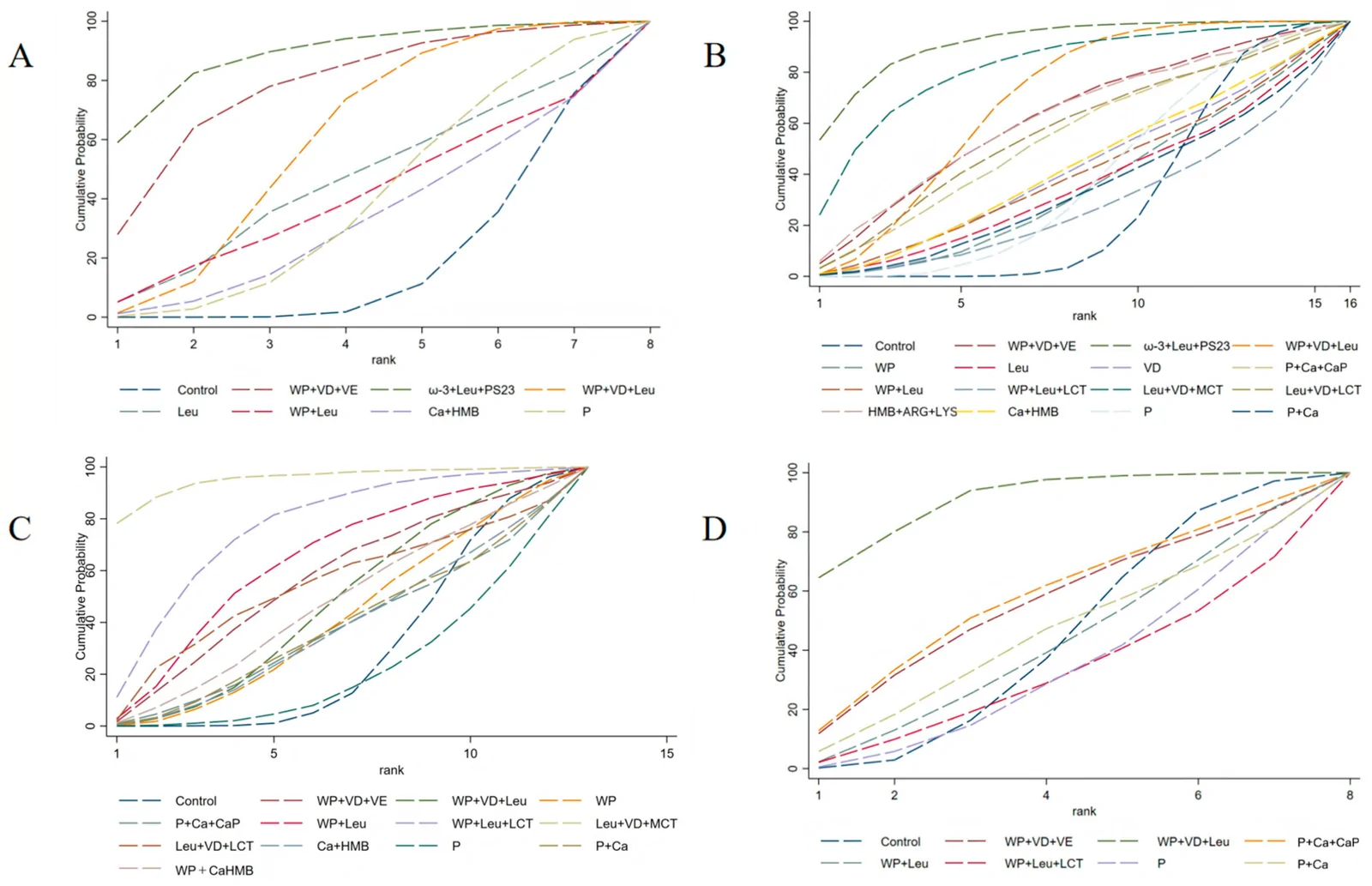
SUCRA ranking curves for outcomes. Notes: Panel (**A**) for appendicular skeletal muscle mass index (ASMI), Panel (**B**) for grip strength, Panel (**C**) for gait speed, and Panel (**D**) for five-times sit-to-stand test (5STS). SUCRA summarizes each intervention’s relative position across the cumulative ranking distribution. A higher SUCRA value indicates a relatively higher position in the network ranking. The x-axis represents the possible rank positions, and the y-axis shows the corresponding cumulative probability (%). Control represents the control group receiving placebo, usual diet, or no additional nutritional supplementation. WP+VD+VE, whey protein + vitamin D + vitamin E; ω-3+Leu+PS23, ω-3 fatty acids + leucine + Lactobacillus paracasei PS23; WP+VD+Leu, whey protein + vitamin D + leucine; WP, whey protein; Leu, leucine; VD, vitamin D; P+Ca, protein + calcium; P+Ca+CaP, protein + calcium + calcium-containing phosphates; WP+Leu, whey protein + leucine; WP+Leu+LCT, whey protein + leucine + long-chain lipid formulation containing *n*-3 PUFA; Leu+VD+MCT, leucine + vitamin D + medium-chain triglycerides; Leu+VD+LCT, leucine + vitamin D + long-chain triglycerides; HMB+ARG+LYS, β-hydroxy-β-methylbutyrate + arginine + lysine; Ca+HMB, calcium + β-hydroxy-β-methylbutyrate; P, protein; WP+CaHMB, whey protein + calcium β-hydroxy-β-methylbutyrate.

SUCRA results showed that omega-3 fatty acids + leucine + Lactobacillus paracasei PS23 had the highest SUCRA value (88.2%), followed by whey protein + vitamin D + vitamin E (78.4%) and whey protein + vitamin D + leucine (60.8%). These values reflect the relative ranking positions of the interventions within the current network rather than the probability of being the best intervention. Because some highly ranked nodes were supported by only a single study, the rankings should be interpreted cautiously together with effect estimates, 95% CIs, and the certainty of evidence. SUCRA rankings are shown in Figure 4A.

#### 3.5.3. Grip Strength

Seventeen studies involving 1512 participants reported grip strength. Compared with control, whey protein + vitamin D + leucine significantly improved grip strength (SMD = 0.82, 95% CI: 0.13 to 1.50), and omega-3 fatty acids + leucine + Lactobacillus paracasei PS23 also showed a significant improvement (SMD = 1.84, 95% CI: 0.39 to 3.28). Between-intervention comparisons showed that omega-3 fatty acids + leucine + Lactobacillus paracasei PS23 was significantly more effective than protein supplementation alone (SMD = 1.68, 95% CI: 0.10 to 3.26). Detailed results are shown in Supplementary Figure S6.

SUCRA results showed that omega-3 fatty acids + leucine + Lactobacillus paracasei PS23 had the highest SUCRA value (91.6%), followed by vitamin D + leucine + medium-chain triglycerides (82.2%) and whey protein + vitamin D + leucine (69.9%). Because some highly ranked nodes were supported by only one study and had wide 95% CIs, SUCRA values were used only to describe relative ranking and should not be interpreted as definitive evidence of clinical superiority. SUCRA rankings are shown in Figure 4B.

#### 3.5.4. Gait Speed

Eleven studies involving 1242 participants reported gait speed. Compared to control, leucine + vitamin D + medium-chain triglycerides significantly improved gait speed (SMD = 1.28, 95% CI: 0.13 to 2.43). Between-intervention comparisons showed that leucine + vitamin D + medium-chain triglycerides was significantly more effective than protein supplementation alone (SMD = 1.37, 95% CI: 0.11 to 2.62). No statistically significant differences were observed for the other comparisons. Detailed results are shown in Supplementary Figure S7.

SUCRA results showed that leucine + vitamin D + medium-chain triglycerides had the highest SUCRA value (94.6%), followed by whey protein + leucine + long-chain lipid supplementation (77.5%) and whey protein + leucine (64.2%). Because the leucine + vitamin D + medium-chain triglycerides node was supported by only one study and had a wide 95% CI, the SUCRA ranking should be interpreted cautiously and should not be regarded as definitive evidence of clinical superiority. SUCRA rankings are shown in Figure 4C.

#### 3.5.5. 5STS

Eight studies involving 778 participants reported 5STS. Because a shorter completion time indicates better physical performance, a negative SMD represents a favorable effect. NMA showed that, compared with control, whey protein + vitamin D + leucine significantly reduced 5STS completion time (SMD = −1.41, 95% CI: −2.63 to −0.18). No statistically significant differences were observed between the other nutritional strategies and control. Detailed results are shown in Supplementary Figure S8.

SUCRA results showed that whey protein + vitamin D + leucine had the highest SUCRA value (91.6%), followed by protein + calcium + calcium-containing phosphates (56.5%) and whey protein + vitamin D + vitamin E (55.2%). Given the relatively sparse evidence network, these ranking results should still be interpreted cautiously together with effect estimates, 95% CIs, and the certainty of evidence. SUCRA rankings are shown in Figure 4D.

### 3.6. Sensitivity Analysis

#### 3.6.1. Sensitivity Analysis for Pairwise Meta-Analysis

Comparison-level leave-one-out sensitivity analyses showed that the pooled effect for grip strength was generally stable, with no material change in effect direction or statistical significance after removal of any individual comparison. For gait speed, after removal of the comparison contributed by Rondanelli (2020) [52], between-study heterogeneity decreased from 65% to 0%, while the pooled effect remained statistically significant (SMD = 0.16, 95% CI: 0.05 to 0.27, *p* = 0.004), although the effect size was smaller than in the primary analysis. For 5STS, after removal of the comparison from the same study, heterogeneity decreased from 91% to 51%, and the pooled effect remained non-significant (SMD = −0.08, 95% CI: −0.32 to 0.15, *p* = 0.48). Detailed results are presented in Supplementary Tables S9–S11.

When nutritional interventions were regrouped into broader categories, the pooled effect estimates were generally consistent with those of the primary pairwise meta-analyses. For ASMI, the pooled effect changed from an SMD of 0.25 (95% CI: 0.10 to 0.40; *I*^2^ = 0%) in the primary analysis to 0.25 (95% CI: 0.10 to 0.41; *I*^2^ = 0%) in the broader-classification sensitivity analysis. For grip strength, the corresponding estimate changed from 0.42 (95% CI: 0.15 to 0.68; *I*^2^ = 85%) to 0.47 (95% CI: 0.15 to 0.80; *I*^2^ = 87.7%). For gait speed, the estimate changed from 0.27 (95% CI: 0.08 to 0.46; *I*^2^ = 65%) to 0.19 (95% CI: 0.05 to 0.33; *I*^2^ = 7%). For 5STS, the estimate changed from −0.31 (95% CI: −0.81 to 0.20; *I*^2^ = 91%) to −0.41 (95% CI: −1.06 to 0.24; *I*^2^ = 93.7%). Thus, the direction and statistical significance of the pooled effects were unchanged across all four outcomes after broader classification, although heterogeneity was substantially reduced for gait speed. Detailed results are presented in Supplementary Table S12.

In the population-restriction sensitivity analysis restricted to participants with confirmed sarcopenia, the pooled effect for ASMI was 0.31 (95% CI: 0.13 to 0.49; *p* < 0.001; *I*^2^ = 0%), compared with 0.25 (95% CI: 0.10 to 0.40; *p* = 0.001; *I*^2^ = 0%) in the primary analysis. For grip strength, the pooled effect was 0.54 (95% CI: 0.11 to 0.96; *p* = 0.01; *I*^2^ = 91%), compared with 0.42 (95% CI: 0.15 to 0.68; *p* = 0.002; *I*^2^ = 85%) in the primary analysis. For gait speed, the pooled effect was 0.30 (95% CI: −0.11 to 0.71; *p* = 0.15; *I*^2^ = 86%), compared to 0.27 (95% CI: 0.08 to 0.46; *p* = 0.006; *I*^2^ = 65%) in the primary analysis. For 5STS, the pooled effect was −0.90 (95% CI: −1.88 to 0.07; *p* = 0.07; *I*^2^ = 95%), compared with −0.31 (95% CI: −0.81 to 0.20; *p* = 0.23; *I*^2^ = 91%) in the primary analysis. Detailed results are presented in Supplementary Table S13.

#### 3.6.2. Sensitivity Analysis for Network Meta-Analysis

To assess whether the network results were influenced by nodes supported by a single study, intervention nodes supported by only one study were excluded and the network for each outcome was reconstructed. For nodes supported by multiple studies and retained after the sensitivity analysis, effect directions were generally consistent with the primary analysis, although some SUCRA values changed. Several highly ranked single-study nodes in the primary analysis were no longer present after exclusion. Therefore, this sensitivity analysis could not verify the robustness of their apparent advantages. In the ASMI network, before sensitivity analysis, whey protein + vitamin D + leucine had an SMD of 0.29 (95% CI: 0.06 to 0.52; SUCRA = 60.8%) and protein had an SMD of 0.14 (95% CI: −0.19 to 0.47; SUCRA = 38.5%). After sensitivity analysis, the corresponding estimates were 0.29 (95% CI: 0.05 to 0.54; SUCRA = 89.7%) and 0.14 (95% CI: −0.26 to 0.54; SUCRA = 50.2%), respectively. In the grip-strength network, before sensitivity analysis, whey protein + vitamin D + leucine had an SMD of 0.82 (95% CI: 0.13 to 1.50; SUCRA = 69.9%) and protein had an SMD of 0.16 (95% CI: −0.47 to 0.80; SUCRA = 39.1%). After sensitivity analysis, the corresponding estimates were 0.83 (95% CI: 0.08 to 1.58; SUCRA = 94.2%) and 0.22 (95% CI: −0.56 to 0.99; SUCRA = 40.5%), respectively. In the gait-speed network, before sensitivity analysis, whey protein + vitamin D + leucine had an SMD of 0.17 (95% CI: −0.25 to 0.60; SUCRA = 48.7%), whey protein + calcium β-hydroxy-β-methylbutyrate had an SMD of 0.16 (95% CI: −0.53 to 0.86; SUCRA = 47.4%), and whey protein had an SMD of 0.11 (95% CI: −0.47 to 0.69; SUCRA = 46.2%). After sensitivity analysis, the corresponding estimates were 0.17 (95% CI: −0.51 to 0.84; SUCRA = 61.5%), 0.17 (95% CI: −0.75 to1.09; SUCRA = 59.7%), and 0.11 (95% CI: −0.96 to 1.17; SUCRA = 34.5%), respectively. In the 5STS network, before sensitivity analysis, whey protein + vitamin D + leucine had an SMD of −1.41 (95% CI: −2.63 to −0.18; SUCRA = 91.6%), whey protein + leucine had an SMD of 0.04 (95% CI: −1.35 to 1.42; SUCRA = 43.3%), and protein had an SMD of 0.19 (95% CI: −0.94 to 1.31; SUCRA = 34.6%). After sensitivity analysis, the corresponding estimates were −1.41 (95% CI: −2.64 to −0.18; SUCRA = 96.6%), 0.04 (95% CI: −1.35 to 1.42; SUCRA = 37.1%), and 0.19 (95% CI: −0.94 to 1.31; SUCRA = 27.4%), respectively. Supplementary Tables S14–S17 provide detailed results.

Trial-level leave-one-out analyses showed that the direction of the principal network estimates was generally maintained, although the statistical significance of some estimates was sensitive to the exclusion of individual trials. For ASMI, the estimate for whey protein + vitamin D + leucine versus control changed from 0.29 (95% CI: 0.06 to 0.52) to 0.20 (95% CI: −0.11 to 0.51) after exclusion of Rondanelli et al. (2020) [52], whereas exclusion of the other contributing trials did not materially alter the result. For grip strength, the corresponding estimate changed from 0.82 (95% CI: 0.13 to 1.50) to 0.40 (95% CI: −0.08 to 0.88) after exclusion of Rondanelli et al. (2020) [52] and to 0.72 (95% CI: −0.14 to 1.59) after exclusion of Rondanelli et al. (2016) [53]. For gait speed, exclusion of individual contributing trials did not materially alter the nonsignificant estimate for whey protein + vitamin D + leucine versus control. For 5STS, the direction and statistical significance of the estimate for whey protein + vitamin D + leucine versus control were maintained across trial exclusions, although the magnitude of the effect varied. Detailed results are presented in Supplementary Tables S18–S21.

In the alternative node-classification sensitivity analysis, formulation-specific interventions were regrouped according to their core nutritional composition into protein-based interventions, protein + vitamin D-based interventions, and other nutritional interventions, with control retained as the reference node. For ASMI, protein + vitamin D-based interventions significantly improved ASMI compared with control (SMD = 0.33, 95% CI: 0.12 to 0.54), whereas no significant differences were observed between the broader intervention categories. For grip strength, protein + vitamin D-based interventions (SMD = 0.80, 95% CI: 0.27 to 1.32) and other nutritional interventions (SMD = 0.62, 95% CI: 0.11 to 1.13) significantly improved grip strength compared with control; protein + vitamin D-based interventions were also significantly more effective than protein-based interventions (SMD = 0.65, 95% CI: 0.02 to 1.29). For gait speed, protein + vitamin D-based interventions significantly improved gait speed compared with control (SMD = 0.22, 95% CI: 0.02 to 0.41), whereas the remaining comparisons were not statistically significant. For 5STS, regrouping the interventions did not yield a network structure suitable for NMA; therefore, an alternative broader-node NMA was not performed for this outcome. The alternative classification of intervention nodes is presented in Supplementary Table S22, the complete network estimates are provided in Supplementary Table S23, and the corresponding network plots are shown in Supplementary Figure S9.

In the population-restriction sensitivity analysis restricted to participants with confirmed sarcopenia, whey protein + vitamin D + leucine remained significantly associated with higher ASMI compared with control (SMD = 0.29, 95% CI: 0.05 to 0.54), similar to the primary NMA (SMD = 0.29, 95% CI: 0.06 to 0.52). ω-3 fatty acids + leucine + Lactobacillus paracasei PS23 also remained significant compared with control (SMD = 0.67, 95% CI: 0.10 to 1.25), with the same estimate as in the primary NMA. For grip strength, ω-3 fatty acids + leucine + Lactobacillus paracasei PS23 remained significant compared with control (SMD = 1.84, 95% CI: 0.02 to 3.65), compared with an SMD of 1.84 (95% CI: 0.39 to 3.28) in the primary NMA. Whey protein + vitamin D + leucine versus control was no longer statistically significant after population restriction (SMD = 0.91, 95% CI: −0.01 to 1.84), compared with 0.82 (95% CI: 0.13 to 1.50) in the primary NMA. Similarly, the comparison of protein supplementation alone versus ω-3 fatty acids + leucine + Lactobacillus paracasei PS23 changed from −1.68 (95% CI: −3.26 to −0.10) in the primary NMA to −1.28 (95% CI: −3.54 to 0.98) in the population-restricted NMA. For gait speed, no statistically significant differences were observed among the interventions retained in the population-restricted network; the leucine + vitamin D + medium-chain triglycerides node, which was significant versus control in the primary NMA, was no longer represented after population restriction. For 5STS, no statistically significant differences were observed in the population-restricted NMA; whey protein + vitamin D + leucine versus control changed from −1.41 (95% CI: −2.63 to −0.18) in the primary NMA to −1.42 (95% CI: −3.42 to 0.58). Complete results are presented in Supplementary Tables S24–S27.

### 3.7. Publication Bias

**Figure 5:**
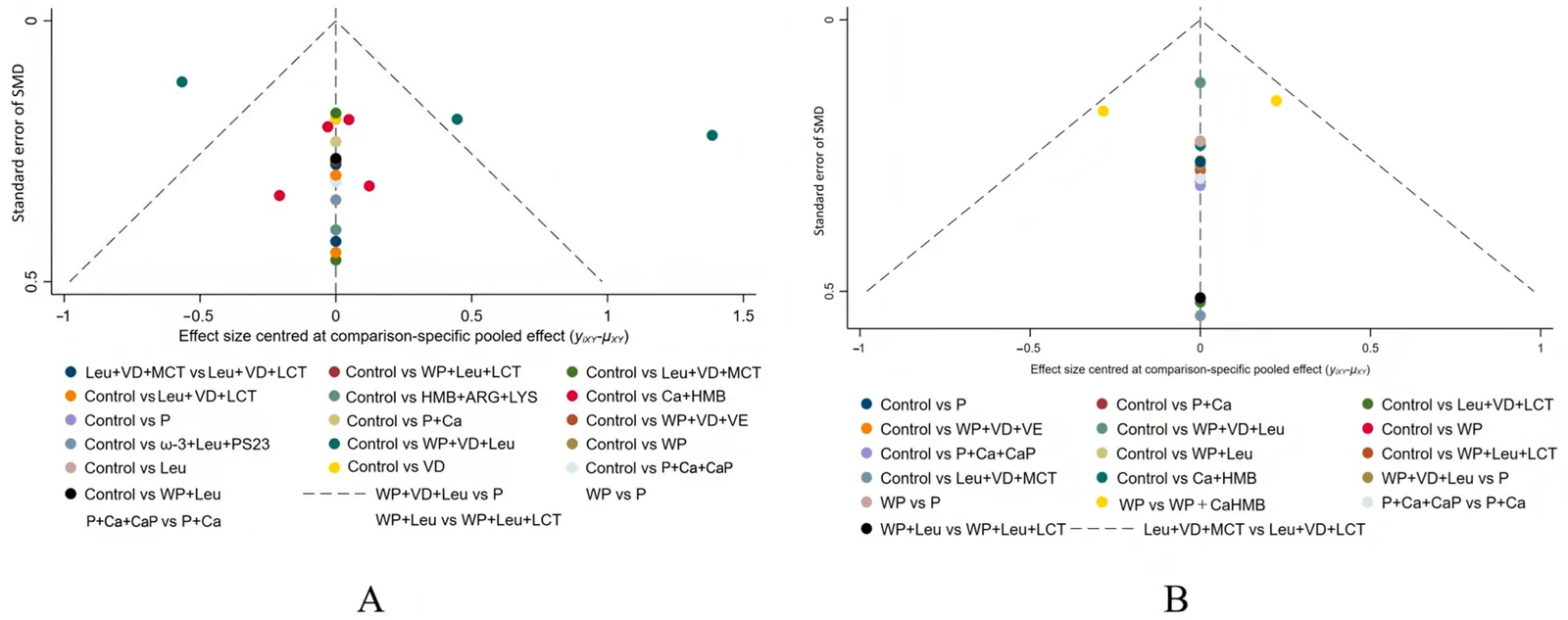
Comparison adjusted funnel plots for publication bias assessment. Notes: Panel (**A**) for grip strength, and Panel (**B**) for gait speed. Each point represents a comparison included in the network meta-analysis. The vertical dashed line represents the comparison-specific pooled effect, and the two diagonal dashed lines indicate the approximate 95% confidence limits. Asymmetry in the funnel plots may indicate possible small-study effects or other sources of between-study asymmetry and should not be interpreted as definitive evidence of publication bias. Abbreviations: Control represents the control group receiving placebo, usual diet, or no additional nutritional supplementation. WP+VD+VE, whey protein + vitamin D + vitamin E; ω-3+Leu+PS23, ω-3 fatty acids + leucine + Lactobacillus paracasei PS23; WP+VD+Leu, whey protein + vitamin D + leucine; WP, whey protein; Leu, leucine; VD, vitamin D; P+Ca, protein + calcium; P+Ca+CaP, protein + calcium + calcium-containing phosphates; WP+Leu, whey protein + leucine; WP+Leu+LCT, whey protein + leucine + long-chain lipid formulation containing *n*-3 PUFA; Leu+VD+MCT, leucine + vitamin D + medium-chain triglycerides; Leu+VD+LCT, leucine + vitamin D + long-chain triglycerides; HMB+ARG+LYS, β-hydroxy-β-methylbutyrate + arginine + lysine; Ca+HMB, calcium + β-hydroxy-β-methylbutyrate; P, protein; WP+CaHMB, whey protein + calcium β-hydroxy-β-methylbutyrate.

Comparison-adjusted funnel plots were generated for grip strength (17 studies) and gait speed (11 studies), the two outcomes with at least 10 included studies. Both funnel plots showed some degree of asymmetry, suggesting possible small-study effects or potential publication bias. Because funnel-plot asymmetry may also be influenced by between-study heterogeneity and differences among interventions, these findings were interpreted as exploratory. The comparison-adjusted funnel plots are shown in Figure 5.

### 3.8. Certainty of Evidence

CINeMA assessment showed that, across all included network comparisons, the certainty of evidence was rated as very low for the vast majority and as low for only a small number of comparisons. Downgrading was mainly related to within-study bias, imprecision, heterogeneity, and, for some comparisons, reporting bias or indirectness. Indirectness primarily reflected differences between the available evidence and the target research question in terms of populations, interventions, comparators, outcomes, and potential effect modifiers. In contrast, the absence of direct head-to-head comparisons between key nutritional strategies and network sparsity mainly increased imprecision and overall uncertainty and should not simply be equated with indirectness.

For the seven principal intervention–outcome comparisons highlighted in the Results and forming the main basis of the Discussion, the certainty of evidence ranged from low to very low. The comparisons of WP+VD+Leu versus Control for ASMI and 5STS were rated as low certainty, whereas the remaining principal comparisons were rated as very low certainty. A summary of the effect estimates, evidence base, six CINeMA domains, and overall confidence ratings for these principal comparisons is provided in Supplementary Table S28.

Comparison-adjusted funnel plots for grip strength and gait speed showed some asymmetry, suggesting possible small-study effects or reporting bias. Because fewer than 10 studies were available for ASMI and 5STS, funnel-plot asymmetry was not formally assessed for these outcomes. Detailed CINeMA assessments for all network comparisons across the four outcomes are presented in Supplementary Tables S29–S32.

## 4. Discussion

This study synthesized 20 RCTs to compare the relative effects of specific nutritional formulations on sarcopenia-related outcomes of muscle mass, muscle strength, and physical performance. Pairwise meta-analysis showed statistically significant overall effects of nutritional supplementation on ASMI, grip strength, and gait speed, whereas the pooled effect on 5STS was not statistically significant. The NMA further identified outcome-specific effects across nutritional formulations. Whey protein + vitamin D + leucine showed statistically significant effects versus control for ASMI, grip strength, and 5STS. Omega-3 fatty acids + leucine + Lactobacillus paracasei PS23 showed statistically significant effects on ASMI and grip strength, whereas leucine + vitamin D + medium-chain triglycerides showed a statistically significant effect on gait speed; however, both formulations were represented by single-study nodes. The certainty of evidence for the principal comparisons ranged from low to very low, and the findings should therefore be interpreted primarily on the basis of the effect estimates, their 95% CIs, and the underlying evidence base, with SUCRA rankings providing complementary exploratory information.

The effects observed for whey protein + vitamin D + leucine across multiple outcomes are biologically plausible. Older adults exhibit varying degrees of anabolic resistance, and simply increasing protein intake may not fully stimulate muscle protein synthesis. Whey protein is rich in essential amino acids and leucine, while leucine itself is an important nutritional signal for stimulating muscle protein synthesis [54,55]. Vitamin D may further influence muscle function through pathways related to muscle-cell function, mi-tochondrial metabolism, and neuromuscular performance [54,56]. These components may influence muscle-related outcomes through distinct physiological pathways [57]. This interpretation is broadly consistent with previous systematic reviews of leucine-enriched protein, whey protein, and vitamin D co-supplementation [9,11,54]. In this context, the favorable ASMI finding indicates an increase in appendicular skeletal muscle mass, which is compatible with but does not establish skeletal muscle hypertrophy. Body-composition-derived estimates of muscle or lean mass may also be influenced by water, glycogen, and non-contractile components, whereas changes in muscle thickness or cross-sectional area assessed by ultrasound or MRI provide more direct morphological evidence of hypertrophy [58]. At the same time, the present NMA evaluated whey protein + vitamin D + leucine as a combined formulation; therefore, the observed effects characterize the efficacy of the formulation as a whole and do not establish whether interactions among its individual components are additive, complementary, or synergistic. Although this formulation was supported by multiple trials, the trial-level leave-one-out analyses showed that the ASMI and grip-strength estimates were influenced by individual contributing studies. This variation suggests that the observed effects may depend partly on differences in study populations or intervention contexts rather than representing a uniform response to the formulation across trials.

Omega-3 fatty acids + leucine + Lactobacillus paracasei PS23 also showed statistically significant effects on ASMI and grip strength. The composition of this formulation raises the possibility that several pathways relevant to muscle health may be targeted concurrently, although the present analysis cannot determine the contribution of each component or their interactions. Previous evidence suggests that omega-3 fatty acids may influence muscle health in older adults by modulating inflammatory responses and the sensitivity of muscle protein synthesis [10], while microbiota-targeted interventions have also attracted increasing attention in relation to sarcopenia [59]. However, this formulation was evaluated in only one RCT, and its observed effects therefore cannot yet be separated from the characteristics of that individual trial. Independent replication and direct head-to-head comparisons are needed to determine whether these effects are reproducible and attributable to the formulation across different populations and treatment settings.

This study also found that nutritional response patterns were not fully concordant across muscle mass, muscle strength, and physical performance. This was particularly evident for physical performance: pairwise meta-analysis showed a statistically significant overall effect on gait speed but not on 5STS, whereas the NMA identified formulation-specific effects of leucine + vitamin D + medium-chain triglycerides on gait speed and whey protein + vitamin D + leucine on 5STS. These findings reflect the different questions addressed by the two analytical approaches. Pairwise meta-analysis estimated the average effect of nutritional supplementation across different formulations, whereas the NMA treated specific formulations as distinct intervention nodes and estimated their relative effects by integrating direct and indirect evidence [13,16,17]. Accordingly, a non-significant pooled effect across nutritional interventions does not exclude an effect associated with a specific formulation, while a significant pooled effect does not imply that all individual formulations are similarly effective. Differences across outcomes may also reflect the distinct physiological dimensions captured by muscle mass, muscle strength, and physical-performance measures. Muscle mass and grip strength more directly reflect body composition and force-generating capacity. In contrast, gait speed and sit-to-stand performance are influenced by multiple factors, including balance, neuromuscular coordination, joint function, cardiorespiratory capacity, pain, cognition, and habitual activity levels [5,60]. Thus, improvements in muscle mass or strength may not translate directly into parallel changes across all measures of physical performance. The 5STS is relatively dependent on lower-limb strength and repeated transfer ability, whereas gait speed reflects broader mobility and may be influenced by additional physiological and testing-related factors [5,60]. At the same time, the gait-speed effect for leucine + vitamin D + medium-chain triglycerides was derived from a single-study node, whereas the 5STS effect for whey protein + vitamin D + leucine arose from a formulation represented across multiple trials. These findings also indicate that no single outcome should be used as a surrogate for the overall effect of nutritional intervention in sarcopenia. Muscle mass, muscle strength, and different dimensions of physical performance should therefore be considered together when evaluating treatment effects.

Pairwise meta-analyses showed substantial heterogeneity for grip strength (*I*^2^ = 85%) and 5STS (*I*^2^ = 91%) and moderate heterogeneity for gait speed (*I*^2^ = 65%). Subgroup analyses did not consistently explain these differences, and meta-regression found no significant linear association between intervention duration and the effects on grip strength or gait speed. Thus, intervention duration alone does not appear to account for the observed between-study variability. The influence analysis provided further evidence that heterogeneity was not evenly distributed across studies. In particular, exclusion of Rondanelli et al. (2020) [52] reduced *I*^2^ from 65% to 0% for gait speed and from 91% to 51% for 5STS, indicating that this study contributed substantially to the between-study variability in physical-performance outcomes. The marked reduction in heterogeneity after exclusion of a single comparison suggests that differences in study-level characteristics, rather than intervention duration alone, may be important in explaining variation in physical-performance responses. Across the included trials, potential sources of such variability included differences in sarcopenia definitions and baseline risk status, baseline nutritional status, supplement composition and dose, adherence, concurrent exercise or rehabilitation programs, and outcome-assessment procedures [6,23,24,61]. A similar pattern was evident in the trial-level NMA influence analyses: the estimated effects of whey protein + vitamin D + leucine on ASMI and grip strength changed after exclusion of individual contributing trials, whereas the 5STS effect remained statistically significant across these exclusions although its magnitude varied. Taken together, these findings suggest that between-study variability may influence both the magnitude and precision of formulation-specific effects and that treatment responses may depend partly on population and intervention context. At the same time, global and local inconsistency tests were not statistically significant, but the networks contained many sparsely represented nodes and few closed loops. Therefore, non-significant inconsistency tests do not exclude clinically important differences among the studies contributing to the network. Network coherence should therefore be considered alongside the distribution of potential effect modifiers, trial-level influence, network structure, and certainty of evidence when interpreting formulation-specific estimates.

Population definition appears to be one relevant source of clinical variability. The primary analysis included both participants with confirmed sarcopenia and those considered at high risk to capture the available randomized evidence across the broader spectrum of sarcopenia-related impairment; however, these populations do not necessarily represent the same clinical state. Participants with confirmed sarcopenia may differ from high-risk populations in the degree of muscle impairment, functional reserve, and underlying nutritional deficits, which may influence their response to nutritional supplementation [6,21,22,23]. In the analysis restricted to participants with confirmed sarcopenia, the ASMI effects of whey protein + vitamin D + leucine and omega-3 fatty acids + leucine + Lactobacillus paracasei PS23 were retained, whereas the grip-strength and 5STS effects of whey protein + vitamin D + leucine were no longer statistically significant. For gait speed, the leucine + vitamin D + medium-chain triglyceride node was no longer represented in the restricted network. These changes suggest that population definition can affect both the composition of the evidence network and the precision of formulation-specific estimates. Importantly, loss of statistical significance in the restricted analysis does not necessarily indicate absence of an intervention effect, because restricting the population reduced the available evidence and increased uncertainty for several comparisons. Variation in diagnostic criteria may further contribute to clinical heterogeneity by identifying populations with different combinations and severities of low muscle mass, reduced muscle strength, and impaired physical performance [21,22,23]. Accordingly, the primary findings describe nutritional effects across the broader spectrum of sarcopenia-related impairment, whereas their applicability specifically to clinically diagnosed sarcopenia remains less certain for some outcomes.

The use of SMDs also affects the clinical interpretation of the observed effects. SMDs enabled results obtained using different measurement methods and scales to be synthesized on a common metric, but they do not directly indicate the absolute magnitude of improvement in clinically familiar units [17]. Consequently, a statistically significant SMD should not be interpreted automatically as a clinically meaningful improvement [17]. The present findings therefore provide evidence about the direction and relative magnitude of effects across nutritional strategies, whereas the extent to which these effects translate into clinically perceptible changes in muscle mass, strength, or physical performance remains uncertain. Future trials should report changes in original units alongside standardized effects and, where established, clinically meaningful thresholds to improve the clinical interpretability of pooled estimates.

From a clinical perspective, the findings suggest that the effects of nutritional supplementation may depend on both formulation composition and patient context rather than supporting a single nutritional strategy for all older adults with sarcopenia-related impairment. Whey protein + vitamin D + leucine represents a candidate multi-nutrient strategy because favorable effects were observed across several outcomes and the formulation was represented in multiple trials, but the influence analyses indicate that the magnitude and precision of these effects varied according to the contributing evidence. The findings for omega-3 fatty acids + leucine + Lactobacillus paracasei PS23 and leucine + vitamin D + medium-chain triglycerides identify additional formulations of interest, but their current evidence base is limited to single-study nodes. In practice, the relevance of a given formulation is also likely to depend on the nutritional and functional characteristics of the individual. Baseline protein intake, vitamin D status, and the presence of nutritional deficiency or risk may influence the potential for supplementation to address an existing nutritional gap, while adherence determines the extent to which the intended intervention is actually received [6,7,23,24]. Concurrent resistance exercise or rehabilitation should also be considered because these co-interventions can independently affect muscle strength and physical performance and may modify the context in which nutritional effects are observed [61]. Comorbidities and functional status may further influence both nutritional needs and the feasib ility of a given intervention [7,23]. Accordingly, the present findings are better used to identify candidate nutritional strategies for individualized evaluation according to nutritional deficits, clinical characteristics, concurrent exercise or rehabilitation, and adherence than to establish a universal treatment hierarchy.

## 5. Limitations and Future Directions

This study has several limitations. First, although 20 RCTs were included, evaluating interventions at the level of specific formulations created numerous network nodes, some supported by only one study. The limited number of direct head-to-head comparisons and closed loops reduced the precision of some network estimates and limited the extent to which formulation-specific effects could be independently replicated within the network. Second, the included populations comprised both participants with confirmed sarcopenia and those at high risk of sarcopenia, and the studies differed in diagnostic criteria, baseline nutritional status, intervention dose, administration frequency, adherence, and concurrent exercise or rehabilitation programs. These differences may have contributed to residual clinical het-erogeneity. Third, to maintain network connectivity, placebo, usual diet, and no additional nutritional supplementation were combined into a common Control node. Although background conditions were generally comparable across randomized groups within individual studies, variation in the form of the control condition across studies may still have introduced heterogeneity. Fourth, some studies did not report the standard deviation of change scores. A prespecified correlation coefficient of r = 0.5 was therefore used for estimation, introducing additional uncertainty. In addition, because measurement devices and assessment protocols differed across studies, SMDs were used for pooling. Although this facilitated comparison across studies, it limited the direct interpretation of effect sizes in clinically familiar units. Fifth, comparison-adjusted funnel plots for grip strength and gait speed showed some asymmetry, whereas the numbers of studies for ASMI and 5STS were insufficient to assess small-study effects reliably. The certainty of evidence for the principal comparisons was also very low, limiting confidence in the precision and reproducibility of the observed comparative effects. Sixth, sex-specific differences in the effects of nutritional interventions were not evaluated. In addition, sample sizes and male-to-female ratios varied across the included RCTs, which may limit the generalizability of the findings. Finally, the full texts of five potentially eligible studies could not be obtained through available institutional and library resources, and the original authors were not contacted. Consequently, these studies could not be assessed for eligibility, which may have affected the completeness of the evidence and potentially influenced the pooled estimates.

Future research should prioritize adequately powered, preregistered RCTs that directly compare potentially advantageous formulations. Such trials should harmonize sarcopenia diagnostic criteria, reporting of nutrient composition and dose, adherence assessment, and measurement of core outcomes such as ASMI, grip strength, gait speed, and 5STS [21,22,23]. When muscle hypertrophy is an intended outcome, direct morphological measures such as muscle thickness or cross-sectional area assessed by ultrasound or MRI should be considered alongside body-composition measures such as ASMI [55]. For multi-nutrient strategies, authors should further clarify key components and dose–response relationships while ensuring the overall formulation can be replicated. Background diet and concurrent exercise or rehabilitation programs should also be fully reported to distinguish effects attributable to nutritional supplementation from those related to co-interventions and to determine whether treatment effects vary across different background conditions. Longer-term follow-up should additionally evaluate patient-important outcomes such as falls, disability, quality of life, and hospitalization. As high-quality head-to-head evidence accumulates, future NMAs could conduct stratified comparisons by baseline nutritional status, sarcopenia severity, and concurrent exercise or rehabilitation, improving the personalization and clinical translatability of nutritional intervention selection. To further strengthen the evidence base, future updates of this systematic review should also make greater efforts to obtain potentially eligible studies, including contacting original study authors when full texts are unavailable through conventional retrieval channels.

## 6. Conclusions

This systematic review and network meta-analysis indicates that nutritional supplementation overall improves ASMI, grip strength, and gait speed in older adults with sarcopenia or at high risk of sarcopenia, but the effects of specific nutritional formulations are outcome-dependent. Whey protein + vitamin D + leucine showed statistically significant effects on ASMI, grip strength, and 5STS, although the magnitude and precision of some estimates varied in trial-level and population-restricted sensitivity analyses. Omega-3 fatty acids + leucine + Lactobacillus paracasei PS23 showed statistically significant effects on ASMI and grip strength, and leucine + vitamin D + medium-chain triglycerides showed a statistically significant effect on gait speed; however, both formulations were supported by single-study nodes. Given the sparse network and limited certainty of the current evidence for the principal comparisons, these findings should be regarded as formulation-specific candidate signals rather than evidence of a definitive treatment hierarchy. Further adequately powered head-to-head RCTs are needed to determine their reproducibility, clinically meaningful effects, and applicability across different nutritional and rehabilitation contexts.

## Figures and Tables

**Table 1:** Characteristics of the included studies.

Author	Year	Country/ Region	Sample (*n*)	Gender (Male/Female)	Age	Diagnostic Criteria	Sarcopenia Status	Intervention Duration	Intervention	Outcomes
Flakoll [44]	2004	USA	27	IG (total): 14 CG (total): 13	IG: 71.50 ± 1.5 CG: 71.50 ± 1.5	Low muscle mass + low strength + slow gait	At risk	12 weeks	HMB+ARG+LYS	②
Alemán-Mateo [39]	2012	Mexico	40	IG: 8/12 CG: 9/11	IG: 75.40 ± 5.0 CG: 76.70 ± 5.8	Residuals Method (DXA)	Confirmed	12 weeks	P	②
Bauer [34]	2015	Germany	380	IG: 64/120 CG: 67/129	IG: 77.30 ± 6.7 CG: 78.10 ± 7.0	EWGSOP	Confirmed	13 weeks	WP+VD+Leu	②③④
Abe [38]	2016	Japan	36	LD+MCT: 4/9 LD+LCT: 3/9 CG: 2/9	LD+MCT: 85.50 ± 3.7 LD+LCT: 87.70 ± 3.9 CG: 86.80 ± 6.5	Low muscle mass + (low strength/slow gait)	At risk	12 weeks	Leu+VD+MCT Leu+VD+LCT	②③
Rondanelli [53]	2016	Italy	130	IG: 29/40 CG: 24/37	IG: 80.77 ± 6.29 CG: 80.21 ± 8.54	EWGSOP	Confirmed	12 weeks	WP+VD+Leu	①②
Cramer (sarcopenia subgroup) [50]	2016	USA	144	IG (total): 80 CG (total): 64	IG: 77 (71, 81) CG: 77 (71, 81)	EWGSOP	Confirmed	24 weeks	WP+CaHMB	③
Cramer (at-risk sarcopenia subgroup) [50]	2016	USA	184	IG (total):83 CG (total):101	IG: 77 (71, 81) CG: 77 (71, 81)	EWGSOP	At risk	24 weeks	WP+CaHMB	③
Ottestad [46]	2017	Norway	36	IG: 5/12 CG: 7/12	IG: 76.80 ± 6.2 CG: 77.10 ± 4.7	Low strength/slow gait/impaired functional performance	At risk	12 weeks	P	②④
El Hajj [41]	2018	Lebanon	115	IG: 33/27 CG: 26/29	IG: 73.05 ± 1.95 CG: 73.56 ± 2.14	Low muscle mass + Vitamin D deficiency	At risk	24 weeks	VD	②
Park [47]	2018	South Korea	120	WP: 12/28 P: 14/26 CG: 16/24	WP: 76.80 ± 3.70 P: 77.30 ± 3.67 CG: 76.83 ± 3.86	Low muscle mass + low strength+ impaired functional performance	At risk	12 weeks	WP P	③
Bo [40]	2019	China	60	IG: 13/17 CG: 14/16	IG: 73.23 ± 6.52 CG: 74.83 ± 5.94	Low muscle mass + (low strength/slow gait)	Confirmed	24 weeks	WP+VD+VE	①②③④
Björkman [35]	2020	Finland	218	WP: 22/51 P: 27/46 CG: 21/51	WP: 83.60 ± 4.7 P: 84.0 ± 3.9 CG: 83.70 ± 5.1	EWGSOP	Confirmed	48 weeks	WP P	②
Rondanelli [52]	2020	Italy	127	IG: 26/38 CG: 17/46	IG: 81 ± 7 CG: 82 ± 5	EWGSOP	Confirmed	6 weeks	WP+VD+Leu	①②③④
Martínez-Arnau [43]	2020	Spain	43	IG (total): 21 CG (total): 22	IG: 78.70 ± 21.2 CG: 78.0 ± 21.6	EWGSOP	Confirmed	13 weeks	Leu	①②
Lin [37]	2021	Taiwan (China)	56	IG: 21/7 CG: 19/9	IG: 72.50 ± 5.57 CG: 73.80 ± 8.11	AWGS	Confirmed	12 weeks	WP+VD+Leu P	①②③
Murphy [51]	2021	Ireland	107	LEU-PRO+*n*-3: 17/21 LEU-PRO: 18/20 CG: 17/14	LEU-PRO+*n*-3: 73 ± 6 LEU-PRO: 70 ± 5 CG: 73 ± 7	Low muscle mass (+ low grip strength)	At risk	24 weeks	WP+Leu+LCT WP+Leu	②③④
Osuka [45]	2021	Japan	75	IG (total): 37 CG (total): 38	IG: 71.50 ± 4.5 CG: 71.60 ± 4.2	AWGS	Confirmed	12 weeks	Ca+HMB	①②③
Rondanelli [49]	2022	Italy	50	IG (total): 22 CG (total): 28	IG: 78.84 ± 5.80 CG: 80.50 ± 3.74	EWGSOP	Confirmed	8 weeks	ω-3+Leu+PS23	①②
Chen [36]	2024	China	68	OG (total): 25 GG (total): 22 CG (total): 21	OG: 71 (67, 73) GG: 75 (70, 77) CG: 73 (67, 76)	AWGS	At risk	12 weeks	P+Ca P+Ca+CaP	②③④
Kao [48]	2025	Taiwan (China)	97	IG: 12/36 CG: 12/37	IG: 72.60 ± 5.1 CG: 72.30 ± 5.2	SARC-F ≥ 4 (SARC-CalF ≥ 11)	At risk	12 weeks	P	①②④
Chung [42]	2026	Malaysia	47	IG: 3/22 CG: 2/20	IG: 68.80 ± 6.5 CG: 69.70 ± 7.9	AWGS	Confirmed	12 weeks	WP+Leu	①④

Notes: WP+VD+VE, whey protein + vitamin D + vitamin E; ω-3+Leu+PS23, ω-3 fatty acids + leucine + Lactobacillus paracasei PS23; WP+VD+Leu, whey protein + vitamin D + leucine; WP, whey protein; Leu, leucine; VD, vitamin D; P+Ca, protein + calcium; P+Ca+CaP, protein + calcium + calcium-containing phosphates; WP+Leu, whey protein + leucine; WP+Leu+LCT, whey protein + leucine + long-chain lipid formulation containing *n*-3 PUFA; Leu+VD+MCT, leucine + vitamin D + medium-chain triglycerides; Leu+VD+LCT, leucine + vitamin D + long-chain triglycerides; HMB+ARG+LYS, β-hydroxy-β-methylbutyrate + arginine + lysine; Ca+HMB, calcium + β-hydroxy-β-methylbutyrate; P, protein; WP+CaHMB, whey protein + calcium β-hydroxy-β-methylbutyrate. ①, appendicular skeletal muscle mass index (ASMI); ②, grip strength; ③, gait speed; ④, five-times sit-to-stand test (5STS). IG, intervention group; CG, control group; OG, original cheese group; GG, golden cheese group. EWGSOP, European Working Group on Sarcopenia in Older People; AWGS, Asian Working Group for Sarcopenia. SARC-F, Sarcopenia risk screening tool (score 0–10; ≥4 indicates risk); SARC-CalF, SARC-F combined with calf circumference measurement (score 0–20; ≥11 indicates risk; calf circumference threshold: <34 cm for males, <33 cm for females). DXA, dual-energy X-ray absorptiometry. Cramer (sarcopenia subgroup) and Cramer (at-risk sarcopenia subgroup) represent two eligible subgroups from the same study and were retained separately for data extraction and analysis.

## Data Availability

The original contributions presented in this study are included in the article/Supplementary Materials. Further inquiries can be directed to the corresponding authors.

## Supplementary Materials

The following supporting information can be downloaded at: https://www.mdpi.com/article/10.3390/nu18203368/s1, Table S1. Potential effect modifiers considered in the assessment of transitivity; Table S2. Intervention node classification for network meta-analysis; Table S3. Distribution of potential effect modifiers across indirect comparisons for appendicular skeletal muscle mass index (ASMI); Table S4. Distribution of potential effect modifiers across indirect comparisons for grip strength; Table S5. Distribution of potential effect modifiers across indirect comparisons for gait speed; Table S6. Distribution of potential effect modifiers across indirect comparisons for the five-times sit-to-stand test (5STS); Table S7. Meta-regression analysis of the effect of intervention duration on grip strength (Pairwise meta-analysis); Table S8. Meta regression analysis of the effect of intervention duration on gait speed (Pairwise meta analysis); Table S9. Leave-one-out sensitivity analysis of grip strength (pairwise meta-analysis); Table S10. Leave-one-out sensitivity analysis of gait speed (pairwise meta-analysis); Table S11. Leave-one-out sensitivity analysis of 5STS (pairwise meta-analysis); Table S12. Comparison of pooled effect estimates between the primary analysis and the broader-node sensitivity analysis (pairwise meta-analysis); Table S13. Population-restriction sensitivity analysis of pairwise meta-analysis restricted to participants with confirmed sarcopenia (pairwise meta-analysis); Table S14. Sensitivity analysis excluding single-study intervention nodes for appendicular skeletal muscle mass index (ASMI); Table S15. Sensitivity analysis excluding single-study intervention nodes for grip strength; Table S16. Sensitivity analysis excluding single-study intervention nodes for gait speed; Table S17. Sensitivity analysis excluding single-study intervention nodes for five-times sit-to-stand test (5STS); Table S18. Trial-level leave-one-out network meta-analysis for appendicular skeletal muscle mass index (ASMI); Table S19. Trial-level leave-one-out network meta-analysis for grip strength; Table S20. Trial-level leave-one-out network meta-analysis for gait speed; Table S21. Trial-level leave-one-out network meta-analysis for five-times sit-to-stand test (5STS); Table S22. Alternative classification of intervention nodes used in the sensitivity analysis; Table S23. Network meta-analysis results using the alternative node classification; Table S24. Population-restriction sensitivity analysis of the network meta-analysis for ASMI; Table S25. Population-restriction sensitivity analysis of the network meta-analysis for grip strength; Table S26. Population-restriction sensitivity analysis of the network meta-analysis for gait speed; Table S27. Population-restriction sensitivity analysis of the network meta-analysis for 5STS; Table S28. Summary of findings and certainty of evidence for principal intervention–outcome comparisons; Table S29. Certainty of evidence for each pairwise comparison of nutritional strategies on appendicular skeletal muscle mass index (ASMI) in older adults using the CINeMA framework; Table S30. Certainty of evidence for each pairwise comparison of nutritional strategies on grip strength in older adults using the CINeMA framework; Table S31. Certainty of evidence for each pairwise comparison of nutritional strategies on gait speed in older adults using the CINeMA framework; Table S32. Certainty of evidence for each pairwise comparison of nutritional strategies on five-times sit-to-stand test (5STS) in older adults using the CINeMA framework; Figure S1. Forest plot of pairwise meta-analysis for appendicular skeletal muscle mass index (ASMI); Figure S2. Forest plot of pairwise meta-analysis for grip strength, with subgroup analyses; Figure S3. Forest plot of pairwise meta-analysis for gait speed, with subgroup analyses; Figure S4. Forest plot of pairwise meta-analysis for five-times sit-to-stand test (5STS), with subgroup analyses; Figure S5. League table of network meta-analysis for appendicular skeletal muscle mass index (ASMI); Figure S6. League table of network meta-analysis for grip strength; Figure S7. League table of network meta-analysis for gait speed; Figure S8. League table of network meta-analysis for five-times sit-to-stand test (5STS); Figure S9. Network plots based on the alternative node classification.

## Abbreviations

The following abbreviations are used in this manuscript:

ASMI	Appendicular Skeletal Muscle Mass Index
5STS	Five-Times Sit-To-Stand Test
AWGS	Asian Working Group for Sarcopenia
EWGSOP	European Working Group on Sarcopenia
IG	Intervention Group
CG	Control Group
OG	Original Cheese Group
GG	Golden Cheese Group
SARC-F	Sarcopenia Risk Screening Tool
DXA	Dual-energy X-ray Absorptiometry
WP+VD+VE	Whey Protein + Vitamin D + Vitamin E
ω-3+Leu+PS23	ω-3 fatty acids + Leucine + Lactobacillus paracasei PS23
WP+VD+Leu	Whey Protein + Vitamin D + Leucine
WP	Whey Protein
Leu	Leucine
VD	Vitamin D
P+Ca	Protein + Calcium
P+Ca+CaP	Protein + Calcium + Calcium-containing Phosphates
WP+Leu	Whey Protein + Leucine
WP+Leu+LCT	whey protein + leucine + long-chain lipid formulation containing *n*-3 PUFA
Leu+VD+MCT	Leucine + Vitamin D + Medium-chain Triglycerides
Leu+VD+LCT	Leucine + Vitamin D + Long-chain Triglycerides
HMB+ARG+LYS	β-hydroxy-β-methylbutyrate + Arginine + Lysine
Ca+HMB	Calcium + β-hydroxy-β-methylbutyrate
P	Protein
WP+CaHMB	Whey Protein + Calcium β-hydroxy-β-methylbutyrate
