# Supplementary material for: Comparative Effectiveness of Nutritional Strategies for Sarcopenia-Related Outcomes in Older Adults: A Systematic Review and Network Meta-Analysis
Source: Nutrients. 2026 Oct 14;18(20):3368. doi: 10.3390/nu18203368 (PMC13708331; doi:10.3390/nu18203368)
Supplement: Supplementary file 1 [file nutrients-18-03368-s001.zip › nutrients-4592777-supplementary.pdf]

Supplementary Material

Comparative effectiveness of nutritional strategies for sarcopenia-related outcomes in older adults: a systematic review and network meta-analysis

Contents

Supplementary Table S1. Potential effect modifiers considered in the assessment of transitivity 1
Supplementary Table S2. Intervention node classification for network meta-analysis........5
Supplementary Table S3. Distribution of potential effect modifiers across indirect comparisons for appendicular skeletal muscle mass index (ASMI) 7
Supplementary Table S4. Distribution of potential effect modifiers across indirect comparisons for grip strength 19
Supplementary Table S5. Distribution of potential effect modifiers across indirect comparisons for gait speed 58
Supplementary Table S6. Distribution of potential effect modifiers across indirect comparisons for the five-times sit-to-stand test (5STS)..87
Supplementary Table S7. Meta-regression analysis of the effect of intervention duration on grip strength (Pairwise meta-analysis)..98
Supplementary Table S8. Meta-regression analysis of the effect of intervention duration on gait speed (Pairwise meta-analysis)..98
Supplementary Table S9. Leave-one-out sensitivity analysis of grip strength (pairwise meta-analysis)..99
Supplementary Table S10. Leave-one-out sensitivity analysis of gait speed (pairwise meta-analysis)..100
Supplementary Table S11. Leave-one-out sensitivity analysis of 5STS (pairwise meta-analysis)..101
Supplementary Table S12. Comparison of pooled effect estimates between the primary analysis and the broader-node sensitivity analysis (pairwise meta-analysis)..................101
Supplementary Table S13. Population-restriction sensitivity analysis of pairwise meta-analysis restricted to participants with confirmed sarcopenia (pairwise meta-analysis) 101
Supplementary Table S14. Sensitivity analysis excluding single-study intervention nodes for appendicular skeletal muscle mass index (ASMI)..103
Supplementary Table S15. Sensitivity analysis excluding single-study intervention nodes for grip strength 103
Supplementary Table S16. Sensitivity analysis excluding single-study intervention nodes for gait speed 103
Supplementary Table S17. Sensitivity analysis excluding single-study intervention nodes for five-times sit-to-stand test (5STS)..103
Supplementary Table S18. Trial-level leave-one-out network meta-analysis for appendicular skeletal muscle mass index (ASMI)..104
Supplementary Table S19. Trial-level leave-one-out network meta-analysis for grip strength..104

Supplementary Table S20. Trial-level leave-one-out network meta-analysis for gait speed 104
Supplementary Table S21. Trial-level leave-one-out network meta-analysis for five-times sit-to-stand test (5STS) 105
Supplementary Table S22. Alternative classification of intervention nodes used in the sensitivity analysis 106
Supplementary Table S23. Network meta-analysis results using the alternative node classification 106
Supplementary Table S24. Population-restriction sensitivity analysis of the network meta-analysis for ASMI 107
Supplementary Table S25. Population-restriction sensitivity analysis of the network meta-analysis for grip strength 107
Supplementary Table S26. Population-restriction sensitivity analysis of the network meta-analysis for gait speed 108
Supplementary Table S27. Population-restriction sensitivity analysis of the network meta-analysis for 5STS 108
Supplementary Table S28. Summary of findings and certainty of evidence for principal intervention–outcome comparisons 109
Supplementary Table S29. Certainty of evidence for each pairwise comparison of nutritional strategies on appendicular skeletal muscle mass index (ASMI) in older adults using the CINeMA framework 110
Supplementary Table S30. Certainty of evidence for each pairwise comparison of nutritional strategies on grip strength in older adults using the CINeMA framework ...113
Supplementary Table S31. Certainty of evidence for each pairwise comparison of nutritional strategies on gait speed in older adults using the CINeMA framework 125
Supplementary Table S32. Certainty of evidence for each pairwise comparison of nutritional strategies on five-times sit-to-stand test (5STS) in older adults using the CINeMA framework 133
Supplementary Figure S1. Forest plot of pairwise meta-analysis for appendicular skeletal muscle mass index (ASMI) 136
Supplementary Figure S2. Forest plot of pairwise meta-analysis for grip strength, with subgroup analyses 137
Supplementary Figure S3. Forest plot of pairwise meta-analysis for gait speed, with subgroup analyses 138
Supplementary Figure S4. Forest plot of pairwise meta-analysis for five-times sit-to-stand test (5STS), with subgroup analyses 139
Supplementary Figure S5. League table of network meta-analysis for appendicular skeletal muscle mass index (ASMI) 140
Supplementary Figure S6. League table of network meta-analysis for grip strength 140
Supplementary Figure S7. League table of network meta-analysis for gait speed 141
Supplementary Figure S8. League table of network meta-analysis for five-times sit-to-stand test (5STS) 141
Supplementary Figure S9. Network plots based on the alternative node classification ..142

Supplementary Table S1. Potential effect modifiers considered in the assessment of transitivity

Study	Intervention node(s)	Age, years	Gender (Male/Female)	Sarcopenia status	Diagnostic criteria	Intervention duration	Co-intervention	Dose	Adherence
Flakoll, 2004	HMB+ARG+LYS	IG: 71.50±1.5<br>CG: 71.50±1.5	IG (total): 14<br>CG (total): 13	At risk	Low muscle mass + low strength+ slow gait	12 weeks	No	2 g HMB, 5g arginine, 1.5g lysine per day	High
Alemán-Mateo, 2012	P	IG: 75.40±5.0<br>CG: 76.70±5.8	IG: 8/12<br>CG: 9/11	Confirmed	Residuals Method (DXA)	12 weeks	No	15.7g protein per day, 7 times a week	NR
Bauer, 2015	WP+VD+Leu	IG: 77.30±6.7<br>CG: 78.10±7.0	IG: 64/120<br>CG: 67/129	Confirmed	EWGSOP	13 weeks	No	20g whey protein, 800 IU vitamin D, 3g leucine, twice daily	High
Abe, 2016	Leu+VD+MCT<br>Leu+VD+LCT	LD+MCT: 85.50±3.7<br>LD+LCT: 87.70±3.9<br>CG: 86.80±6.5	LD+MCT: 4/9<br>LD+LCT: 3/9<br>CG: 2/9	At risk	Low muscle mass + (low strength / slow gait)	12 weeks	Regular Exercise Activities in Nursing Homes	1.2g leucine, 800IU vitamin D, 6g MCT or LCT per day	High
Rondanelli, 2016	WP+VD+Leu	IG: 80.77±6.29<br>CG: 80.21±8.54	IG: 29/40<br>CG: 24/37	Confirmed	EWGSOP	12 weeks	Structured training 5 times a week, 20 minutes each session	22g whey protein, 100IU vitamin D, 4g leucine per day	High
Cramer (sarcopenia subgroup), 2016	WP+CaHMB	IG: 77(71,81)<br>CG: 77(71,81)	IG (total): 80<br>CG (total): 64	Confirmed	EWGSOP	24 weeks	No	660 kcal, 40g protein, 3.0g CaHMB per day	High
Cramer (at-risk sarcopenia subgroup), 2016	WP+CaHMB	IG: 77(71,81)<br>CG: 77(71,81)	IG (total): 83<br>CG (total): 101	At risk	EWGSOP	24 weeks	No	660 kcal, 40g protein, 3.0g CaHMB per day	High

Study	Interventio n node(s)	Age, years	Gender (Male/Femal e)	Sarcopenia status	Diagnostic criteria	Intervention duration	Co-intervention	Dose	Adhere nce
Ottestad , 2017	P	IG: 76.80±6.2 CG: 77.10±4.7	IG: 5/12 CG: 7/12	At risk	Low muscle mass + (low strength / slow gait)	12 weeks	No	20g protein, twice daily	High
El Hajj, 2018	VD	IG: 73.05±1.95 CG: 73.56±2.14	IG: 33/27 CG: 26/29	At risk	Low muscle mass + Vitamin D deficiency	24 weeks	No	10,000IU vitamin D, 3 times a week	High
Park, 2018	WP P	WP: 76.80±3.70 P: 77.30±3.67 CG: 76.83±3.86	WP: 12/28 P:14/26 CG: 16/24	At risk	Low muscle mass + low strength+ impaired functional performance	12 weeks	No	20g Whey protein; 14g protein per day	High
Bo, 2019	WP+VD+V E	IG: 73.23±6.52 CG: 74.83±5.94	IG: 13/17 CG: 14/16	Confirmed	Low muscle mass + (low strength / slow gait)	24 weeks	No	22g whey protein, 702IU vitamin D, 109mg vitamin E, twice daily	NR
Björkma n, 2020	WP P	WP: 83.60±4.7 P: 84.0±3.9 CG: 83.70±5.1	WP: 22/51 P: 27/46 CG: 21/51	Confirmed	EWGSOP	48 weeks	Low-Intensity Home Workouts	20g Whey protein; 3.75g protein, twice daily	Low
Rondan elli, 2020	WP+VD+ Leu	IG: 81±7 CG: 82±5	IG: 26/38 CG: 17/46	Confirmed	EWGSOP	6 weeks	Personalized physical rehabilitation therapy 5 times a week, 20–30 minutes per session	20g whey protein, 800IU vitamin D, 2.8g leucine, twice daily	High
Martíne z-Arnau, 2020	Leu	IG: 78.70±21.2 CG: 78.0±21.6	IG (total): 21 CG (total): 22	Confirmed	EWGSOP	13 weeks	No	3g leucine, twice daily	High
Lin, 2021	WP+VD+Le u	IG: 72.50±5.57 CG: 73.80±8.11	IG: 21/7 CG: 19/9	Confirmed	AWGS	12 weeks	No	8.5g whey protein, 120IU vitamin D,	High

Study	Intervention node(s)	Age, years	Gender (Male/Female)	Sarcopenia status	Diagnostic criteria	Intervention duration	Co-intervention	Dose	Adherence
Murphy, 2021	WP+Leu+LCT<br>WP+Leu	LEU-PRO+n-3: 73±6<br>LEU-PRO: 70±5<br>CG: 73±7	LEU-PRO+n-3: 17/21<br>LEU-PRO: 18/20<br>CG: 17/14	At risk	Low muscle mass (+ low grip strength)	24 weeks	No	1.2g leucine per day 10.6g whey protein, 3.1 g leucine, twice daily; with or without 2.4g n-3 PUFA	High
Osuka, 2021	Ca+HMB	IG: 71.50±4.5<br>CG: 71.60±4.2	IG (total): 37<br>CG (total): 38	Confirmed	AWGS	12 weeks	No	207mg calcium, 1200mg HMB per day	High
Rondanelli, 2022	ω-3+Leu +PS23	IG: 78.84±5.80<br>CG: 80.50±3.74	IG (total): 22<br>CG (total): 28	Confirmed	EWGSOP	8 weeks	Basic exercise 5 times a week for 20 to 30 minutes	2.5g omega-3, 500mg leucine, 3.0 × 10^9CFU PS23, per day	High
Chen, 2024	P+Ca<br>P+Ca+CaP	OG: 71(67,73)<br>GG: 75(70,77)<br>CG: 73(67,76)	OG (total): 25<br>GG (total): 22<br>CG (total): 21	At risk	AWGS	12 weeks	All were instructed to maintain their current level of physical activity	9.0 g protein + 322.8 mg calcium per day; 12.7 g protein + 802.1 mg calcium + additional calcium-containing phosphates (CaP) per day	High
Kao, 2025	P	IG: 72.60±5.1<br>CG: 72.30±5.2	IG: 12/36<br>CG: 12/37	At risk	SARC-F≥4 (SARC-CalF≥11)	12 weeks	No	24–30g protein per day	High
Chung, 2026	WP+Leu	IG: 68.80±6.5<br>CG: 69.70±7.9	IG: 3/22<br>CG: 2/20	Confirmed	AWGS	12 weeks	No	25.3g whey protein, 3g leucine, twice daily	Low

Notes: WP+VD+VE, whey protein + vitamin D + vitamin E; ω-3+Leu+PS23, ω-3 fatty acids + leucine + Lactobacillus paracasei PS23; WP+VD+Leu, whey protein + vitamin D + leucine; WP, whey protein; Leu, leucine; VD, vitamin D; P+Ca, protein + calcium; P+Ca+CaP, protein + calcium + calcium-containing phosphates; WP+Leu, whey protein + leucine; WP+Leu+LCT, whey protein + leucine + long-chain lipid formulation containing n-3 PUFA; Leu+VD+MCT, leucine + vitamin D + medium-chain triglycerides; Leu+VD+LCT, leucine + vitamin D + long-chain triglycerides; HMB+ARG+LYS, β-hydroxy-β-methylbutyrate + arginine + lysine; Ca+HMB, calcium + β-hydroxy-β-methylbutyrate; P, protein; WP+CaHMB, whey protein + calcium β-hydroxy-β-methylbutyrate. IG, intervention group; CG, control group; OG, original cheese group; GG, golden cheese group. NR, not

reported; High adherence, high adherence to the prescribed nutritional intervention; Low adherence, low adherence to the prescribed nutritional intervention; High adherence: >70%; Low adherence: <70%. EWGSOP, European Working Group on Sarcopenia in Older People; AWGS, Asian Working Group for Sarcopenia. SARC-F, Sarcopenia risk screening tool (score 0–10; ≥4 indicates risk); SARC-CalF, SARC-F combined with calf circumference measurement (score 0–20; ≥11 indicates risk; calf circumference threshold: <34 cm for males, <33 cm for females). DXA, dual-energy X-ray absorptiometry. Cramer (sarcopenia subgroup) and Cramer (at-risk sarcopenia subgroup) represent two eligible subgroups from the same study and were retained separately for data extraction and analysis. Control node: placebo, usual diet, and no additional nutritional supplementation were grouped because none provided the target active nutritional supplementation, and within each randomized trial the non-nutritional background conditions were required to be balanced or comparable across arms. The specific control type was considered in the assessment of transitivity.

Supplementary Table S2. Intervention node classification for network meta-analysis

Node	Full name	Nutritional components and dosage	Included studies/arms
Control	Placebo, usual diet, or no additional nutritional supplementation	Placebo, regular diet, no nutritional supplementation	All included studies
WP+VD+Leu	Whey protein + vitamin D + leucine	8.5g whey protein, 120IU vitamin D, 1.2g leucine per day; 20g whey protein, 800 IU vitamin D, 3g leucine, twice daily; 20g whey protein, 800IU vitamin D, 2.8g leucine, twice daily; 22g whey protein, 100IU vitamin D, 4g leucine per day	Lin 2021; Bauer 2015; Rondanelli 2020; Rondanelli 2016
WP+VD+VE	Whey protein + vitamin D + vitamin E	22g whey protein, 702IU vitamin D, 109mg vitamin E, twice daily	Bo 2019
ω-3+Leu+PS23	Omega-3 fatty acids + leucine + Lactobacillus paracasei PS23	2.5g omega-3, 500mg leucine, 3.0 × 10^9CFU PS23, per day	Rondanelli 2022
WP+Leu	Whey protein + leucine	10.6g whey protein, 3.1 g leucine, twice daily; 25.3g whey protein, 3g leucine, twice daily	Murphy 2021 (WP+Leu arm); Chung 2026
WP+Leu+LCT	Whey protein + leucine + long-chain lipid formulation containing n-3 PUFA	10.6g whey protein, 3.1 g leucine, twice daily; 2.4g n-3 PUFA	Murphy 2021 (WP+Leu+LCT arm)
WP	Whey protein alone	20g Whey protein, twice daily; 20g Whey protein, per day	Björkman 2020 (WP arm); Park 2018 (WP arm)
P	Protein (non-whey or mixed protein)	3.75g protein, twice daily; 20g protein, twice daily; 15.7g protein per day, 7 times a week; 24–30g protein	Björkman 2020 (P arm); Ottestad 2017; Alemán-Mateo 2012; Kao 2025; Park 2018 (P arm)

		per day; 14g protein per day	
Leu	Leucine alone	3g leucine, twice daily	Martínez-Arnau 2020
VD	Vitamin D alone	10,000IU vitamin D, 3 times a week	El Hajj 2018
Leu+VD+MCT	Leucine + vitamin D + medium-chain triglycerides	1.2g leucine, 800IU vitamin D, 6g MCT per day	Abe 2016 (Leu+VD+MCT arm)
Leu+VD+LCT	Leucine + vitamin D + long-chain triglycerides	1.2g leucine, 800IU vitamin D, 6g LCT per day	Abe 2016 (Leu+VD+LCT arm)
HMB+ARG+LYS	β-Hydroxy-β-methylbutyrate + arginine + lysine	2 g HMB, 5g arginine, 1.5g lysine per day	Flakoll 2004
Ca+HMB	Calcium + β-hydroxy-β-methylbutyrate	207mg calcium, 1200mg HMB per day	Osuka 2021
P+Ca	Protein + calcium (original cheese)	9.0 g protein + 322.8 mg calcium per day	Chen 2024 (original cheese arm)
P+Ca+CaP	Protein + calcium (golden cheese)	12.7 g protein + 802.1 mg calcium + additional calcium-containing phosphates (CaP), per day	Chen 2024 (golden cheese arm)
WP+CaHMB	Whey protein + calcium HMB	660 kcal, 40g protein, 3.0g CaHMB per day	Cramer (sarcopenia subgroup), 2016 Cramer (at-risk sarcopenia subgroup), 2016

Notes: WP+VD+VE, whey protein + vitamin D + vitamin E; ω-3+Leu+PS23, ω-3 fatty acids + leucine + Lactobacillus paracasei PS23; WP+VD+Leu, whey protein + vitamin D + leucine; WP, whey protein; Leu, leucine; VD, vitamin D; P+Ca, protein + calcium; P+Ca+CaP, protein + calcium + calcium-containing phosphates; WP+Leu, whey protein + leucine; WP+Leu+LCT, whey protein + leucine + long-chain lipid formulation containing n-3 PUFA; Leu+VD+MCT, leucine + vitamin D + medium-chain triglycerides; Leu+VD+LCT, leucine + vitamin D + long-chain triglycerides; HMB+ARG+LYS, β-hydroxy-β-methylbutyrate + arginine + lysine; Ca+HMB, calcium + β-hydroxy-β-methylbutyrate; P, protein; WP+CaHMB, whey protein + calcium β-hydroxy-β-methylbutyrate.

Supplementary Table S3. Distribution of potential effect modifiers across indirect comparisons for appendicular skeletal muscle mass index (ASMI)

Indirect comparison	Node A	Node B	Age (A)	Age (B)	Sarcopenia status (A)	Sarcopenia status (B)	Intervention duration (A)	Intervention duration (B)	Background exercise (A)	Background exercise (B)	Supporting studies (A)	Supporting studies (B)
WP+VD+Leu vs WP+Leu	WP+VD+Leu	WP+Leu	IG: 72.50±5.57 / CG: 73.80±8.11; IG: 77.30±6.7 / CG: 78.10±7.0; IG: 81±7 / CG: 82±5; IG: 80.77±6.29 / CG: 80.21±8.54 (n=4 study records)	LEU-PRO+n-3: 73±6 / LEU-PRO: 70±5 / CG: 73±7; IG: 68.80±6.5 / CG: 69.70±7.9 (n=2 study records)	Confirmed (4 study records)	At risk: 1; Confirmed: 1 study record	Lin 2021: 12 weeks; Bauer, 2015: 13 weeks; Rondanelli 2020: 6 weeks; Rondanelli 2016: 12 weeks	Murphy 2021: 24 weeks; Chung 2026: 12 weeks	Lin, 2021: No; Bauer, 2015: No; Rondanelli, 2020: Personalized physical rehabilitation therapy 5 times a week, 20–30 minutes per session; Rondanelli, 2016: Structured training 5 times a week, 20 minutes each session	Murphy, 2021: No; Chung, 2026: No	Lin, 2021; Bauer, 2015; Rondanelli, 2020; Rondanelli, 2016	Murphy, 2021; Chung, 2026
WP+VD+Leu vs Leu	WP+VD+Leu	Leu	IG: 72.50±5.57 / CG: 73.80±8.11; IG: 77.30±6.7 / CG: 78.10±7.0; IG: 81±7 / CG:	IG: 78.70±21.2 / CG: 78.0±21.6 (n=1 study record)	Confirmed (4 study records)	Confirmed (1 study record)	Lin 2021: 12 weeks; Bauer, 2015: 13 weeks; Rondanelli 2020: 6 weeks; Rondanelli	Martínez-Arnau 2020: 13 weeks	Lin, 2021: No; Bauer, 2015: No; Rondanelli, 2020: Personalized physical rehabilitation	Martínez-Arnau, 2020: No	Lin, 2021; Bauer, 2015; Rondanelli, 2020; Rondanelli, 2016	Martínez-Arnau, 2020

Indirect comparison	Node A	Node B	Age (A)	Age (B)	Sarcopenia status (A)	Sarcopenia status (B)	Intervention duration (A)	Intervention duration (B)	Background exercise (A)	Background exercise (B)	Supporting studies (A)	Supporting studies (B)
			82±5; IG: 80.77±6.29 / CG: 80.21±8.54 (n=4 study records)				2016: 12 weeks		therapy 5 times a week, 20–30 minutes per session; Rondanelli, 2016: Structured training 5 times a week, 20 minutes per session			
WP+VD+Leu vs WP+VD+VE	WP+VD+Leu	WP+VD+VE	IG: 72.50±5.57 / CG: 73.80±8.11; IG: 77.30±6.7 / CG: 78.10±7.0; IG: 81±7 / CG: 82±5; IG: 80.77±6.29 / CG: 80.21±8.54 (n=4 study records)	IG: 73.23±6.52 / CG: 74.83±5.94 (n=1 study record)	Confirmed (4 study records)	Confirmed (1 study record)	Lin 2021: 12 weeks; Bauer, 2015: 13 weeks; Rondanelli 2020: 6 weeks; Rondanelli 2016: 12 weeks	Bo, 2019: 24 weeks	Lin, 2021: No; Bauer, 2015: No; Rondanelli, 2020: Personalized physical rehabilitation therapy 5 times a week, 20–30 minutes per session; Rondanelli, 2016: Structured training 5 times a week, 20 minutes each session	Bo, 2019: No	Lin, 2021; Bauer, 2015; Rondanelli, 2020; Rondanelli, 2016	Bo, 2019

Indirect comparison	Node A	Node B	Age (A)	Age (B)	Sarcopenia status (A)	Sarcopenia status (B)	Intervention duration (A)	Intervention duration (B)	Background exercise (A)	Background exercise (B)	Supporting studies (A)	Supporting studies (B)
WP+VD+Leu vs WP	WP+VD+Leu	WP	IG: 72.50±5.57 / CG: 73.80±8.11; IG: 77.30±6.7 / CG: 78.10±7.0; IG: 81±7 / CG: 82±5; IG: 80.77±6.29 / CG: 80.21±8.54 (n=4 study records)	WP: 83.60±4.7 / P: 84.0±3.9 / CG: 83.70±5.1; WP: 76.80±3.70 / P: 77.30±3.67 / CG: 76.83±3.86 (n=2 study records)	Confirmed (4 study records)	Confirmed: 1; At risk: 1 study record	Lin 2021: 12 weeks; Bauer, 2015: 13 weeks; Rondanelli 2020: 6 weeks; Rondanelli 2016: 12 weeks	Björkman 2020: 48 weeks; Park, 2018: 12 weeks	Lin, 2021: No; Bauer, 2015: No; Rondanelli, 2020: Personalized physical rehabilitation therapy 5 times a week, 20–30 minutes per session; Rondanelli, 2016: Structured training 5 times a week, 20 minutes each session	Björkman, 2020: Low-Intensity Home Workouts; Park, 2018: No	Lin, 2021; Bauer, 2015; Rondanelli, 2020; Rondanelli, 2016	Björkman, 2020; Park, 2018
WP+VD+Leu vs ω-3+Leu+PS23	WP+VD+Leu	ω-3+Leu+PS23	IG: 72.50±5.57 / CG: 73.80±8.11; IG: 77.30±6.7 / CG: 78.10±7.0; IG: 81±7 / CG: 82±5; IG: 80.77±6.29 / CG: 80.21±8.54	IG: 78.84±5.80 / CG: 80.50±3.74 (n=1 study record)	Confirmed (4 study records)	Confirmed (1 study record)	Lin 2021: 12 weeks; Bauer, 2015: 13 weeks; Rondanelli 2020: 6 weeks; Rondanelli 2016: 12 weeks	Rondanelli 2022: 8 weeks	Lin, 2021: No; Bauer, 2015: No; Rondanelli, 2020: Personalized physical rehabilitation therapy 5 times a week, 20–30 minutes per	Rondanelli, 2022: Basic exercise 5 times a week for 20 to 30 minutes	Lin, 2021; Bauer, 2015; Rondanelli, 2020; Rondanelli, 2016	Rondanelli, 2022

Indirect comparison	Node A	Node B	Age (A)	Age (B)	Sarcopenia status (A)	Sarcopenia status (B)	Intervention duration (A)	Intervention duration (B)	Background exercise (A)	Background exercise (B)	Supporting studies (A)	Supporting studies (B)
			(n=4 study records)						session; Rondanelli, 2016: Structured training 5 times a week, 20 minutes each session			
WP+VD+Leu vs Ca+HMB	WP+VD+Leu	Ca+HMB	IG: 72.50±5.57 / CG: 73.80±8.11; IG: 77.30±6.7 / CG: 78.10±7.0; IG: 81±7 / CG: 82±5; IG: 80.77±6.29 / CG: 80.21±8.54 (n=4 study records)	IG: 71.50±4.5 / CG: 71.60±4.2 (n=1 study record)	Confirmed (4 study records)	Confirmed (1 study record)	Lin 2021: 12 weeks; Bauer, 2015: 13 weeks; Rondanelli 2020: 6 weeks; Rondanelli 2016: 12 weeks	Osuka 2021: 12 weeks	Lin, 2021: No; Bauer, 2015: No; Rondanelli, 2020: Personalized physical rehabilitation therapy 5 times a week, 20–30 minutes per session; Rondanelli, 2016: Structured training 5 times a week, 20 minutes each session	Osuka, 2021: No	Lin, 2021; Bauer, 2015; Rondanelli, 2020; Rondanelli, 2016	Osuka, 2021
P vs Leu	P	Leu	WP: 83.60±4.7 / P: 84.0±3.9 / CG:	IG: 78.70±21.2 / CG: 78.0±21.	Confirmed: 2; At risk: 3 study records	Confirmed (1 study record)	Björkman 2020: 48 weeks; Ottestad,	Martínez-Arnau 2020: 13 weeks	Björkman, 2020: Low-Intensity Home	Martínez-Arnau, 2020: No	Björkman, 2020; Ottestad, 2017;	Martínez-Arnau, 2020

Indirect comparison	Node A	Node B	Age (A)	Age (B)	Sarcopenia status (A)	Sarcopenia status (B)	Intervention duration (A)	Intervention duration (B)	Background exercise (A)	Background exercise (B)	Supporting studies (A)	Supporting studies (B)
			83.70±5.1; IG: 76.80±6.2 / CG: 77.10±4.7; IG: 75.40±5.0 / CG: 76.70±5.8; IG: 72.60±5.1 / CG: 72.30±5.2; WP: 76.80±3.70 / P: 77.30±3.67 / CG: 76.83±3.86 (n=5 study records)	6 (n=1 study record)			2017: 12 weeks; Alemán-Mateo, 2012: 12 weeks; Kao 2025: 12 weeks; Park, 2018: 12 weeks		Workouts; Ottestad, 2017: No; Alemán-Mateo, 2012: No; Kao, 2025: No; Park, 2018: No		Alemán-Mateo, 2012; Kao, 2025; Park, 2018	
P vs WP+VD+VE	P	WP+VD+VE	WP: 83.60±4.7 / P: 84.0±3.9 / CG: 83.70±5.1; IG: 76.80±6.2 / CG: 77.10±4.7; IG: 75.40±5.0 / CG: 76.70±5.8; IG: 72.60±5.1 / CG: 72.30±5.2; WP:	IG: 73.23±6.52 / CG: 74.83±5.94 (n=1 study record)	Confirmed: 2; At risk: 3 study records	Confirmed (1 study record)	Björkman 2020: 48 weeks; Ottestad, 2017: 12 weeks; Alemán-Mateo, 2012: 12 weeks; Kao 2025: 12 weeks; Park, 2018: 12 weeks	Bo, 2019: 24 weeks	Björkman, 2020: Low-Intensity Home Workouts; Ottestad, 2017: No; Alemán-Mateo, 2012: No; Kao, 2025: No; Park, 2018: No	Bo, 2019: No	Björkman, 2020; Ottestad, 2017; Alemán-Mateo, 2012; Kao, 2025; Park, 2018	Bo, 2019

Indirect comparison	Node A	Node B	Age (A)	Age (B)	Sarcopenia status (A)	Sarcopenia status (B)	Intervention duration (A)	Intervention duration (B)	Background exercise (A)	Background exercise (B)	Supporting studies (A)	Supporting studies (B)
			76.80±3.70 / P: 77.30±3.67 / CG: 76.83±3.86 (n=5 study records)									
P vs WP	P	WP	WP: 83.60±4.7 / P: 84.0±3.9 / CG: 83.70±5.1; IG: 76.80±6.2 / CG: 77.10±4.7; IG: 75.40±5.0 / CG: 76.70±5.8; IG: 72.60±5.1 / CG: 72.30±5.2; WP: 76.80±3.70 / P: 77.30±3.67 / CG: 76.83±3.86 (n=5 study records)	WP: 83.60±4.7 / P: 84.0±3.9 / CG: 83.70±5.1; WP: 76.80±3.70 / P: 77.30±3.67 / CG: 76.83±3.86 (n=2 study records)	Confirmed: 2; At risk: 3 study records	Confirmed: 1; At risk: 1 study record	Björkman 2020: 48 weeks; Ottestad, 2017: 12 weeks; Alemán-Mateo, 2012: 12 weeks; Kao 2025: 12 weeks; Park, 2018: 12 weeks	Björkman, 2020: 48 weeks; Park, 2018: 12 weeks	Björkman, 2020: Low-Intensity Home Workouts; Ottestad, 2017: No; Alemán-Mateo, 2012: No; Kao, 2025: No; Park, 2018: No	Björkman, 2020: Low-Intensity Home Workouts; Park, 2018: No	Björkman, 2020; Ottestad, 2017; Alemán-Mateo, 2012; Kao, 2025; Park, 2018	Björkman, 2020; Park, 2018
P vs ω-3+Leu+PS23	P	ω-3+Leu +PS23	WP: 83.60±4.7 / P: 84.0±3.9 / CG: 83.70±5.1; IG:	IG: 78.84±5.80 / CG: 80.50±3.74 (n=1	Confirmed: 2; At risk: 3 study records	Confirmed (1 study record)	Björkman 2020: 48 weeks; Ottestad, 2017: 12	Rondanelli, 2022: 8 weeks	Björkman, 2020: Low-Intensity Home Workouts;	Rondanelli, 2022: Basic exercise 5 times a week	Björkman, 2020; Ottestad, 2017; Alemán-	Rondanelli, 2022

Indirect comparison	Node A	Node B	Age (A)	Age (B)	Sarcopenia status (A)	Sarcopenia status (B)	Intervention duration (A)	Intervention duration (B)	Background exercise (A)	Background exercise (B)	Supporting studies (A)	Supporting studies (B)
			76.80±6.2 / CG: 77.10±4.7; IG: 75.40±5.0 / CG: 76.70±5.8; IG: 72.60±5.1 / CG: 72.30±5.2; WP: 76.80±3.70 / P: 77.30±3.67 / CG: 76.83±3.86 (n=5 study records)	study record)			weeks; Alemán-Mateo, 2012: 12 weeks; Kao 2025: 12 weeks; Park, 2018: 12 weeks		Ottestad, 2017: No; Alemán-Mateo, 2012: No; Kao, 2025: No; Park, 2018: No	for 20 to 30 minutes	Mateo, 2012; Kao, 2025; Park, 2018	
P vs Ca+HMB	P	Ca+HMB	WP: 83.60±4.7 / P: 84.0±3.9 / CG: 83.70±5.1; IG: 76.80±6.2 / CG: 77.10±4.7; IG: 75.40±5.0 / CG: 76.70±5.8; IG: 72.60±5.1 / CG: 72.30±5.2; WP: 76.80±3.70 /	IG: 71.50±4.5 / CG: 71.60±4.2 (n=1 study record)	Confirmed: 2; At risk: 3 study records	Confirmed (1 study record)	Björkman 2020: 48 weeks; Ottestad, 2017: 12 weeks; Alemán-Mateo, 2012: 12 weeks; Kao 2025: 12 weeks; Park, 2018: 12 weeks	Osuka, 2021: 12 weeks	Björkman, 2020: Low-Intensity Home Workouts; Ottestad, 2017: No; Alemán-Mateo, 2012: No; Kao, 2025: No; Park, 2018: No	Osuka, 2021: No	Björkman, 2020; Ottestad, 2017; Alemán-Mateo, 2012; Kao, 2025; Park, 2018	Osuka, 2021

Indirect comparison	Node A	Node B	Age (A)	Age (B)	Sarcopenia status (A)	Sarcopenia status (B)	Intervention duration (A)	Intervention duration (B)	Background exercise (A)	Background exercise (B)	Supporting studies (A)	Supporting studies (B)
			P: 77.30±3.67 / CG: 76.83±3.86 (n=5 study records)									
WP+Leu vs Leu	WP+Leu	Leu	LEU-PRO+n-3: 73±6 / LEU-PRO: 70±5 / CG: 73±7; IG: 68.80±6.5 / CG: 69.70±7.9 (n=2 study records)	IG: 78.70±21.2 / CG: 78.0±21.6 (n=1 study record)	At risk: 1; Confirmed: 1 study record	Confirmed (1 study record)	Murphy 2021: 24 weeks; Chung 2026: 12 weeks	Martínez-Arnau 2020: 13 weeks	Murphy, 2021: No; Chung, 2026: No	Martínez-Arnau, 2020: No	Murphy, 2021; Chung, 2026	Martínez-Arnau, 2020
WP+Leu vs WP+VD+VE	WP+Leu	WP+VD+VE	LEU-PRO+n-3: 73±6 / LEU-PRO: 70±5 / CG: 73±7; IG: 68.80±6.5 / CG: 69.70±7.9 (n=2 study records)	IG: 73.23±6.52 / CG: 74.83±5.94 (n=1 study record)	At risk: 1; Confirmed: 1 study record	Confirmed (1 study record)	Murphy 2021: 24 weeks; Chung 2026: 12 weeks	Bo, 2019: 24 weeks	Murphy, 2021: No; Chung, 2026: No	Bo, 2019: No	Murphy, 2021; Chung, 2026	Bo, 2019
WP+Leu vs WP	WP+Leu	WP	LEU-PRO+n-3: 73±6 / LEU-PRO: 70±5 / CG: 73±7; IG: 68.80±6.5 / CG: 69.70±7.9 (n=2 study records)	WP: 83.60±4.7 / P: 84.0±3.9 / CG: 83.70±5.1; WP: 76.80±3.70 / P:	At risk: 1; Confirmed: 1 study record	Confirmed: 1; At risk: 1 study record	Murphy 2021: 24 weeks; Chung 2026: 12 weeks	Björkman 2020: 48 weeks; Park, 2018: 12 weeks	Murphy, 2021: No; Chung, 2026: No	Björkman, 2020: Low-Intensity Home Workouts; Park, 2018: No	Murphy, 2021; Chung, 2026	Björkman, 2020; Park, 2018

Indirect comparison	Node A	Node B	Age (A)	Age (B)	Sarcopenia status (A)	Sarcopenia status (B)	Intervention duration (A)	Intervention duration (B)	Background exercise (A)	Background exercise (B)	Supporting studies (A)	Supporting studies (B)
				77.30±3.67 / CG: 76.83±3.86 (n=2 study records)								
WP+Leu vs ω-3+Leu+PS23	WP+Leu	ω-3+Leu+PS23	LEU-PRO+n-3: 73±6 / LEU-PRO: 70±5 / CG: 73±7; IG: 68.80±6.5 / CG: 69.70±7.9 (n=2 study records)	IG: 78.84±5.80 / CG: 80.50±3.74 (n=1 study record)	At risk: 1; Confirmed: 1 study record	Confirmed (1 study record)	Murphy 2021: 24 weeks; Chung 2026: 12 weeks	Rondanelli 2022: 8 weeks	Murphy, 2021: No; Chung, 2026: No	Rondanelli, 2022: Basic exercise 5 times a week for 20 to 30 minutes	Murphy, 2021; Chung, 2026	Rondanelli, 2022
WP+Leu vs Ca+HMB	WP+Leu	Ca+HMB	LEU-PRO+n-3: 73±6 / LEU-PRO: 70±5 / CG: 73±7; IG: 68.80±6.5 / CG: 69.70±7.9 (n=2 study records)	IG: 71.50±4.5 / CG: 71.60±4.2 (n=1 study record)	At risk: 1; Confirmed: 1 study record	Confirmed (1 study record)	Murphy 2021: 24 weeks; Chung 2026: 12 weeks	Osuka 2021: 12 weeks	Murphy, 2021: No; Chung, 2026: No	Osuka, 2021: No	Murphy, 2021; Chung, 2026	Osuka, 2021
Leu vs WP+VD+VE	Leu	WP+VD+VE	IG: 78.70±21.2 / CG: 78.0±21.6 (n=1 study record)	IG: 73.23±6.52 / CG: 74.83±5.94 (n=1 study record)	Confirmed (1 study record)	Confirmed (1 study record)	Martínez-Arnau 2020: 13 weeks	Bo, 2019: 24 weeks	Martínez-Arnau, 2020: No	Bo, 2019: No	Martínez-Arnau, 2020	Bo, 2019

Indirect comparison	Node A	Node B	Age (A)	Age (B)	Sarcopenia status (A)	Sarcopenia status (B)	Intervention duration (A)	Intervention duration (B)	Background exercise (A)	Background exercise (B)	Supporting studies (A)	Supporting studies (B)
Leu vs WP	Leu	WP	IG: 78.70±21.2 / CG: 78.0±21.6 (n=1 study record)	WP: 83.60±4.7 / P: 84.0±3.9 / CG: 83.70±5.1; WP: 76.80±3.70 / P: 77.30±3.67 / CG: 76.83±3.86 (n=2 study records)	Confirmed (1 study record)	Confirmed: 1; At risk: 1 study record	Martínez-Arnau 2020: 13 weeks	Björkman 2020: 48 weeks; Park, 2018: 12 weeks	Martínez-Arnau, 2020: No	Björkman, 2020: Low-Intensity Home Workouts; Park, 2018: No	Martínez-Arnau, 2020	Björkman, 2020; Park, 2018
Leu vs ω-3+Leu+PS23	Leu	ω-3+Leu +PS23	IG: 78.70±21.2 / CG: 78.0±21.6 (n=1 study record)	IG: 78.84±5.80 / CG: 80.50±3.74 (n=1 study record)	Confirmed (1 study record)	Confirmed (1 study record)	Martínez-Arnau 2020: 13 weeks	Rondanelli 2022: 8 weeks	Martínez-Arnau, 2020: No	Rondanelli, 2022: Basic exercise 5 times a week for 20 to 30 minutes	Martínez-Arnau, 2020	Rondanelli, 2022
Leu vs Ca+HMB	Leu	Ca+HMB	IG: 78.70±21.2 / CG: 78.0±21.6 (n=1 study record)	IG: 71.50±4.5 / CG: 71.60±4.2 (n=1 study record)	Confirmed (1 study record)	Confirmed (1 study record)	Martínez-Arnau 2020: 13 weeks	Osuka 2021: 12 weeks	Martínez-Arnau, 2020: No	Osuka, 2021: No	Martínez-Arnau, 2020	Osuka, 2021
WP+VD+VE vs WP	WP+VD+VE	WP	IG: 73.23±6.52 / CG:	WP: 83.60±4.7 / P:	Confirmed (1 study record)	Confirmed: 1; At risk: 1	Bo, 2019: 24 weeks	Björkman 2020: 48 weeks; Park,	Bo, 2019: No	Björkman, 2020: Low-Intensity	Bo, 2019	Björkman, 2020; Park, 2018

**Indirect comparison**	**Node A**	**Node B**	**Age (A)**	**Age (B)**	**Sarcopenia status (A)**	**Sarcopenia status (B)**	**Intervention duration (A)**	**Intervention duration (B)**	**Background exercise (A)**	**Background exercise (B)**	**Supporting studies (A)**	**Supporting studies (B)**
			74.83±5.94 (n=1 study record)	84.0±3.9 / CG: 83.70±5.1; WP: 76.80±3.70 / P: 77.30±3.67 / CG: 76.83±3.86 (n=2 study records)		study record		2018: 12 weeks		Home Workouts; Park, 2018: No		
WP+VD+VE vs ω-3+Leu+PS23	WP+VD+VE	ω-3+Leu+PS23	IG: 73.23±6.52 / CG: 74.83±5.94 (n=1 study record)	IG: 78.84±5.80 / CG: 80.50±3.74 (n=1 study record)	Confirmed (1 study record)	Confirmed (1 study record)	Bo, 2019: 24 weeks	Rondanelli 2022: 8 weeks	Bo, 2019: No	Rondanelli, 2022: Basic exercise 5 times a week for 20 to 30 minutes	Bo, 2019	Rondanelli, 2022
WP+VD+VE vs Ca+HMB	WP+VD+VE	Ca+HMB	IG: 73.23±6.52 / CG: 74.83±5.94 (n=1 study record)	IG: 71.50±4.5 / CG: 71.60±4.2 (n=1 study record)	Confirmed (1 study record)	Confirmed (1 study record)	Bo, 2019: 24 weeks	Osuka, 2021: 12 weeks	Bo, 2019: No	Osuka, 2021: No	Bo, 2019	Osuka, 2021
WP vs ω-3+Leu+PS23	WP	ω-3+Leu+PS23	WP: 83.60±4.7 / P: 84.0±3.9 / CG: 83.70±5.1; WP:	IG: 78.84±5.80 / CG: 80.50±3.74 (n=1	Confirmed: 1; At risk: 1 study record	Confirmed (1 study record)	Björkman 2020: 48 weeks; Park, 2018: 12 weeks	Rondanelli 2022: 8 weeks	Björkman, 2020: Low-Intensity Home Workouts;	Rondanelli, 2022: Basic exercise 5 times a week for 20 to 30 minutes	Björkman, 2020; Park, 2018	Rondanelli, 2022

Indirect comparison	Node A	Node B	Age (A)	Age (B)	Sarcopenia status (A)	Sarcopenia status (B)	Intervention duration (A)	Intervention duration (B)	Background exercise (A)	Background exercise (B)	Supporting studies (A)	Supporting studies (B)
			76.80±3.70 / P: 77.30±3.67 / CG: 76.83±3.86 (n=2 study records)	study record)					Park, 2018: No			
WP vs Ca+HMB	WP	Ca+HMB	WP: 83.60±4.7 / P: 84.0±3.9 / CG: 83.70±5.1; WP: 76.80±3.70 / P: 77.30±3.67 / CG: 76.83±3.86 (n=2 study records)	IG: 71.50±4.5 / CG: 71.60±4.2 (n=1 study record)	Confirmed: 1; At risk: 1 study record	Confirmed (1 study record)	Björkman 2020: 48 weeks; Park, 2018: 12 weeks	Osuka 2021: 12 weeks	Björkman, 2020: Low-Intensity Home Workouts; Park, 2018: No	Osuka, 2021: No	Björkman, 2020; Park, 2018	Osuka, 2021
ω-3+Leu+PS23 vs Ca+HMB	ω-3+Leu+PS23	Ca+HMB	IG: 78.84±5.80 / CG: 80.50±3.74 (n=1 study record)	IG: 71.50±4.5 / CG: 71.60±4.2 (n=1 study record)	Confirmed (1 study record)	Confirmed (1 study record)	Rondanelli 2022: 8 weeks	Osuka 2021: 12 weeks	Rondanelli, 2022: Basic exercise 5 times a week for 20 to 30 minutes	Osuka, 2021: No	Rondanelli, 2022	Osuka, 2021

Supplementary Table S4. Distribution of potential effect modifiers across indirect comparisons for grip strength

Indirect comparison	Node A	Node B	Age (A)	Age (B)	Sarcopenia status (A)	Sarcopenia status (B)	Intervention duration (A)	Intervention duration (B)	Background exercise (A)	Background exercise (B)	Supporting studies (A)	Supporting studies (B)
Leu vs WP	Leu	WP	IG: 78.70±21.2 / CG: 78.0±21.6	Björkman 2020: WP 83.60±4.7 / P 84.0±3.9 / CG 83.70±5.1; Park 2018: WP 76.80±3.70 / P 77.30±3.67 / CG 76.83±3.86	Confirmed	Confirmed: 1; At risk: 1	Martínez-Arnau 2020: 13 weeks	Björkman 2020: 48 weeks; Park 2018: 12 weeks	Martínez-Arnau 2020: No	Björkman 2020: Low-intensity home workouts; Park 2018: No	Martínez-Arnau 2020	Björkman 2020; Park 2018
Leu vs WP+VD+Leu	Leu	WP+VD+Leu	IG: 78.70±21.2 / CG: 78.0±21.6	Lin 2021: IG 72.50±5.57 / CG 73.80±8.11 ; Bauer 2015: IG 77.30±6.7 / CG 78.10±7.0; Rondanelli 2020: IG 81±7 / CG 82±5; Rondanelli 2016: IG	Confirmed	Confirmed (4 studies)	Martínez-Arnau 2020: 13 weeks	Lin 2021: 12 weeks; Bauer 2015: 13 weeks; Rondanelli 2020: 6 weeks; Rondanelli 2016: 12 weeks	Martínez-Arnau 2020: No	Lin 2021: No; Bauer 2015: No; Rondanelli 2020: Personalized physical rehabilitation therapy 5 times/week, 20–30 min/session; Rondanelli 2016: Structured training 5	Martínez-Arnau 2020	Lin 2021; Bauer 2015; Rondanelli 2020; Rondanelli 2016

Indirect comparison	Node A	Node B	Age (A)	Age (B)	Sarcopenia status (A)	Sarcopenia status (B)	Intervention duration (A)	Intervention duration (B)	Background exercise (A)	Background exercise (B)	Supporting studies (A)	Supporting studies (B)
				80.77±6.29 / CG 80.21±8.54						times/week, 20 min/session		
Leu vs ω-3+Leu+PS23	Leu	ω-3+Leu +PS23	IG: 78.70±21.2 / CG: 78.0±21.6	IG: 78.84±5.80 / CG: 80.50±3.74	Confirmed	Confirmed	Martínez-Arnau 2020: 13 weeks	Rondanelli 2022: 8 weeks	Martínez-Arnau 2020: No	Rondanelli 2022: Basic exercise 5 times/week, 20–30 min	Martínez-Arnau 2020	Rondanelli 2022
Leu vs WP+VD+VE	Leu	WP+VD+VE	IG: 78.70±21.2 / CG: 78.0±21.6	IG: 73.23±6.52 / CG: 74.83±5.94	Confirmed	Confirmed	Martínez-Arnau 2020: 13 weeks	Bo 2019: 24 weeks	Martínez-Arnau 2020: No	Bo 2019: No	Martínez-Arnau 2020	Bo 2019
Leu vs VD	Leu	VD	IG: 78.70±21.2 / CG: 78.0±21.6	IG: 73.05±1.95 / CG: 73.56±2.14	Confirmed	At risk	Martínez-Arnau 2020: 13 weeks	El Hajj 2018: 24 weeks	Martínez-Arnau 2020: No	El Hajj 2018: No	Martínez-Arnau 2020	El Hajj 2018
Leu vs P+Ca+CaP	Leu	P+Ca+CaP	IG: 78.70±21.2 / CG: 78.0±21.6	Chen 2024: OG 71(67,73) / GG 75(70,77) / CG 73(67,76)	Confirmed	At risk	Martínez-Arnau 2020: 13 weeks	Chen 2024: 12 weeks	Martínez-Arnau 2020: No	Chen 2024: Maintain current physical activity	Martínez-Arnau 2020	Chen 2024
Leu vs WP+Leu	Leu	WP+Leu	IG: 78.70±21.2 / CG: 78.0±21.6	Murphy 2021: LEU-PRO+n-3 73±6 / LEU-PRO 70±5 / CG 73±7; Chung	Confirmed	At risk: 1; Confirmed: 1	Martínez-Arnau 2020: 13 weeks	Murphy 2021: 24 weeks; Chung 2026: 12 weeks	Martínez-Arnau 2020: No	Murphy 2021: No; Chung 2026: No	Martínez-Arnau 2020	Murphy 2021; Chung 2026

Indirect comparison	Node A	Node B	Age (A)	Age (B)	Sarcopenia status (A)	Sarcopenia status (B)	Intervention duration (A)	Intervention duration (B)	Background exercise (A)	Background exercise (B)	Supporting studies (A)	Supporting studies (B)
Leu vs WP+Leu+LCT	Leu	WP+Leu+LCT	IG: 78.70±21.2 / CG: 78.0±21.6	2026: IG 68.80±6.5 / CG 69.70±7.9 Murphy 2021: LEU-PRO+n-3 73±6 / LEU-PRO 70±5 / CG 73±7	Confirmed	At risk	Martínez-Arnau 2020: 13 weeks	Murphy 2021: 24 weeks	Martínez-Arnau 2020: No	Murphy 2021: No	Martínez-Arnau 2020	Murphy 2021
Leu vs Leu+VD+MCT	Leu	Leu+VD+MCT	IG: 78.70±21.2 / CG: 78.0±21.6	Abe 2016: LD+MCT 85.50±3.7 / LD+LCT 87.70±3.9 / CG 86.80±6.5	Confirmed	At risk	Martínez-Arnau 2020: 13 weeks	Abe 2016: 12 weeks	Martínez-Arnau 2020: No	Abe 2016: Regular exercise activities in nursing homes	Martínez-Arnau 2020	Abe 2016
Leu vs Leu+VD+LCT	Leu	Leu+VD+LCT	IG: 78.70±21.2 / CG: 78.0±21.6	Abe 2016: LD+MCT 85.50±3.7 / LD+LCT 87.70±3.9 / CG 86.80±6.5	Confirmed	At risk	Martínez-Arnau 2020: 13 weeks	Abe 2016: 12 weeks	Martínez-Arnau 2020: No	Abe 2016: Regular exercise activities in nursing homes	Martínez-Arnau 2020	Abe 2016
Leu vs HMB+ARG+LYS	Leu	HMB+ARG+LYS	IG: 78.70±21.2 / CG: 78.0±21.6	IG: 71.50±1.5 / CG: 71.50±1.5	Confirmed	At risk	Martínez-Arnau 2020: 13 weeks	Flakoll 2004: 12 weeks	Martínez-Arnau 2020: No	Flakoll 2004: No	Martínez-Arnau 2020	Flakoll 2004
Leu vs Ca+HMB	Leu	Ca+HMB	IG: 78.70±21.2 /	IG: 71.50±4.5 /	Confirmed	Confirmed	Martínez-Arnau 2020:	Osuka 2021: 12 weeks	Martínez-Arnau 2020:	Osuka 2021: No	Martínez-Arnau 2020	Osuka 2021

Indirect comparison	Node A	Node B	Age (A)	Age (B)	Sarcopenia status (A)	Sarcopenia status (B)	Intervention duration (A)	Intervention duration (B)	Background exercise (A)	Background exercise (B)	Supporting studies (A)	Supporting studies (B)
			CG: 78.0±21.6	CG: 71.60±4.2			13 weeks		No			
Leu vs P	Leu	P	IG: 78.70±21.2 / CG: 78.0±21.6	Björkman 2020: WP 83.60±4.7 / P 84.0±3.9 / CG 83.70±5.1; Ottestad 2017: IG 76.80±6.2 / CG 77.10±4.7; Alemán-Mateo 2012: IG 75.40±5.0 / CG 76.70±5.8; Kao 2025: IG 72.60±5.1 / CG 72.30±5.2; Park 2018: WP 76.80±3.70 / P 77.30±3.67 / CG 76.83±3.86	Confirmed	Confirmed: 2; At risk: 3	Martínez-Arnau 2020: 13 weeks	Björkman 2020: 48 weeks; Ottestad 2017: 12 weeks; Alemán-Mateo 2012: 12 weeks; Kao 2025: 12 weeks; Park 2018: 12 weeks	Martínez-Arnau 2020: No	Björkman 2020: Low-intensity home workouts; Ottestad 2017: No; Alemán-Mateo 2012: No; Kao 2025: No; Park 2018: No	Martínez-Arnau 2020	Björkman 2020; Ottestad 2017; Alemán-Mateo 2012; Kao 2025; Park 2018
Leu vs P+Ca	Leu	P+Ca	IG:	Chen	Confirmed	At risk	Martínez-	Chen 2024:	Martínez-	Chen 2024:	Martínez-	Chen 2024

Indirect comparison	Node A	Node B	Age (A)	Age (B)	Sarcopenia status (A)	Sarcopenia status (B)	Intervention duration (A)	Intervention duration (B)	Background exercise (A)	Background exercise (B)	Supporting studies (A)	Supporting studies (B)
			78.70±21.2 / CG: 78.0±21.6	2024: OG 71(67,73) / GG 75(70,77) / CG 73(67,76)			Arnau 2020: 13 weeks	12 weeks	Arnau 2020: No	Maintain current physical activity	Arnau 2020	
WP vs ω-3+Leu+PS23	WP	ω-3+Leu +PS23	Björkman 2020: WP 83.60±4.7 / P 84.0±3.9 / CG 83.70±5.1; Park 2018: WP 76.80±3.70 / P 77.30±3.67 / CG 76.83±3.86	IG: 78.84±5.80 / CG: 80.50±3.74	Confirmed: 1; At risk: 1	Confirmed	Björkman 2020: 48 weeks; Park 2018: 12 weeks	Rondanelli 2022: 8 weeks	Björkman 2020: Low-intensity home workouts; Park 2018: No	Rondanelli 2022: Basic exercise 5 times/week, 20–30 min	Björkman 2020; Park 2018	Rondanelli 2022
WP vs WP+VD+VE	WP	WP+VD+VE	Björkman 2020: WP 83.60±4.7 / P 84.0±3.9 / CG 83.70±5.1; Park 2018: WP 76.80±3.70 / P 77.30±3.67 / CG 76.83±3.86	IG: 73.23±6.52 / CG: 74.83±5.94	Confirmed: 1; At risk: 1	Confirmed	Björkman 2020: 48 weeks; Park 2018: 12 weeks	Bo 2019: 24 weeks	Björkman 2020: Low-intensity home workouts; Park 2018: No	Bo 2019: No	Björkman 2020; Park 2018	Bo 2019

Indirect comparison	Node A	Node B	Age (A)	Age (B)	Sarcopenia status (A)	Sarcopenia status (B)	Intervention duration (A)	Intervention duration (B)	Background exercise (A)	Background exercise (B)	Supporting studies (A)	Supporting studies (B)
WP vs VD	WP	VD	Björkman 2020: WP 83.60±4.7 / P 84.0±3.9 / CG 83.70±5.1; Park 2018: WP 76.80±3.70 / P 77.30±3.67 / CG 76.83±3.86	IG: 73.05±1.95 / CG: 73.56±2.14	Confirmed: 1; At risk: 1	At risk	Björkman 2020: 48 weeks; Park 2018: 12 weeks	El Hajj 2018: 24 weeks	Björkman 2020: Low-intensity home workouts; Park 2018: No	El Hajj 2018: No	Björkman 2020; Park 2018	El Hajj 2018
WP vs P+Ca+CaP	WP	P+Ca+CaP	Björkman 2020: WP 83.60±4.7 / P 84.0±3.9 / CG 83.70±5.1; Park 2018: WP 76.80±3.70 / P 77.30±3.67 / CG 76.83±3.86	Chen 2024: OG 71(67,73) / GG 75(70,77) / CG 73(67,76)	Confirmed: 1; At risk: 1	At risk	Björkman 2020: 48 weeks; Park 2018: 12 weeks	Chen 2024: 12 weeks	Björkman 2020: Low-intensity home workouts; Park 2018: No	Chen 2024: Maintain current physical activity	Björkman 2020; Park 2018	Chen 2024
WP vs WP+Leu	WP	WP+Leu	Björkman 2020: WP 83.60±4.7 / P 84.0±3.9 / CG 83.70±5.1;	Murphy 2021: LEU-PRO+n-3 73±6 / LEU-PRO	Confirmed: 1; At risk: 1	At risk: 1; Confirmed: 1	Björkman 2020: 48 weeks; Park 2018: 12 weeks	Murphy 2021: 24 weeks; Chung 2026: 12 weeks	Björkman 2020: Low-intensity home workouts; Park 2018:	Murphy 2021: No; Chung 2026: No	Björkman 2020; Park 2018	Murphy 2021; Chung 2026

Indirect comparison	Node A	Node B	Age (A)	Age (B)	Sarcopenia status (A)	Sarcopenia status (B)	Intervention duration (A)	Intervention duration (B)	Background exercise (A)	Background exercise (B)	Supporting studies (A)	Supporting studies (B)
			Park 2018: WP 76.80±3.70 / P 77.30±3.67 / CG 76.83±3.86	70±5 / CG 73±7; Chung 2026: IG 68.80±6.5 / CG 69.70±7.9					No			
WP vs WP+Leu+LCT	WP	WP+Leu+LCT	Björkman 2020: WP 83.60±4.7 / P 84.0±3.9 / CG 83.70±5.1; Park 2018: WP 76.80±3.70 / P 77.30±3.67 / CG 76.83±3.86	Murphy 2021: LEU-PRO+n-3 73±6 / LEU-PRO 70±5 / CG 73±7	Confirmed: 1; At risk: 1	At risk	Björkman 2020: 48 weeks; Park 2018: 12 weeks	Murphy 2021: 24 weeks	Björkman 2020: Low-intensity home workouts; Park 2018: No	Murphy 2021: No	Björkman 2020; Park 2018	Murphy 2021
WP vs Leu+VD+MCT	WP	Leu+VD+MCT	Björkman 2020: WP 83.60±4.7 / P 84.0±3.9 / CG 83.70±5.1; Park 2018: WP 76.80±3.70 / P 77.30±3.67 / CG	Abe 2016: LD+MCT 85.50±3.7 / LD+LCT 87.70±3.9 / CG 86.80±6.5	Confirmed: 1; At risk: 1	At risk	Björkman 2020: 48 weeks; Park 2018: 12 weeks	Abe 2016: 12 weeks	Björkman 2020: Low-intensity home workouts; Park 2018: No	Abe 2016: Regular exercise activities in nursing homes	Björkman 2020; Park 2018	Abe 2016

Indirect comparison	Node A	Node B	Age (A)	Age (B)	Sarcopenia status (A)	Sarcopenia status (B)	Intervention duration (A)	Intervention duration (B)	Background exercise (A)	Background exercise (B)	Supporting studies (A)	Supporting studies (B)
			76.83±3.86									
WP vs Leu+VD+LCT	WP	Leu+VD+LCT	Björkman 2020: WP 83.60±4.7 / P 84.0±3.9 / CG 83.70±5.1; Park 2018: WP 76.80±3.70 / P 77.30±3.67 / CG 76.83±3.86	Abe 2016: LD+MCT 85.50±3.7 / LD+LCT 87.70±3.9 / CG 86.80±6.5	Confirmed: 1; At risk: 1	At risk	Björkman 2020: 48 weeks; Park 2018: 12 weeks	Abe 2016: 12 weeks	Björkman 2020: Low-intensity home workouts; Park 2018: No	Abe 2016: Regular exercise activities in nursing homes	Björkman 2020; Park 2018	Abe 2016
WP vs HMB+ARG+LYS	WP	HMB+ARG+LYS	Björkman 2020: WP 83.60±4.7 / P 84.0±3.9 / CG 83.70±5.1; Park 2018: WP 76.80±3.70 / P 77.30±3.67 / CG 76.83±3.86	IG: 71.50±1.5 / CG: 71.50±1.5	Confirmed: 1; At risk: 1	At risk	Björkman 2020: 48 weeks; Park 2018: 12 weeks	Flakoll 2004: 12 weeks	Björkman 2020: Low-intensity home workouts; Park 2018: No	Flakoll 2004: No	Björkman 2020; Park 2018	Flakoll 2004
WP vs Ca+HMB	WP	Ca+HMB	Björkman 2020: WP 83.60±4.7 / P 84.0±3.9 / CG	IG: 71.50±4.5 / CG: 71.60±4.2	Confirmed: 1; At risk: 1	Confirmed	Björkman 2020: 48 weeks; Park 2018: 12 weeks	Osuka 2021: 12 weeks	Björkman 2020: Low-intensity home workouts;	Osuka 2021: No	Björkman 2020; Park 2018	Osuka 2021

Indirect comparison	Node A	Node B	Age (A)	Age (B)	Sarcopenia status (A)	Sarcopenia status (B)	Intervention duration (A)	Intervention duration (B)	Background exercise (A)	Background exercise (B)	Supporting studies (A)	Supporting studies (B)
			83.70±5.1; Park 2018: WP 76.80±3.70 / P 77.30±3.67 / CG 76.83±3.86						Park 2018: No			
WP vs P+Ca	WP	P+Ca	Björkman 2020: WP 83.60±4.7 / P 84.0±3.9 / CG 83.70±5.1; Park 2018: WP 76.80±3.70 / P 77.30±3.67 / CG 76.83±3.86	Chen 2024: OG 71(67,73) / GG 75(70,77) / CG 73(67,76)	Confirmed: 1; At risk: 1	At risk	Björkman 2020: 48 weeks; Park 2018: 12 weeks	Chen 2024: 12 weeks	Björkman 2020: Low-intensity home workouts; Park 2018: No	Chen 2024: Maintain current physical activity	Björkman 2020; Park 2018	Chen 2024
WP+VD+Leu vs ω-3+Leu+PS23	WP+VD+Leu	ω-3+Leu +PS23	Lin 2021: IG 72.50±5.57 / CG 73.80±8.11; Bauer 2015: IG 77.30±6.7 / CG 78.10±7.0; Rondanelli	IG: 78.84±5.80 / CG: 80.50±3.74	Confirmed (4 studies)	Confirmed	Lin 2021: 12 weeks; Bauer 2015: 13 weeks; Rondanelli 2020: 6 weeks; Rondanelli 2016: 12 weeks	Rondanelli 2022: 8 weeks	Lin 2021: No; Bauer 2015: No; Rondanelli 2020: Personalized physical rehabilitation therapy 5 times/week, 20–30	Rondanelli 2022: Basic exercise 5 times/week, 20–30 min	Lin 2021; Bauer 2015; Rondanelli 2020; Rondanelli 2016	Rondanelli 2022

Indirect comparison	Node A	Node B	Age (A)	Age (B)	Sarcopenia status (A)	Sarcopenia status (B)	Intervention duration (A)	Intervention duration (B)	Background exercise (A)	Background exercise (B)	Supporting studies (A)	Supporting studies (B)
			2020: IG 81±7 / CG 82±5; Rondanelli 2016: IG 80.77±6.29 / CG 80.21±8.54						min/session; Rondanelli 2016: Structured training 5 times/week, 20 min/session			
WP+VD+Leu vs WP+VD+VE	WP+VD+Leu	WP+VD+VE	Lin 2021: IG 72.50±5.57 / CG 73.80±8.11; Bauer 2015: IG 77.30±6.7 / CG 78.10±7.0; Rondanelli 2020: IG 81±7 / CG 82±5; Rondanelli 2016: IG 80.77±6.29 / CG 80.21±8.54	IG: 73.23±6.52 / CG: 74.83±5.94	Confirmed (4 studies)	Confirmed	Lin 2021: 12 weeks; Bauer 2015: 13 weeks; Rondanelli 2020: 6 weeks; Rondanelli 2016: 12 weeks	Bo 2019: 24 weeks	Lin 2021: No; Bauer 2015: No; Rondanelli 2020: Personalized physical rehabilitation therapy 5 times/week, 20–30 min/session; Rondanelli 2016: Structured training 5 times/week, 20 min/session	Bo 2019: No	Lin 2021; Bauer 2015; Rondanelli 2020; Rondanelli 2016	Bo 2019
WP+VD+Leu vs VD	WP+VD+Leu	VD	Lin 2021: IG 72.50±5.57 / CG 73.80±8.11;	IG: 73.05±1.95 / CG: 73.56±2.14	Confirmed (4 studies)	At risk	Lin 2021: 12 weeks; Bauer 2015: 13 weeks; Rondanelli	El Hajj 2018: 24 weeks	Lin 2021: No; Bauer 2015: No; Rondanelli 2020:	El Hajj 2018: No	Lin 2021; Bauer 2015; Rondanelli 2020; Rondanelli	El Hajj 2018

Indirect comparison	Node A	Node B	Age (A)	Age (B)	Sarcopenia status (A)	Sarcopenia status (B)	Intervention duration (A)	Intervention duration (B)	Background exercise (A)	Background exercise (B)	Supporting studies (A)	Supporting studies (B)
			Bauer 2015: IG 77.30±6.7 / CG 78.10±7.0; Rondanelli 2020: IG 81±7 / CG 82±5; Rondanelli 2016: IG 80.77±6.29 / CG 80.21±8.54				2020: 6 weeks; Rondanelli 2016: 12 weeks		Personalized physical rehabilitation therapy 5 times/week, 20–30 min/session; Rondanelli 2016: Structured training 5 times/week, 20 min/session		2016	
WP+VD+Leu vs P+Ca+CaP	WP+VD+Leu	P+Ca+CaP	Lin 2021: IG 72.50±5.57 / CG 73.80±8.11; Bauer 2015: IG 77.30±6.7 / CG 78.10±7.0; Rondanelli 2020: IG 81±7 / CG 82±5; Rondanelli 2016: IG 80.77±6.29 / CG	Chen 2024: OG 71(67,73) / GG 75(70,77) / CG 73(67,76)	Confirmed (4 studies)	At risk	Lin 2021: 12 weeks; Bauer 2015: 13 weeks; Rondanelli 2020: 6 weeks; Rondanelli 2016: 12 weeks	Chen 2024: 12 weeks	Lin 2021: No; Bauer 2015: No; Rondanelli 2020: Personalized physical rehabilitation therapy 5 times/week, 20–30 min/session; Rondanelli 2016: Structured training 5 times/week, 20	Chen 2024: Maintain current physical activity	Lin 2021; Bauer 2015; Rondanelli 2020; Rondanelli 2016	Chen 2024

Indirect comparison	Node A	Node B	Age (A)	Age (B)	Sarcopenia status (A)	Sarcopenia status (B)	Intervention duration (A)	Intervention duration (B)	Background exercise (A)	Background exercise (B)	Supporting studies (A)	Supporting studies (B)
			80.21±8.54						min/session			
WP+VD+Leu vs WP+Leu	WP+VD+Leu	WP+Leu	Lin 2021: IG 72.50±5.57 / CG 73.80±8.11; Bauer 2015: IG 77.30±6.7 / CG 78.10±7.0; Rondanelli 2020: IG 81±7 / CG 82±5; Rondanelli 2016: IG 80.77±6.29 / CG 80.21±8.54	Murphy 2021: LEU-PRO+n-3 73±6 / LEU-PRO 70±5 / CG 73±7; Chung 2026: IG 68.80±6.5 / CG 69.70±7.9	Confirmed (4 studies)	At risk: 1; Confirmed: 1	Lin 2021: 12 weeks; Bauer 2015: 13 weeks; Rondanelli 2020: 6 weeks; Rondanelli 2016: 12 weeks	Murphy 2021: 24 weeks; Chung 2026: 12 weeks	Lin 2021: No; Bauer 2015: No; Rondanelli 2020: Personalized physical rehabilitation therapy 5 times/week, 20–30 min/session; Rondanelli 2016: Structured training 5 times/week, 20 min/session	Murphy 2021: No; Chung 2026: No	Lin 2021; Bauer 2015; Rondanelli 2020; Rondanelli 2016	Murphy 2021; Chung 2026
WP+VD+Leu vs WP+Leu+LCT	WP+VD+Leu	WP+Leu+LCT	Lin 2021: IG 72.50±5.57 / CG 73.80±8.11; Bauer 2015: IG 77.30±6.7 / CG 78.10±7.0; Rondanelli 2020: IG	Murphy 2021: LEU-PRO+n-3 73±6 / LEU-PRO 70±5 / CG 73±7	Confirmed (4 studies)	At risk	Lin 2021: 12 weeks; Bauer 2015: 13 weeks; Rondanelli 2020: 6 weeks; Rondanelli 2016: 12 weeks	Murphy 2021: 24 weeks	Lin 2021: No; Bauer 2015: No; Rondanelli 2020: Personalized physical rehabilitation therapy 5 times/week, 20–30 min/session;	Murphy 2021: No	Lin 2021; Bauer 2015; Rondanelli 2020; Rondanelli 2016	Murphy 2021

Indirect comparison	Node A	Node B	Age (A)	Age (B)	Sarcopenia status (A)	Sarcopenia status (B)	Intervention duration (A)	Intervention duration (B)	Background exercise (A)	Background exercise (B)	Supporting studies (A)	Supporting studies (B)
WP+VD+Leu vs Leu+VD+MCT	WP+VD+Leu	Leu+VD+MCT	81±7 / CG 82±5; Rondanelli 2016: IG 80.77±6.29 / CG 80.21±8.54 Lin 2021: IG 72.50±5.57 / CG 73.80±8.11; Bauer 2015: IG 77.30±6.7 / CG 78.10±7.0; Rondanelli 2020: IG 81±7 / CG 82±5; Rondanelli 2016: IG 80.77±6.29 / CG 80.21±8.54	Abe 2016: LD+MCT 85.50±3.7 / LD+LCT 87.70±3.9 / CG 86.80±6.5	Confirmed (4 studies)	At risk	Lin 2021: 12 weeks; Bauer 2015: 13 weeks; Rondanelli 2020: 6 weeks; Rondanelli 2016: 12 weeks	Abe 2016: 12 weeks	Rondanelli 2016: Structured training 5 times/week, 20 min/session Lin 2021: No; Bauer 2015: No; Rondanelli 2020: Personalized physical rehabilitation therapy 5 times/week, 20–30 min/session; Rondanelli 2016: Structured training 5 times/week, 20 min/session	Abe 2016: Regular exercise activities in nursing homes	Lin 2021; Bauer 2015; Rondanelli 2020; Rondanelli 2016	Abe 2016
WP+VD+Leu vs Leu+VD+LCT	WP+VD+Leu	Leu+VD+LCT	Lin 2021: IG 72.50±5.57 / CG 73.80±8.11; Bauer 2015:	Abe 2016: LD+MCT 85.50±3.7 / LD+LCT 87.70±3.9 / CG	Confirmed (4 studies)	At risk	Lin 2021: 12 weeks; Bauer 2015: 13 weeks; Rondanelli 2020: 6	Abe 2016: 12 weeks	Lin 2021: No; Bauer 2015: No; Rondanelli 2020: Personalized	Abe 2016: Regular exercise activities in nursing homes	Lin 2021; Bauer 2015; Rondanelli 2020; Rondanelli 2016	Abe 2016

Indirect comparison	Node A	Node B	Age (A)	Age (B)	Sarcopenia status (A)	Sarcopenia status (B)	Intervention duration (A)	Intervention duration (B)	Background exercise (A)	Background exercise (B)	Supporting studies (A)	Supporting studies (B)
			IG 77.30±6.7 / CG 78.10±7.0; Rondanelli 2020: IG 81±7 / CG 82±5; Rondanelli 2016: IG 80.77±6.29 / CG 80.21±8.54	86.80±6.5			weeks; Rondanelli 2016: 12 weeks		physical rehabilitation therapy 5 times/week, 20–30 min/session; Rondanelli 2016: Structured training 5 times/week, 20 min/session			
WP+VD+Leu vs HMB+ARG+LYS	WP+VD+Leu	HMB+ARG+LYS	Lin 2021: IG 72.50±5.57 / CG 73.80±8.11; Bauer 2015: IG 77.30±6.7 / CG 78.10±7.0; Rondanelli 2020: IG 81±7 / CG 82±5; Rondanelli 2016: IG 80.77±6.29 / CG 80.21±8.54	IG: 71.50±1.5 / CG: 71.50±1.5	Confirmed (4 studies)	At risk	Lin 2021: 12 weeks; Bauer 2015: 13 weeks; Rondanelli 2020: 6 weeks; Rondanelli 2016: 12 weeks	Flakoll 2004: 12 weeks	Lin 2021: No; Bauer 2015: No; Rondanelli 2020: Personalized physical rehabilitation therapy 5 times/week, 20–30 min/session; Rondanelli 2016: Structured training 5 times/week, 20 min/session	Flakoll 2004: No	Lin 2021; Bauer 2015; Rondanelli 2020; Rondanelli 2016	Flakoll 2004

Indirect comparison	Node A	Node B	Age (A)	Age (B)	Sarcopenia status (A)	Sarcopenia status (B)	Intervention duration (A)	Intervention duration (B)	Background exercise (A)	Background exercise (B)	Supporting studies (A)	Supporting studies (B)
WP+VD+Leu vs Ca+HMB	WP+VD+Leu	Ca+HMB	Lin 2021: IG 72.50±5.57 / CG 73.80±8.11; Bauer 2015: IG 77.30±6.7 / CG 78.10±7.0; Rondanelli 2020: IG 81±7 / CG 82±5; Rondanelli 2016: IG 80.77±6.29 / CG 80.21±8.54	IG: 71.50±4.5 / CG: 71.60±4.2	Confirmed (4 studies)	Confirmed	Lin 2021: 12 weeks; Bauer 2015: 13 weeks; Rondanelli 2020: 6 weeks; Rondanelli 2016: 12 weeks	Osuka 2021: 12 weeks	Lin 2021: No; Bauer 2015: No; Rondanelli 2020: Personalized physical rehabilitation therapy 5 times/week, 20–30 min/session; Rondanelli 2016: Structured training 5 times/week, 20 min/session	Osuka 2021: No	Lin 2021; Bauer 2015; Rondanelli 2020; Rondanelli 2016	Osuka 2021
WP+VD+Leu vs P	WP+VD+Leu	P	Lin 2021: IG 72.50±5.57 / CG 73.80±8.11; Bauer 2015: IG 77.30±6.7 / CG 78.10±7.0; Rondanelli 2020: IG 81±7 / CG	Björkman 2020: WP 83.60±4.7 / P 84.0±3.9 / CG 83.70±5.1; Ottestad 2017: IG 76.80±6.2 / CG 77.10±4.7; Alemán-Mateo	Confirmed (4 studies)	Confirmed: 2; At risk: 3	Lin 2021: 12 weeks; Bauer 2015: 13 weeks; Rondanelli 2020: 6 weeks; Rondanelli 2016: 12 weeks	Björkman 2020: 48 weeks; Ottestad 2017: 12 weeks; Alemán-Mateo 2012: 12 weeks; Kao 2025: 12 weeks; Park 2018: 12 weeks	Lin 2021: No; Bauer 2015: No; Rondanelli 2020: Personalized physical rehabilitation therapy 5 times/week, 20–30 min/session; Rondanelli	Björkman 2020: Low-intensity home workouts; Ottestad 2017: No; Alemán-Mateo 2012: No; Kao 2025: No; Park 2018: No	Lin 2021; Bauer 2015; Rondanelli 2020; Rondanelli 2016	Björkman 2020; Ottestad 2017; Alemán-Mateo 2012; Kao 2025; Park 2018

Indirect comparison	Node A	Node B	Age (A)	Age (B)	Sarcopenia status (A)	Sarcopenia status (B)	Intervention duration (A)	Intervention duration (B)	Background exercise (A)	Background exercise (B)	Supporting studies (A)	Supporting studies (B)
			82±5; Rondanelli 2016: IG 80.77±6.29 / CG 80.21±8.54	2012: IG 75.40±5.0 / CG 76.70±5.8; Kao 2025: IG 72.60±5.1 / CG 72.30±5.2; Park 2018: WP 76.80±3.70 / P 77.30±3.67 / CG 76.83±3.86					2016: Structured training 5 times/week, 20 min/session			
WP+VD+Leu vs P+Ca	WP+VD+Leu	P+Ca	Lin 2021: IG 72.50±5.57 / CG 73.80±8.11; Bauer 2015: IG 77.30±6.7 / CG 78.10±7.0; Rondanelli 2020: IG 81±7 / CG 82±5; Rondanelli 2016: IG	Chen 2024: OG 71(67,73) / GG 75(70,77) / CG 73(67,76)	Confirmed (4 studies)	At risk	Lin 2021: 12 weeks; Bauer 2015: 13 weeks; Rondanelli 2020: 6 weeks; Rondanelli 2016: 12 weeks	Chen 2024: 12 weeks	Lin 2021: No; Bauer 2015: No; Rondanelli 2020: Personalized physical rehabilitation therapy 5 times/week, 20–30 min/session; Rondanelli 2016: Structured training 5	Chen 2024: Maintain current physical activity	Lin 2021; Bauer 2015; Rondanelli 2020; Rondanelli 2016	Chen 2024

Indirect comparison	Node A	Node B	Age (A)	Age (B)	Sarcopenia status (A)	Sarcopenia status (B)	Intervention duration (A)	Intervention duration (B)	Background exercise (A)	Background exercise (B)	Supporting studies (A)	Supporting studies (B)
			80.77±6.29 / CG 80.21±8.54						times/week, 20 min/session			
ω-3+Leu+PS23 vs WP+VD+VE	ω-3+Leu +PS23	WP+VD+VE	IG: 78.84±5.80 / CG: 80.50±3.74	IG: 73.23±6.52 / CG: 74.83±5.94	Confirmed	Confirmed	Rondanelli 2022: 8 weeks	Bo 2019: 24 weeks	Rondanelli 2022: Basic exercise 5 times/week, 20–30 min	Bo 2019: No	Rondanelli 2022	Bo 2019
ω-3+Leu+PS23 vs VD	ω-3+Leu +PS23	VD	IG: 78.84±5.80 / CG: 80.50±3.74	IG: 73.05±1.95 / CG: 73.56±2.14	Confirmed	At risk	Rondanelli 2022: 8 weeks	El Hajj 2018: 24 weeks	Rondanelli 2022: Basic exercise 5 times/week, 20–30 min	El Hajj 2018: No	Rondanelli 2022	El Hajj 2018
ω-3+Leu+PS23 vs P+Ca+CaP	ω-3+Leu +PS23	P+Ca+CaP	IG: 78.84±5.80 / CG: 80.50±3.74	Chen 2024: OG 71(67,73) / GG 75(70,77) / CG 73(67,76)	Confirmed	At risk	Rondanelli 2022: 8 weeks	Chen 2024: 12 weeks	Rondanelli 2022: Basic exercise 5 times/week, 20–30 min	Chen 2024: Maintain current physical activity	Rondanelli 2022	Chen 2024
ω-3+Leu+PS23 vs WP+Leu	ω-3+Leu +PS23	WP+Leu	IG: 78.84±5.80 / CG: 80.50±3.74	Murphy 2021: LEU-PRO+n-3 73±6 / LEU-PRO 70±5 / CG 73±7; Chung 2026: IG 68.80±6.5 / CG	Confirmed	At risk: 1; Confirmed: 1	Rondanelli 2022: 8 weeks	Murphy 2021: 24 weeks; Chung 2026: 12 weeks	Rondanelli 2022: Basic exercise 5 times/week, 20–30 min	Murphy 2021: No; Chung 2026: No	Rondanelli 2022	Murphy 2021; Chung 2026

Indirect comparison	Node A	Node B	Age (A)	Age (B)	Sarcopenia status (A)	Sarcopenia status (B)	Intervention duration (A)	Intervention duration (B)	Background exercise (A)	Background exercise (B)	Supporting studies (A)	Supporting studies (B)
ω-3+Leu+PS23 vs WP+Leu+LCT	ω-3+Leu+PS23	WP+Leu+LCT	IG: 78.84±5.80 / CG: 80.50±3.74	69.70±7.9 Murphy 2021: LEU-PRO+n-3 73±6 / LEU-PRO 70±5 / CG 73±7	Confirmed	At risk	Rondanelli 2022: 8 weeks	Murphy 2021: 24 weeks	Rondanelli 2022: Basic exercise 5 times/week, 20–30 min	Murphy 2021: No	Rondanelli 2022	Murphy 2021
ω-3+Leu+PS23 vs Leu+VD+MCT	ω-3+Leu+PS23	Leu+VD+MCT	IG: 78.84±5.80 / CG: 80.50±3.74	Abe 2016: LD+MCT 85.50±3.7 / LD+LCT 87.70±3.9 / CG 86.80±6.5	Confirmed	At risk	Rondanelli 2022: 8 weeks	Abe 2016: 12 weeks	Rondanelli 2022: Basic exercise 5 times/week, 20–30 min	Abe 2016: Regular exercise activities in nursing homes	Rondanelli 2022	Abe 2016
ω-3+Leu+PS23 vs Leu+VD+LCT	ω-3+Leu+PS23	Leu+VD+LCT	IG: 78.84±5.80 / CG: 80.50±3.74	Abe 2016: LD+MCT 85.50±3.7 / LD+LCT 87.70±3.9 / CG 86.80±6.5	Confirmed	At risk	Rondanelli 2022: 8 weeks	Abe 2016: 12 weeks	Rondanelli 2022: Basic exercise 5 times/week, 20–30 min	Abe 2016: Regular exercise activities in nursing homes	Rondanelli 2022	Abe 2016
ω-3+Leu+PS23 vs HMB+ARG+LYS	ω-3+Leu+PS23	HMB+ARG+LYS	IG: 78.84±5.80 / CG: 80.50±3.74	IG: 71.50±1.5 / CG: 71.50±1.5	Confirmed	At risk	Rondanelli 2022: 8 weeks	Flakoll 2004: 12 weeks	Rondanelli 2022: Basic exercise 5 times/week, 20–30 min	Flakoll 2004: No	Rondanelli 2022	Flakoll 2004
ω-3+Leu+PS23 vs Ca+HMB	ω-3+Leu+PS23	Ca+HMB	IG: 78.84±5.80 / CG: 80.50±3.74	IG: 71.50±4.5 / CG: 71.60±4.2	Confirmed	Confirmed	Rondanelli 2022: 8 weeks	Osuka 2021: 12 weeks	Rondanelli 2022: Basic exercise 5 times/week,	Osuka 2021: No	Rondanelli 2022	Osuka 2021

Indirect comparison	Node A	Node B	Age (A)	Age (B)	Sarcopenia status (A)	Sarcopenia status (B)	Intervention duration (A)	Intervention duration (B)	Background exercise (A)	Background exercise (B)	Supporting studies (A)	Supporting studies (B)
									20–30 min			
ω-3+Leu+PS23 vs P	ω-3+Leu +PS23	P	IG: 78.84±5.80 / CG: 80.50±3.74	Björkman 2020: WP 83.60±4.7 / P 84.0±3.9 / CG 83.70±5.1; Ottestad 2017: IG 76.80±6.2 / CG 77.10±4.7; Alemán-Mateo 2012: IG 75.40±5.0 / CG 76.70±5.8; Kao 2025: IG 72.60±5.1 / CG 72.30±5.2; Park 2018: WP 76.80±3.70 / P 77.30±3.67 / CG 76.83±3.86	Confirmed	Confirmed: 2; At risk: 3	Rondanelli 2022: 8 weeks	Björkman 2020: 48 weeks; Ottestad 2017: 12 weeks; Alemán-Mateo 2012: 12 weeks; Kao 2025: 12 weeks; Park 2018: 12 weeks	Rondanelli 2022: Basic exercise 5 times/week, 20–30 min	Björkman 2020: Low-intensity home workouts; Ottestad 2017: No; Alemán-Mateo 2012: No; Kao 2025: No; Park 2018: No	Rondanelli 2022	Björkman 2020; Ottestad 2017; Alemán-Mateo 2012; Kao 2025; Park 2018
ω-3+Leu+PS23	ω-3+Leu	P+Ca	IG: 78.84±5.80 /	Chen 2024: OG	Confirmed	At risk	Rondanelli 2022: 8 weeks	Chen 2024: 12 weeks	Rondanelli 2022: Basic	Chen 2024: Maintain	Rondanelli 2022	Chen 2024

Indirect comparison	Node A	Node B	Age (A)	Age (B)	Sarcopenia status (A)	Sarcopenia status (B)	Intervention duration (A)	Intervention duration (B)	Background exercise (A)	Background exercise (B)	Supporting studies (A)	Supporting studies (B)
vs P+Ca	+PS23		CG: 80.50±3.74	71(67,73) / GG 75(70,77) / CG 73(67,76)					exercise 5 times/week, 20–30 min	current physical activity		
WP+VD+VE vs VD	WP+VD+VE	VD	IG: 73.23±6.52 / CG: 74.83±5.94	IG: 73.05±1.95 / CG: 73.56±2.14	Confirmed	At risk	Bo 2019: 24 weeks	El Hajj 2018: 24 weeks	Bo 2019: No	El Hajj 2018: No	Bo 2019	El Hajj 2018
WP+VD+VE vs P+Ca+CaP	WP+VD+VE	P+Ca+CaP	IG: 73.23±6.52 / CG: 74.83±5.94	Chen 2024: OG 71(67,73) / GG 75(70,77) / CG 73(67,76)	Confirmed	At risk	Bo 2019: 24 weeks	Chen 2024: 12 weeks	Bo 2019: No	Chen 2024: Maintain current physical activity	Bo 2019	Chen 2024
WP+VD+VE vs WP+Leu	WP+VD+VE	WP+Leu	IG: 73.23±6.52 / CG: 74.83±5.94	Murphy 2021: LEU-PRO+n-3 73±6 / LEU-PRO 70±5 / CG 73±7; Chung 2026: IG 68.80±6.5 / CG 69.70±7.9	Confirmed	At risk: 1; Confirmed: 1	Bo 2019: 24 weeks	Murphy 2021: 24 weeks; Chung 2026: 12 weeks	Bo 2019: No	Murphy 2021: No; Chung 2026: No	Bo 2019	Murphy 2021; Chung 2026
WP+VD+VE vs WP+Leu+LC	WP+VD+VE	WP+Leu+LCT	IG: 73.23±6.52 / CG:	Murphy 2021: LEU-	Confirmed	At risk	Bo 2019: 24 weeks	Murphy 2021: 24 weeks	Bo 2019: No	Murphy 2021: No	Bo 2019	Murphy 2021

Indirect comparison	Node A	Node B	Age (A)	Age (B)	Sarcopenia status (A)	Sarcopenia status (B)	Intervention duration (A)	Intervention duration (B)	Background exercise (A)	Background exercise (B)	Supporting studies (A)	Supporting studies (B)
T			74.83±5.94	PRO+n-3 73±6 / LEU-PRO 70±5 / CG 73±7								
WP+VD+VE vs Leu+VD+MCT	WP+VD+VE	Leu+VD+MCT	IG: 73.23±6.52 / CG: 74.83±5.94	Abe 2016: LD+MCT 85.50±3.7 / LD+LCT 87.70±3.9 / CG 86.80±6.5	Confirmed	At risk	Bo 2019: 24 weeks	Abe 2016: 12 weeks	Bo 2019: No	Abe 2016: Regular exercise activities in nursing homes	Bo 2019	Abe 2016
WP+VD+VE vs Leu+VD+LCT	WP+VD+VE	Leu+VD+LCT	IG: 73.23±6.52 / CG: 74.83±5.94	Abe 2016: LD+MCT 85.50±3.7 / LD+LCT 87.70±3.9 / CG 86.80±6.5	Confirmed	At risk	Bo 2019: 24 weeks	Abe 2016: 12 weeks	Bo 2019: No	Abe 2016: Regular exercise activities in nursing homes	Bo 2019	Abe 2016
WP+VD+VE vs HMB+ARG+LYS	WP+VD+VE	HMB+ARG+LYS	IG: 73.23±6.52 / CG: 74.83±5.94	IG: 71.50±1.5 / CG: 71.50±1.5	Confirmed	At risk	Bo 2019: 24 weeks	Flakoll 2004: 12 weeks	Bo 2019: No	Flakoll 2004: No	Bo 2019	Flakoll 2004
WP+VD+VE vs Ca+HMB	WP+VD+VE	Ca+HMB	IG: 73.23±6.52 / CG: 74.83±5.94	IG: 71.50±4.5 / CG: 71.60±4.2	Confirmed	Confirmed	Bo 2019: 24 weeks	Osuka 2021: 12 weeks	Bo 2019: No	Osuka 2021: No	Bo 2019	Osuka 2021
WP+VD+VE vs P	WP+VD+VE	P	IG: 73.23±6.52 / CG: 74.83±5.94	Björkman 2020: WP 83.60±4.7 / P 84.0±3.9 / CG	Confirmed	Confirmed: 2; At risk: 3	Bo 2019: 24 weeks	Björkman 2020: 48 weeks; Ottestad 2017: 12	Bo 2019: No	Björkman 2020: Low-intensity home workouts;	Bo 2019	Björkman 2020; Ottestad 2017; Alemán-

Indirect comparison	Node A	Node B	Age (A)	Age (B)	Sarcopenia status (A)	Sarcopenia status (B)	Intervention duration (A)	Intervention duration (B)	Background exercise (A)	Background exercise (B)	Supporting studies (A)	Supporting studies (B)
				83.70±5.1; Ottestad 2017: IG 76.80±6.2 / CG 77.10±4.7; Alemán-Mateo 2012: IG 75.40±5.0 / CG 76.70±5.8; Kao 2025: IG 72.60±5.1 / CG 72.30±5.2; Park 2018: WP 76.80±3.70 / P 77.30±3.67 / CG 76.83±3.86				weeks; Alemán-Mateo 2012: 12 weeks; Kao 2025: 12 weeks; Park 2018: 12 weeks		Ottestad 2017: No; Alemán-Mateo 2012: No; Kao 2025: No; Park 2018: No		Mateo 2012; Kao 2025; Park 2018
WP+VD+VE vs P+Ca	WP+VD+VE	P+Ca	IG: 73.23±6.52 / CG: 74.83±5.94	Chen 2024: OG 71(67,73) / GG 75(70,77) / CG 73(67,76)	Confirmed	At risk	Bo 2019: 24 weeks	Chen 2024: 12 weeks	Bo 2019: No	Chen 2024: Maintain current physical activity	Bo 2019	Chen 2024
VD vs	VD	P+Ca+	IG:	Chen	At risk	At risk	El Hajj 2018:	Chen 2024:	El Hajj 2018:	Chen 2024:	El Hajj 2018	Chen 2024

Indirect comparison	Node A	Node B	Age (A)	Age (B)	Sarcopenia status (A)	Sarcopenia status (B)	Intervention duration (A)	Intervention duration (B)	Background exercise (A)	Background exercise (B)	Supporting studies (A)	Supporting studies (B)
P+Ca+CaP		CaP	73.05±1.95 / CG: 73.56±2.14	2024: OG 71(67,73) / GG 75(70,77) / CG 73(67,76)			24 weeks	12 weeks	No	Maintain current physical activity		
VD vs WP+Leu	VD	WP+Leu	IG: 73.05±1.95 / CG: 73.56±2.14	Murphy 2021: LEU-PRO+n-3 73±6 / LEU-PRO 70±5 / CG 73±7; Chung 2026: IG 68.80±6.5 / CG 69.70±7.9	At risk	At risk: 1; Confirmed: 1	El Hajj 2018: 24 weeks	Murphy 2021: 24 weeks; Chung 2026: 12 weeks	El Hajj 2018: No	Murphy 2021: No; Chung 2026: No	El Hajj 2018	Murphy 2021; Chung 2026
VD vs WP+Leu+LCT	VD	WP+Leu+LCT	IG: 73.05±1.95 / CG: 73.56±2.14	Murphy 2021: LEU-PRO+n-3 73±6 / LEU-PRO 70±5 / CG 73±7	At risk	At risk	El Hajj 2018: 24 weeks	Murphy 2021: 24 weeks	El Hajj 2018: No	Murphy 2021: No	El Hajj 2018	Murphy 2021
VD vs Leu+VD+MCT	VD	Leu+VD+MCT	IG: 73.05±1.95 / CG: 73.56±2.14	Abe 2016: LD+MCT 85.50±3.7 / LD+LCT 87.70±3.9 /	At risk	At risk	El Hajj 2018: 24 weeks	Abe 2016: 12 weeks	El Hajj 2018: No	Abe 2016: Regular exercise activities in nursing	El Hajj 2018	Abe 2016

Indirect comparison	Node A	Node B	Age (A)	Age (B)	Sarcopenia status (A)	Sarcopenia status (B)	Intervention duration (A)	Intervention duration (B)	Background exercise (A)	Background exercise (B)	Supporting studies (A)	Supporting studies (B)
				CG 86.80±6.5						homes		
VD vs Leu+VD+LCT	VD	Leu+VD+LCT	IG: 73.05±1.95 / CG: 73.56±2.14	Abe 2016: LD+MCT 85.50±3.7 / LD+LCT 87.70±3.9 / CG 86.80±6.5	At risk	At risk	El Hajj 2018: 24 weeks	Abe 2016: 12 weeks	El Hajj 2018: No	Abe 2016: Regular exercise activities in nursing homes	El Hajj 2018	Abe 2016
VD vs HMB+ARG+LYS	VD	HMB+ARG+LYS	IG: 73.05±1.95 / CG: 73.56±2.14	IG: 71.50±1.5 / CG: 71.50±1.5	At risk	At risk	El Hajj 2018: 24 weeks	Flakoll 2004: 12 weeks	El Hajj 2018: No	Flakoll 2004: No	El Hajj 2018	Flakoll 2004
VD vs Ca+HMB	VD	Ca+HMB	IG: 73.05±1.95 / CG: 73.56±2.14	IG: 71.50±4.5 / CG: 71.60±4.2	At risk	Confirmed	El Hajj 2018: 24 weeks	Osuka 2021: 12 weeks	El Hajj 2018: No	Osuka 2021: No	El Hajj 2018	Osuka 2021
VD vs P	VD	P	IG: 73.05±1.95 / CG: 73.56±2.14	Björkman 2020: WP 83.60±4.7 / P 84.0±3.9 / CG 83.70±5.1; Ottestad 2017: IG 76.80±6.2 / CG 77.10±4.7; Alemán-Mateo 2012: IG 75.40±5.0 /	At risk	Confirmed: 2; At risk: 3	El Hajj 2018: 24 weeks	Björkman 2020: 48 weeks; Ottestad 2017: 12 weeks; Alemán-Mateo 2012: 12 weeks; Kao 2025: 12 weeks; Park 2018: 12 weeks	El Hajj 2018: No	Björkman 2020: Low-intensity home workouts; Ottestad 2017: No; Alemán-Mateo 2012: No; Kao 2025: No; Park 2018: No	El Hajj 2018	Björkman 2020; Ottestad 2017; Alemán-Mateo 2012; Kao 2025; Park 2018

Indirect comparison	Node A	Node B	Age (A)	Age (B)	Sarcopenia status (A)	Sarcopenia status (B)	Intervention duration (A)	Intervention duration (B)	Background exercise (A)	Background exercise (B)	Supporting studies (A)	Supporting studies (B)
VD vs P+Ca	VD	P+Ca	IG: 73.05±1.95 / CG: 73.56±2.14	CG 76.70±5.8; Kao 2025: IG 72.60±5.1 / CG 72.30±5.2; Park 2018: WP 76.80±3.70 / P 77.30±3.67 / CG 76.83±3.86 Chen 2024: OG 71(67,73) / GG 75(70,77) / CG 73(67,76)	At risk	At risk	El Hajj 2018: 24 weeks	Chen 2024: 12 weeks	El Hajj 2018: No	Chen 2024: Maintain current physical activity	El Hajj 2018	Chen 2024
P+Ca+CaP vs WP+Leu	P+Ca+ CaP	WP+L eu	Chen 2024: OG 71(67,73) / GG 75(70,77) / CG 73(67,76)	Murphy 2021: LEU-PRO+n-3 73±6 / LEU-PRO 70±5 / CG 73±7; Chung 2026: IG 68.80±6.5 /	At risk	At risk: 1; Confirmed: 1	Chen 2024: 12 weeks	Murphy 2021: 24 weeks; Chung 2026: 12 weeks	Chen 2024: Maintain current physical activity	Murphy 2021: No; Chung 2026: No	Chen 2024	Murphy 2021; Chung 2026

Indirect comparison	Node A	Node B	Age (A)	Age (B)	Sarcopenia status (A)	Sarcopenia status (B)	Intervention duration (A)	Intervention duration (B)	Background exercise (A)	Background exercise (B)	Supporting studies (A)	Supporting studies (B)
P+Ca+CaP vs WP+Leu+LCT	P+Ca+CaP	WP+Leu+LCT	Chen 2024: OG 71(67,73) / GG 75(70,77) / CG 73(67,76)	CG 69.70±7.9 Murphy 2021: LEU-PRO+n-3 73±6 / LEU-PRO 70±5 / CG 73±7	At risk	At risk	Chen 2024: 12 weeks	Murphy 2021: 24 weeks	Chen 2024: Maintain current physical activity	Murphy 2021: No	Chen 2024	Murphy 2021
P+Ca+CaP vs Leu+VD+MCT	P+Ca+CaP	Leu+VD+MCT	Chen 2024: OG 71(67,73) / GG 75(70,77) / CG 73(67,76)	Abe 2016: LD+MCT 85.50±3.7 / LD+LCT 87.70±3.9 / CG 86.80±6.5	At risk	At risk	Chen 2024: 12 weeks	Abe 2016: 12 weeks	Chen 2024: Maintain current physical activity	Abe 2016: Regular exercise activities in nursing homes	Chen 2024	Abe 2016
P+Ca+CaP vs Leu+VD+LCT	P+Ca+CaP	Leu+VD+LCT	Chen 2024: OG 71(67,73) / GG 75(70,77) / CG 73(67,76)	Abe 2016: LD+MCT 85.50±3.7 / LD+LCT 87.70±3.9 / CG 86.80±6.5	At risk	At risk	Chen 2024: 12 weeks	Abe 2016: 12 weeks	Chen 2024: Maintain current physical activity	Abe 2016: Regular exercise activities in nursing homes	Chen 2024	Abe 2016
P+Ca+CaP vs HMB+ARG+LYS	P+Ca+CaP	HMB+ARG+LYS	Chen 2024: OG 71(67,73) / GG 75(70,77) / CG 73(67,76)	IG: 71.50±1.5 / CG: 71.50±1.5	At risk	At risk	Chen 2024: 12 weeks	Flakoll 2004: 12 weeks	Chen 2024: Maintain current physical activity	Flakoll 2004: No	Chen 2024	Flakoll 2004
P+Ca+CaP vs	P+Ca+	Ca+H	Chen 2024:	IG:	At risk	Confirmed	Chen 2024:	Osuka 2021:	Chen 2024:	Osuka 2021:	Chen 2024	Osuka 2021

Indirect comparison	Node A	Node B	Age (A)	Age (B)	Sarcopenia status (A)	Sarcopenia status (B)	Intervention duration (A)	Intervention duration (B)	Background exercise (A)	Background exercise (B)	Supporting studies (A)	Supporting studies (B)
Ca+HMB	CaP	MB	OG 71(67,73) / GG 75(70,77) / CG 73(67,76)	71.50±4.5 / CG: 71.60±4.2			12 weeks	12 weeks	Maintain current physical activity	No		
P+Ca+CaP vs P	P+Ca+ CaP	P	Chen 2024: OG 71(67,73) / GG 75(70,77) / CG 73(67,76)	Björkman 2020: WP 83.60±4.7 / P 84.0±3.9 / CG 83.70±5.1; Ottestad 2017: IG 76.80±6.2 / CG 77.10±4.7; Alemán-Mateo 2012: IG 75.40±5.0 / CG 76.70±5.8; Kao 2025: IG 72.60±5.1 / CG 72.30±5.2; Park 2018: WP 76.80±3.70 / P	At risk	Confirmed: 2; At risk: 3	Chen 2024: 12 weeks	Björkman 2020: 48 weeks; Ottestad 2017: 12 weeks; Alemán-Mateo 2012: 12 weeks; Kao 2025: 12 weeks; Park 2018: 12 weeks	Chen 2024: Maintain current physical activity	Björkman 2020: Low-intensity home workouts; Ottestad 2017: No; Alemán-Mateo 2012: No; Kao 2025: No; Park 2018: No	Chen 2024	Björkman 2020; Ottestad 2017; Alemán-Mateo 2012; Kao 2025; Park 2018

Indirect comparison	Node A	Node B	Age (A)	Age (B)	Sarcopenia status (A)	Sarcopenia status (B)	Intervention duration (A)	Intervention duration (B)	Background exercise (A)	Background exercise (B)	Supporting studies (A)	Supporting studies (B)
				77.30±3.67 / CG 76.83±3.86								
WP+Leu vs Leu+VD+MCT	WP+Leu	Leu+VD+MCT	Murphy 2021: LEU-PRO+n-3 73±6 / LEU-PRO 70±5 / CG 73±7; Chung 2026: IG 68.80±6.5 / CG 69.70±7.9	Abe 2016: LD+MCT 85.50±3.7 / LD+LCT 87.70±3.9 / CG 86.80±6.5	At risk: 1; Confirmed: 1	At risk	Murphy 2021: 24 weeks; Chung 2026: 12 weeks	Abe 2016: 12 weeks	Murphy 2021: No; Chung 2026: No	Abe 2016: Regular exercise activities in nursing homes	Murphy 2021; Chung 2026	Abe 2016
WP+Leu vs Leu+VD+LCT	WP+Leu	Leu+VD+LCT	Murphy 2021: LEU-PRO+n-3 73±6 / LEU-PRO 70±5 / CG 73±7; Chung 2026: IG 68.80±6.5 / CG 69.70±7.9	Abe 2016: LD+MCT 85.50±3.7 / LD+LCT 87.70±3.9 / CG 86.80±6.5	At risk: 1; Confirmed: 1	At risk	Murphy 2021: 24 weeks; Chung 2026: 12 weeks	Abe 2016: 12 weeks	Murphy 2021: No; Chung 2026: No	Abe 2016: Regular exercise activities in nursing homes	Murphy 2021; Chung 2026	Abe 2016
WP+Leu vs HMB+ARG+LYS	WP+Leu	HMB+ARG+LYS	Murphy 2021: LEU-PRO+n-3 73±6 / LEU-PRO 70±5 / CG 73±7; Chung	IG: 71.50±1.5 / CG: 71.50±1.5	At risk: 1; Confirmed: 1	At risk	Murphy 2021: 24 weeks; Chung 2026: 12 weeks	Flakoll 2004: 12 weeks	Murphy 2021: No; Chung 2026: No	Flakoll 2004: No	Murphy 2021; Chung 2026	Flakoll 2004

Indirect comparison	Node A	Node B	Age (A)	Age (B)	Sarcopenia status (A)	Sarcopenia status (B)	Intervention duration (A)	Intervention duration (B)	Background exercise (A)	Background exercise (B)	Supporting studies (A)	Supporting studies (B)
WP+Leu vs Ca+HMB	WP+Leu	Ca+HMB	2026: IG 68.80±6.5 / CG 69.70±7.9 Murphy 2021: LEU-PRO+n-3 73±6 / LEU-PRO 70±5 / CG 73±7; Chung 2026: IG 68.80±6.5 / CG 69.70±7.9	IG: 71.50±4.5 / CG: 71.60±4.2	At risk: 1; Confirmed: 1	Confirmed	Murphy 2021: 24 weeks; Chung 2026: 12 weeks	Osuka 2021: 12 weeks	Murphy 2021: No; Chung 2026: No	Osuka 2021: No	Murphy 2021; Chung 2026	Osuka 2021
WP+Leu vs P	WP+Leu	P	Murphy 2021: LEU-PRO+n-3 73±6 / LEU-PRO 70±5 / CG 73±7; Chung 2026: IG 68.80±6.5 / CG 69.70±7.9	Björkman 2020: WP 83.60±4.7 / P 84.0±3.9 / CG 83.70±5.1; Ottestad 2017: IG 76.80±6.2 / CG 77.10±4.7; Alemán-Mateo 2012: IG 75.40±5.0 / CG 76.70±5.8;	At risk: 1; Confirmed: 1	Confirmed: 2; At risk: 3	Murphy 2021: 24 weeks; Chung 2026: 12 weeks	Björkman 2020: 48 weeks; Ottestad 2017: 12 weeks; Alemán-Mateo 2012: 12 weeks; Kao 2025: 12 weeks; Park 2018: 12 weeks	Murphy 2021: No; Chung 2026: No	Björkman 2020: Low-intensity home workouts; Ottestad 2017: No; Alemán-Mateo 2012: No; Kao 2025: No; Park 2018: No	Murphy 2021; Chung 2026	Björkman 2020; Ottestad 2017; Alemán-Mateo 2012; Kao 2025; Park 2018

Indirect comparison	Node A	Node B	Age (A)	Age (B)	Sarcopenia status (A)	Sarcopenia status (B)	Intervention duration (A)	Intervention duration (B)	Background exercise (A)	Background exercise (B)	Supporting studies (A)	Supporting studies (B)
				Kao 2025: IG 72.60±5.1 / CG 72.30±5.2; Park 2018: WP 76.80±3.70 / P 77.30±3.67 / CG 76.83±3.86								
WP+Leu vs P+Ca	WP+Leu	P+Ca	Murphy 2021: LEU-PRO+n-3 73±6 / LEU-PRO 70±5 / CG 73±7; Chung 2026: IG 68.80±6.5 / CG 69.70±7.9	Chen 2024: OG 71(67,73) / GG 75(70,77) / CG 73(67,76)	At risk: 1; Confirmed: 1	At risk	Murphy 2021: 24 weeks; Chung 2026: 12 weeks	Chen 2024: 12 weeks	Murphy 2021: No; Chung 2026: No	Chen 2024: Maintain current physical activity	Murphy 2021; Chung 2026	Chen 2024
WP+Leu+LCT vs Leu+VD+MCT	WP+Leu+LCT	Leu+VD+MCT	Murphy 2021: LEU-PRO+n-3 73±6 / LEU-PRO 70±5 / CG 73±7	Abe 2016: LD+MCT 85.50±3.7 / LD+LCT 87.70±3.9 / CG 86.80±6.5	At risk	At risk	Murphy 2021: 24 weeks	Abe 2016: 12 weeks	Murphy 2021: No	Abe 2016: Regular exercise activities in nursing homes	Murphy 2021	Abe 2016
WP+Leu+LCT vs	WP+Leu+LC	Leu+VD+LC	Murphy 2021: LEU-	Abe 2016: LD+MCT	At risk	At risk	Murphy 2021: 24	Abe 2016: 12 weeks	Murphy 2021: No	Abe 2016: Regular	Murphy 2021	Abe 2016

Indirect comparison	Node A	Node B	Age (A)	Age (B)	Sarcopenia status (A)	Sarcopenia status (B)	Intervention duration (A)	Intervention duration (B)	Background exercise (A)	Background exercise (B)	Supporting studies (A)	Supporting studies (B)
Leu+VD+LCT	T	T	PRO+n-3 73±6 / LEU-PRO 70±5 / CG 73±7	85.50±3.7 / LD+LCT 87.70±3.9 / CG 86.80±6.5			weeks			exercise activities in nursing homes		
WP+Leu+LCT vs HMB+ARG+LYS	WP+Leu+LCT	HMB+ARG+LYS	Murphy 2021: LEU-PRO+n-3 73±6 / LEU-PRO 70±5 / CG 73±7	IG: 71.50±1.5 / CG: 71.50±1.5	At risk	At risk	Murphy 2021: 24 weeks	Flakoll 2004: 12 weeks	Murphy 2021: No	Flakoll 2004: No	Murphy 2021	Flakoll 2004
WP+Leu+LCT vs Ca+HMB	WP+Leu+LCT	Ca+HMB	Murphy 2021: LEU-PRO+n-3 73±6 / LEU-PRO 70±5 / CG 73±7	IG: 71.50±4.5 / CG: 71.60±4.2	At risk	Confirmed	Murphy 2021: 24 weeks	Osuka 2021: 12 weeks	Murphy 2021: No	Osuka 2021: No	Murphy 2021	Osuka 2021
WP+Leu+LCT vs P	WP+Leu+LCT	P	Murphy 2021: LEU-PRO+n-3 73±6 / LEU-PRO 70±5 / CG 73±7	Björkman 2020: WP 83.60±4.7 / P 84.0±3.9 / CG 83.70±5.1; Ottestad 2017: IG 76.80±6.2 / CG 77.10±4.7; Alemán-Mateo 2012: IG 75.40±5.0 /	At risk	Confirmed: 2; At risk: 3	Murphy 2021: 24 weeks	Björkman 2020: 48 weeks; Ottestad 2017: 12 weeks; Alemán-Mateo 2012: 12 weeks; Kao 2025: 12 weeks; Park 2018: 12 weeks	Murphy 2021: No	Björkman 2020: Low-intensity home workouts; Ottestad 2017: No; Alemán-Mateo 2012: No; Kao 2025: No; Park 2018: No	Murphy 2021	Björkman 2020; Ottestad 2017; Alemán-Mateo 2012; Kao 2025; Park 2018

Indirect comparison	Node A	Node B	Age (A)	Age (B)	Sarcopenia status (A)	Sarcopenia status (B)	Intervention duration (A)	Intervention duration (B)	Background exercise (A)	Background exercise (B)	Supporting studies (A)	Supporting studies (B)
				CG 76.70±5.8; Kao 2025: IG 72.60±5.1 / CG 72.30±5.2; Park 2018: WP 76.80±3.70 / P 77.30±3.67 / CG 76.83±3.86								
WP+Leu+LCT vs P+Ca	WP+Leu+LCT	P+Ca	Murphy 2021: LEU-PRO+n-3 73±6 / LEU-PRO 70±5 / CG 73±7	Chen 2024: OG 71(67,73) / GG 75(70,77) / CG 73(67,76)	At risk	At risk	Murphy 2021: 24 weeks	Chen 2024: 12 weeks	Murphy 2021: No	Chen 2024: Maintain current physical activity	Murphy 2021	Chen 2024
Leu+VD+MCT vs HMB+ARG+LYS	Leu+VD+MCT	HMB+ARG+LYS	Abe 2016: LD+MCT 85.50±3.7 / LD+LCT 87.70±3.9 / CG 86.80±6.5	IG: 71.50±1.5 / CG: 71.50±1.5	At risk	At risk	Abe 2016: 12 weeks	Flakoll 2004: 12 weeks	Abe 2016: Regular exercise activities in nursing homes	Flakoll 2004: No	Abe 2016	Flakoll 2004
Leu+VD+MCT vs Ca+HMB	Leu+VD+MCT	Ca+HMB	Abe 2016: LD+MCT 85.50±3.7 / LD+LCT	IG: 71.50±4.5 / CG: 71.60±4.2	At risk	Confirmed	Abe 2016: 12 weeks	Osuka 2021: 12 weeks	Abe 2016: Regular exercise activities in	Osuka 2021: No	Abe 2016	Osuka 2021

Indirect comparison	Node A	Node B	Age (A)	Age (B)	Sarcopenia status (A)	Sarcopenia status (B)	Intervention duration (A)	Intervention duration (B)	Background exercise (A)	Background exercise (B)	Supporting studies (A)	Supporting studies (B)
			87.70±3.9 / CG 86.80±6.5						nursing homes			
Leu+VD+MCT vs P	Leu+VD+MCT	P	Abe 2016: LD+MCT 85.50±3.7 / LD+LCT 87.70±3.9 / CG 86.80±6.5	Björkman 2020: WP 83.60±4.7 / P 84.0±3.9 / CG 83.70±5.1; Ottestad 2017: IG 76.80±6.2 / CG 77.10±4.7; Alemán-Mateo 2012: IG 75.40±5.0 / CG 76.70±5.8; Kao 2025: IG 72.60±5.1 / CG 72.30±5.2; Park 2018: WP 76.80±3.70 / P 77.30±3.67 / CG 76.83±3.86	At risk	Confirmed: 2; At risk: 3	Abe 2016: 12 weeks	Björkman 2020: 48 weeks; Ottestad 2017: 12 weeks; Alemán-Mateo 2012: 12 weeks; Kao 2025: 12 weeks; Park 2018: 12 weeks	Abe 2016: Regular exercise activities in nursing homes	Björkman 2020: Low-intensity home workouts; Ottestad 2017: No; Alemán-Mateo 2012: No; Kao 2025: No; Park 2018: No	Abe 2016	Björkman 2020; Ottestad 2017; Alemán-Mateo 2012; Kao 2025; Park 2018

Indirect comparison	Node A	Node B	Age (A)	Age (B)	Sarcopenia status (A)	Sarcopenia status (B)	Intervention duration (A)	Intervention duration (B)	Background exercise (A)	Background exercise (B)	Supporting studies (A)	Supporting studies (B)
Leu+VD+MCT vs P+Ca	Leu+VD+MCT	P+Ca	Abe 2016: LD+MCT 85.50±3.7 / LD+LCT 87.70±3.9 / CG 86.80±6.5	Chen 2024: OG 71(67,73) / GG 75(70,77) / CG 73(67,76)	At risk	At risk	Abe 2016: 12 weeks	Chen 2024: 12 weeks	Abe 2016: Regular exercise activities in nursing homes	Chen 2024: Maintain current physical activity	Abe 2016	Chen 2024
Leu+VD+LCT vs HMB+ARG+LYS	Leu+VD+LCT	HMB+ARG+LYS	Abe 2016: LD+MCT 85.50±3.7 / LD+LCT 87.70±3.9 / CG 86.80±6.5	IG: 71.50±1.5 / CG: 71.50±1.5	At risk	At risk	Abe 2016: 12 weeks	Flakoll 2004: 12 weeks	Abe 2016: Regular exercise activities in nursing homes	Flakoll 2004: No	Abe 2016	Flakoll 2004
Leu+VD+LCT vs Ca+HMB	Leu+VD+LCT	Ca+HMB	Abe 2016: LD+MCT 85.50±3.7 / LD+LCT 87.70±3.9 / CG 86.80±6.5	IG: 71.50±4.5 / CG: 71.60±4.2	At risk	Confirmed	Abe 2016: 12 weeks	Osuka 2021: 12 weeks	Abe 2016: Regular exercise activities in nursing homes	Osuka 2021: No	Abe 2016	Osuka 2021
Leu+VD+LCT vs P	Leu+VD+LCT	P	Abe 2016: LD+MCT 85.50±3.7 / LD+LCT 87.70±3.9 / CG 86.80±6.5	Björkman 2020: WP 83.60±4.7 / P 84.0±3.9 / CG 83.70±5.1; Ottestad 2017: IG 76.80±6.2 / CG 77.10±4.7;	At risk	Confirmed: 2; At risk: 3	Abe 2016: 12 weeks	Björkman 2020: 48 weeks; Ottestad 2017: 12 weeks; Alemán-Mateo 2012: 12 weeks; Kao 2025: 12 weeks; Park	Abe 2016: Regular exercise activities in nursing homes	Björkman 2020: Low-intensity home workouts; Ottestad 2017: No; Alemán-Mateo 2012: No; Kao 2025: No;	Abe 2016	Björkman 2020; Ottestad 2017; Alemán-Mateo 2012; Kao 2025; Park 2018

Indirect comparison	Node A	Node B	Age (A)	Age (B)	Sarcopenia status (A)	Sarcopenia status (B)	Intervention duration (A)	Intervention duration (B)	Background exercise (A)	Background exercise (B)	Supporting studies (A)	Supporting studies (B)
				Alemán-Mateo 2012: IG 75.40±5.0 / CG 76.70±5.8; Kao 2025: IG 72.60±5.1 / CG 72.30±5.2; Park 2018: WP 76.80±3.70 / P 77.30±3.67 / CG 76.83±3.86				2018: 12 weeks		Park 2018: No		
Leu+VD+LCT vs P+Ca	Leu+VD+LCT	P+Ca	Abe 2016: LD+MCT 85.50±3.7 / LD+LCT 87.70±3.9 / CG 86.80±6.5	Chen 2024: OG 71(67,73) / GG 75(70,77) / CG 73(67,76)	At risk	At risk	Abe 2016: 12 weeks	Chen 2024: 12 weeks	Abe 2016: Regular exercise activities in nursing homes	Chen 2024: Maintain current physical activity	Abe 2016	Chen 2024
HMB+ARG+LYS vs Ca+HMB	HMB+ARG+LYS	Ca+HMB	IG: 71.50±1.5 / CG: 71.50±1.5	IG: 71.50±4.5 / CG: 71.60±4.2	At risk	Confirmed	Flakoll 2004: 12 weeks	Osuka 2021: 12 weeks	Flakoll 2004: No	Osuka 2021: No	Flakoll 2004	Osuka 2021
HMB+ARG+LYS vs P	HMB+ARG+LYS	P	IG: 71.50±1.5 / CG:	Björkman 2020: WP 83.60±4.7 /	At risk	Confirmed: 2; At risk: 3	Flakoll 2004: 12 weeks	Björkman 2020: 48 weeks;	Flakoll 2004: No	Björkman 2020: Low-intensity	Flakoll 2004	Björkman 2020; Ottestad

Indirect comparison	Node A	Node B	Age (A)	Age (B)	Sarcopenia status (A)	Sarcopenia status (B)	Intervention duration (A)	Intervention duration (B)	Background exercise (A)	Background exercise (B)	Supporting studies (A)	Supporting studies (B)
			71.50±1.5	P 84.0±3.9 / CG 83.70±5.1; Ottestad 2017: IG 76.80±6.2 / CG 77.10±4.7; Alemán-Mateo 2012: IG 75.40±5.0 / CG 76.70±5.8; Kao 2025: IG 72.60±5.1 / CG 72.30±5.2; Park 2018: WP 76.80±3.70 / P 77.30±3.67 / CG 76.83±3.86				Ottestad 2017: 12 weeks; Alemán-Mateo 2012: 12 weeks; Kao 2025: 12 weeks; Park 2018: 12 weeks		home workouts; Ottestad 2017: No; Alemán-Mateo 2012: No; Kao 2025: No; Park 2018: No		2017; Alemán-Mateo 2012; Kao 2025; Park 2018
HMB+ARG+LYS vs P+Ca	HMB+ARG+LYS	P+Ca	IG: 71.50±1.5 / CG: 71.50±1.5	Chen 2024: OG 71(67,73) / GG 75(70,77) / CG	At risk	At risk	Flakoll 2004: 12 weeks	Chen 2024: 12 weeks	Flakoll 2004: No	Chen 2024: Maintain current physical activity	Flakoll 2004	Chen 2024

Indirect comparison	Node A	Node B	Age (A)	Age (B)	Sarcopenia status (A)	Sarcopenia status (B)	Intervention duration (A)	Intervention duration (B)	Background exercise (A)	Background exercise (B)	Supporting studies (A)	Supporting studies (B)
Ca+HMB vs P	Ca+HMB	P	IG: 71.50±4.5 / CG: 71.60±4.2	73(67,76) Björkman 2020: WP 83.60±4.7 / P 84.0±3.9 / CG 83.70±5.1; Ottestad 2017: IG 76.80±6.2 / CG 77.10±4.7; Alemán-Mateo 2012: IG 75.40±5.0 / CG 76.70±5.8; Kao 2025: IG 72.60±5.1 / CG 72.30±5.2; Park 2018: WP 76.80±3.70 / P 77.30±3.67 / CG 76.83±3.86	Confirmed	Confirmed: 2; At risk: 3	Osuka 2021: 12 weeks	Björkman 2020: 48 weeks; Ottestad 2017: 12 weeks; Alemán-Mateo 2012: 12 weeks; Kao 2025: 12 weeks; Park 2018: 12 weeks	Osuka 2021: No	Björkman 2020: Low-intensity home workouts; Ottestad 2017: No; Alemán-Mateo 2012: No; Kao 2025: No; Park 2018: No	Osuka 2021	Björkman 2020; Ottestad 2017; Alemán-Mateo 2012; Kao 2025; Park 2018
Ca+HMB vs P+Ca	Ca+HMB	P+Ca	IG: 71.50±4.5 /	Chen 2024: OG	Confirmed	At risk	Osuka 2021: 12 weeks	Chen 2024: 12 weeks	Osuka 2021: No	Chen 2024: Maintain	Osuka 2021	Chen 2024

Indirect comparison	Node A	Node B	Age (A)	Age (B)	Sarcopenia status (A)	Sarcopenia status (B)	Intervention duration (A)	Intervention duration (B)	Background exercise (A)	Background exercise (B)	Supporting studies (A)	Supporting studies (B)
			CG: 71.60±4.2	71(67,73) / GG 75(70,77) / CG 73(67,76)						current physical activity		
P vs P+Ca	P	P+Ca	Björkman 2020: WP 83.60±4.7 / P 84.0±3.9 / CG 83.70±5.1; Ottestad 2017: IG 76.80±6.2 / CG 77.10±4.7; Alemán-Mateo 2012: IG 75.40±5.0 / CG 76.70±5.8; Kao 2025: IG 72.60±5.1 / CG 72.30±5.2; Park 2018: WP 76.80±3.70 / P 77.30±3.67 /	Chen 2024: OG 71(67,73) / GG 75(70,77) / CG 73(67,76)	Confirmed: 2; At risk: 3	At risk	Björkman 2020: 48 weeks; Ottestad 2017: 12 weeks; Alemán-Mateo 2012: 12 weeks; Kao 2025: 12 weeks; Park 2018: 12 weeks	Chen 2024: 12 weeks	Björkman 2020: Low-intensity home workouts; Ottestad 2017: No; Alemán-Mateo 2012: No; Kao 2025: No; Park 2018: No	Chen 2024: Maintain current physical activity	Björkman 2020; Ottestad 2017; Alemán-Mateo 2012; Kao 2025; Park 2018	Chen 2024

Indirect comparison	Node A	Node B	Age (A)	Age (B)	Sarcopenia status (A)	Sarcopenia status (B)	Intervention duration (A)	Intervention duration (B)	Background exercise (A)	Background exercise (B)	Supporting studies (A)	Supporting studies (B)
			CG 76.83±3.86									

Supplementary Table S5. Distribution of potential effect modifiers across indirect comparisons for gait speed

Indirect comparison	Node A	Node B	Age (A)	Age (B)	Sarcopenia status (A)	Sarcopenia status (B)	Intervention duration (A)	Intervention duration (B)	Background exercise (A)	Background exercise (B)	Supporting studies (A)	Supporting studies (B)
P+Ca+CaP vs WP	P+Ca +CaP	WP	OG: 71(67,73) / GG: 75(70,77) / CG: 73(67,76) (n=1 study record)	WP: 83.60±4.7 / P: 84.0±3.9 / CG: 83.70±5.1; WP: 76.80±3.70 / P: 77.30±3.67 / CG: 76.83±3.86 (n=2 study records)	At risk (1 study record)	Confirmed: 1; At risk: 1 study record	Chen 2024: 12 weeks	Björkman 2020: 48 weeks; Park, 2018: 12 weeks	Chen, 2024: All were instructed to maintain their current level of physical activity	Björkman, 2020: Low-Intensity Home Workouts; Park, 2018: No	Chen, 2024	Björkman, 2020; Park, 2018
P+Ca+CaP vs WP+VD+Leu	P+Ca +CaP	WP+V D+Leu	OG: 71(67,73) / GG: 75(70,77) / CG: 73(67,76) (n=1 study record)	IG: 72.50±5.57 / CG: 73.80±8.11; IG: 77.30±6.7 / CG: 78.10±7.0; IG: 81±7 / CG: 82±5; IG: 80.77±6.29 / CG: 80.21±8.54 (n=4 study records)	At risk (1 study record)	Confirmed (4 study records)	Chen 2024: 12 weeks	Lin 2021: 12 weeks; Bauer, 2015: 13 weeks; Rondanelli 2020: 6 weeks; Rondanelli 2016: 12 weeks	Chen, 2024: All were instructed to maintain their current level of physical activity	Lin, 2021: No; Bauer, 2015: No; Rondanelli, 2020: Personalized physical rehabilitation therapy 5 times a week, 20–30 minutes per session; Rondanelli, 2016: Structured	Chen, 2024	Lin, 2021; Bauer, 2015; Rondanelli, 2020; Rondanelli, 2016

Indirect comparison	Node A	Node B	Age (A)	Age (B)	Sarcopenia status (A)	Sarcopenia status (B)	Intervention duration (A)	Intervention duration (B)	Background exercise (A)	Background exercise (B)	Supporting studies (A)	Supporting studies (B)
										training 5 times a week, 20 minutes each session		
P+Ca+CaP vs WP+VD+VE	P+Ca +CaP	WP+V D+VE	OG: 71(67,73) / GG: 75(70,77) / CG: 73(67,76) (n=1 study record)	IG: 73.23±6.52 / CG: 74.83±5.94 (n=1 study record)	At risk (1 study record)	Confirmed (1 study record)	Chen 2024: 12 weeks	Bo, 2019: 24 weeks	Chen, 2024: All were instructed to maintain their current level of physical activity	Bo, 2019: No	Chen, 2024	Bo, 2019
P+Ca+CaP vs WP+Leu	P+Ca +CaP	WP+L eu	OG: 71(67,73) / GG: 75(70,77) / CG: 73(67,76) (n=1 study record)	LEU-PRO+n-3: 73±6 / LEU-PRO: 70±5 / CG: 73±7; IG: 68.80±6.5 / CG: 69.70±7.9 (n=2 study records)	At risk (1 study record)	At risk: 1; Confirmed: 1 study record	Chen 2024: 12 weeks	Murphy 2021: 24 weeks; Chung 2026: 12 weeks	Chen, 2024: All were instructed to maintain their current level of physical activity	Murphy, 2021: No; Chung, 2026: No	Chen, 2024	Murphy, 2021; Chung, 2026
P+Ca+CaP vs WP+Leu+LCT	P+Ca +CaP	WP+L eu+LC T	OG: 71(67,73) / GG: 75(70,77) / CG: 73(67,76) (n=1 study	LEU-PRO+n-3: 73±6 / LEU-PRO: 70±5 / CG: 73±7 (n=1 study record)	At risk (1 study record)	At risk (1 study record)	Chen 2024: 12 weeks	Murphy 2021: 24 weeks	Chen, 2024: All were instructed to maintain their current level of physical activity	Murphy, 2021: No	Chen, 2024	Murphy, 2021

Indirect comparison	Node A	Node B	Age (A)	Age (B)	Sarcopenia status (A)	Sarcopenia status (B)	Intervention duration (A)	Intervention duration (B)	Background exercise (A)	Background exercise (B)	Supporting studies (A)	Supporting studies (B)
			record)									
P+Ca+CaP vs Leu+VD+MCT	P+Ca +CaP	Leu+V D+MC T	OG: 71(67,73) / GG: 75(70,77) / CG: 73(67,76) (n=1 study record)	LD+MCT: 85.50±3.7 / LD+LCT: 87.70±3.9 / CG: 86.80±6.5 (n=1 study record)	At risk (1 study record)	At risk (1 study record)	Chen 2024: 12 weeks	Abe 2016: 12 weeks	Chen, 2024: All were instructed to maintain their current level of physical activity	Abe, 2016: Regular Exercise Activities in Nursing Homes	Chen, 2024	Abe, 2016
P+Ca+CaP vs Leu+VD+LCT	P+Ca +CaP	Leu+V D+LC T	OG: 71(67,73) / GG: 75(70,77) / CG: 73(67,76) (n=1 study record)	LD+MCT: 85.50±3.7 / LD+LCT: 87.70±3.9 / CG: 86.80±6.5 (n=1 study record)	At risk (1 study record)	At risk (1 study record)	Chen 2024: 12 weeks	Abe 2016: 12 weeks	Chen, 2024: All were instructed to maintain their current level of physical activity	Abe, 2016: Regular Exercise Activities in Nursing Homes	Chen, 2024	Abe, 2016
P+Ca+CaP vs Ca+HMB	P+Ca +CaP	Ca+H MB	OG: 71(67,73) / GG: 75(70,77) / CG: 73(67,76) (n=1 study record)	IG: 71.50±4.5 / CG: 71.60±4.2 (n=1 study record)	At risk (1 study record)	Confirmed (1 study record)	Chen 2024: 12 weeks	Osuka 2021: 12 weeks	Chen, 2024: All were instructed to maintain their current level of physical activity	Osuka, 2021: No	Chen, 2024	Osuka, 2021
P+Ca+CaP vs P	P+Ca +CaP	P	OG: 71(67,73) / GG: 75(70,77	WP: 83.60±4.7 / P: 84.0±3.9 / CG:	At risk (1 study record)	Confirmed: 2; At risk: 3 study records	Chen 2024: 12 weeks	Björkman 2020: 48 weeks; Ottestad,	Chen, 2024: All were instructed to maintain	Björkman, 2020: Low-Intensity Home	Chen, 2024	Björkman, 2020; Ottestad, 2017;

Indirect comparison	Node A	Node B	Age (A)	Age (B)	Sarcopenia status (A)	Sarcopenia status (B)	Intervention duration (A)	Intervention duration (B)	Background exercise (A)	Background exercise (B)	Supporting studies (A)	Supporting studies (B)
			) / CG: 73(67,76) (n=1 study record)	83.70±5.1; IG: 76.80±6.2 / CG: 77.10±4.7; IG: 75.40±5.0 / CG: 76.70±5.8; IG: 72.60±5.1 / CG: 72.30±5.2; WP: 76.80±3.70 / P: 77.30±3.67 / CG: 76.83±3.86 (n=5 study records)				2017: 12 weeks; Alemán-Mateo, 2012: 12 weeks; Kao 2025: 12 weeks; Park, 2018: 12 weeks	their current level of physical activity	Workouts; Ottestad, 2017: No; Alemán-Mateo, 2012: No; Kao, 2025: No; Park, 2018: No		Alemán-Mateo, 2012; Kao, 2025; Park, 2018
P+Ca+CaP vs WP+CaHMB	P+Ca +CaP	WP+C aHMB	OG: 71(67,73) / GG: 75(70,77) / CG: 73(67,76) (n=1 study record)	IG: 77(71,81) / CG: 77(71,81); IG: 77(71,81) / CG: 77(71,81) (n=2 study records)	At risk (1 study record)	Confirmed: 1; At risk: 1 study record	Chen 2024: 12 weeks	Cramer (sarcopenia subgroup) 2016: 24 weeks; Cramer (at-risk sarcopenia subgroup) 2016: 24 weeks	Chen, 2024: All were instructed to maintain their current level of physical activity	Cramer (sarcopenia subgroup), 2016: No; Cramer (at-risk sarcopenia subgroup), 2016: No	Chen, 2024	Cramer (sarcopenia subgroup), 2016; Cramer (at-risk sarcopenia subgroup), 2016

Indirect comparison	Node A	Node B	Age (A)	Age (B)	Sarcopenia status (A)	Sarcopenia status (B)	Intervention duration (A)	Intervention duration (B)	Background exercise (A)	Background exercise (B)	Supporting studies (A)	Supporting studies (B)
WP vs WP+VD+VE	WP	WP+VD+VE	WP: 83.60±4.7 / P: 84.0±3.9 / CG: 83.70±5.1; WP: 76.80±3.70 / P: 77.30±3.67 / CG: 76.83±3.86 (n=2 study records)	IG: 73.23±6.52 / CG: 74.83±5.94 (n=1 study record)	Confirmed: 1; At risk: 1 study record	Confirmed (1 study record)	Björkman 2020: 48 weeks; Park, 2018: 12 weeks	Bo, 2019: 24 weeks	Björkman, 2020: Low-Intensity Home Workouts; Park, 2018: No	Bo, 2019: No	Björkman, 2020; Park, 2018	Bo, 2019
WP vs WP+Leu	WP	WP+Leu	WP: 83.60±4.7 / P: 84.0±3.9 / CG: 83.70±5.1; WP: 76.80±3.70 / P: 77.30±3.67 / CG: 76.83±3.86 (n=2 study records)	LEU-PRO+n-3: 73±6 / LEU-PRO: 70±5 / CG: 73±7; IG: 68.80±6.5 / CG: 69.70±7.9 (n=2 study records)	Confirmed: 1; At risk: 1 study record	At risk: 1; Confirmed: 1 study record	Björkman 2020: 48 weeks; Park, 2018: 12 weeks	Murphy 2021: 24 weeks; Chung 2026: 12 weeks	Björkman, 2020: Low-Intensity Home Workouts; Park, 2018: No	Murphy, 2021: No; Chung, 2026: No	Björkman, 2020; Park, 2018	Murphy, 2021; Chung, 2026
WP vs WP+Leu+LCT	WP	WP+Leu+LC	WP: 83.60±4.	LEU-PRO+n-3:	Confirmed: 1; At risk: 1	At risk (1 study	Björkman 2020: 48	Murphy 2021: 24	Björkman, 2020: Low-	Murphy, 2021: No	Björkman, 2020; Park,	Murphy, 2021

Indirect comparison	Node A	Node B	Age (A)	Age (B)	Sarcopenia status (A)	Sarcopenia status (B)	Intervention duration (A)	Intervention duration (B)	Background exercise (A)	Background exercise (B)	Supporting studies (A)	Supporting studies (B)
		T	7 / P: 84.0±3.9 / CG: 83.70±5.1; WP: 76.80±3.70 / P: 77.30±3.67 / CG: 76.83±3.86 (n=2 study records)	73±6 / LEU-PRO: 70±5 / CG: 73±7 (n=1 study record)	study record	record)	weeks; Park, 2018: 12 weeks	weeks	Intensity Home Workouts; Park, 2018: No		2018	
WP vs Leu+VD+MCT	WP	Leu+VD+MCT	WP: 83.60±4.7 / P: 84.0±3.9 / CG: 83.70±5.1; WP: 76.80±3.70 / P: 77.30±3.67 / CG: 76.83±3.86 (n=2 study records)	LD+MCT: 85.50±3.7 / LD+LCT: 87.70±3.9 / CG: 86.80±6.5 (n=1 study record)	Confirmed: 1; At risk: 1 study record	At risk (1 study record)	Björkman 2020: 48 weeks; Park, 2018: 12 weeks	Abe 2016: 12 weeks	Björkman, 2020: Low-Intensity Home Workouts; Park, 2018: No	Abe, 2016: Regular Exercise Activities in Nursing Homes	Björkman, 2020; Park, 2018	Abe, 2016
WP vs Leu+VD+LCT	WP	Leu+VD+LCT	WP: 83.60±4.7 / P: 84.0±3.9	LD+MCT: 85.50±3.7 / LD+LCT: 87.70±3.9 /	Confirmed: 1; At risk: 1 study record	At risk (1 study record)	Björkman 2020: 48 weeks; Park, 2018: 12	Abe 2016: 12 weeks	Björkman, 2020: Low-Intensity Home	Abe, 2016: Regular Exercise Activities in	Björkman, 2020; Park, 2018	Abe, 2016

Indirect comparison	Node A	Node B	Age (A)	Age (B)	Sarcopenia status (A)	Sarcopenia status (B)	Intervention duration (A)	Intervention duration (B)	Background exercise (A)	Background exercise (B)	Supporting studies (A)	Supporting studies (B)
			/ CG: 83.70±5.1; WP: 76.80±3.70 / P: 77.30±3.67 / CG: 76.83±3.86 (n=2 study records)	CG: 86.80±6.5 (n=1 study record)			weeks		Workouts; Park, 2018: No	Nursing Homes		
WP vs Ca+HMB	WP	Ca+HMB	WP: 83.60±4.7 / P: 84.0±3.9 / CG: 83.70±5.1; WP: 76.80±3.70 / P: 77.30±3.67 / CG: 76.83±3.86 (n=2 study records)	IG: 71.50±4.5 / CG: 71.60±4.2 (n=1 study record)	Confirmed: 1; At risk: 1 study record	Confirmed (1 study record)	Björkman 2020: 48 weeks; Park, 2018: 12 weeks	Osuka 2021: 12 weeks	Björkman, 2020: Low-Intensity Home Workouts; Park, 2018: No	Osuka, 2021: No	Björkman, 2020; Park, 2018	Osuka, 2021
WP vs WP+CaHMB	WP	WP+CaHMB	WP: 83.60±4.7 / P: 84.0±3.9 / CG: 83.70±5.	IG: 77(71,81) / CG: 77(71,81); IG: 77(71,81) /	Confirmed: 1; At risk: 1 study record	Confirmed: 1; At risk: 1 study record	Björkman 2020: 48 weeks; Park, 2018: 12 weeks	Cramer (sarcopenia subgroup) 2016: 24 weeks; Cramer (at-	Björkman, 2020: Low-Intensity Home Workouts; Park, 2018:	Cramer (sarcopenia subgroup), 2016: No; Cramer (at-risk	Björkman, 2020; Park, 2018	Cramer (sarcopenia subgroup), 2016; Cramer (at-risk

Indirect comparison	Node A	Node B	Age (A)	Age (B)	Sarcopenia status (A)	Sarcopenia status (B)	Intervention duration (A)	Intervention duration (B)	Background exercise (A)	Background exercise (B)	Supporting studies (A)	Supporting studies (B)
			1; WP: 76.80±3.70 / P: 77.30±3.67 / CG: 76.83±3.86 (n=2 study records)	CG: 77(71,81) (n=2 study records)				risk sarcopenia subgroup) 2016: 24 weeks	No	sarcopenia subgroup), 2016: No		sarcopenia subgroup), 2016
WP+VD+Leu vs WP+VD+VE	WP+VD+Leu	WP+VD+VE	IG: 72.50±5.57 / CG: 73.80±8.11; IG: 77.30±6.7 / CG: 78.10±7.0; IG: 81±7 / CG: 82±5; IG: 80.77±6.29 / CG: 80.21±8.54 (n=4 study records)	IG: 73.23±6.52 / CG: 74.83±5.94 (n=1 study record)	Confirmed (4 study records)	Confirmed (1 study record)	Lin 2021: 12 weeks; Bauer, 2015: 13 weeks; Rondanelli 2020: 6 weeks; Rondanelli 2016: 12 weeks	Bo, 2019: 24 weeks	Lin, 2021: No; Bauer, 2015: No; Rondanelli, 2020: Personalized physical rehabilitation therapy 5 times a week, 20–30 minutes per session; Rondanelli, 2016: Structured training 5 times a week, 20 minutes per session	Bo, 2019: No	Lin, 2021; Bauer, 2015; Rondanelli, 2020; Rondanelli, 2016	Bo, 2019
WP+VD+Leu vs WP+Leu	WP+VD+Leu	WP+Leu	IG: 72.50±5.57 / CG:	LEU-PRO+n-3: 73±6 / LEU-	Confirmed (4 study records)	At risk: 1; Confirmed: 1 study	Lin 2021: 12 weeks; Bauer, 2015:	Murphy 2021: 24 weeks;	Lin, 2021: No; Bauer, 2015: No;	Murphy, 2021: No; Chung, 2026:	Lin, 2021; Bauer, 2015;	Murphy, 2021; Chung,

Indirect comparison	Node A	Node B	Age (A)	Age (B)	Sarcopenia status (A)	Sarcopenia status (B)	Intervention duration (A)	Intervention duration (B)	Background exercise (A)	Background exercise (B)	Supporting studies (A)	Supporting studies (B)
			73.80±8.11; IG: 77.30±6.7 / CG: 78.10±7.0; IG: 81±7 / CG: 82±5; IG: 80.77±6.29 / CG: 80.21±8.54 (n=4 study records)	PRO: 70±5 / CG: 73±7; IG: 68.80±6.5 / CG: 69.70±7.9 (n=2 study records)		record	13 weeks; Rondanelli 2020: 6 weeks; Rondanelli 2016: 12 weeks	Chung 2026: 12 weeks	Rondanelli, 2020: Personalized physical rehabilitation therapy 5 times a week, 20–30 minutes per session; Rondanelli, 2016: Structured training 5 times a week, 20 minutes each session	No	Rondanelli, 2020; Rondanelli, 2016	2026
WP+VD+Leu vs WP+Leu+LCT	WP+VD+Leu	WP+Leu+LCT	IG: 72.50±5.57 / CG: 73.80±8.11; IG: 77.30±6.7 / CG: 78.10±7.0; IG: 81±7 / CG: 82±5; IG: 80.77±6.29 / CG: 80.21±8.	LEU-PRO+n-3: 73±6 / LEU-PRO: 70±5 / CG: 73±7 (n=1 study record)	Confirmed (4 study records)	At risk (1 study record)	Lin 2021: 12 weeks; Bauer, 2015: 13 weeks; Rondanelli 2020: 6 weeks; Rondanelli 2016: 12 weeks	Murphy 2021: 24 weeks	Lin, 2021: No; Bauer, 2015: No; Rondanelli, 2020: Personalized physical rehabilitation therapy 5 times a week, 20–30 minutes per session; Rondanelli, 2016:	Murphy, 2021: No	Lin, 2021; Bauer, 2015; Rondanelli, 2020; Rondanelli, 2016	Murphy, 2021

Indirect comparison	Node A	Node B	Age (A)	Age (B)	Sarcopenia status (A)	Sarcopenia status (B)	Intervention duration (A)	Intervention duration (B)	Background exercise (A)	Background exercise (B)	Supporting studies (A)	Supporting studies (B)
			54 (n=4 study records)						Structured training 5 times a week, 20 minutes per session			
WP+VD+Leu vs Leu+VD+MCT	WP+VD+Leu	Leu+VD+MCT	IG: 72.50±5.57 / CG: 73.80±8.11; IG: 77.30±6.7 / CG: 78.10±7.0; IG: 81±7 / CG: 82±5; IG: 80.77±6.29 / CG: 80.21±8.54 (n=4 study records)	LD+MCT: 85.50±3.7 / LD+LCT: 87.70±3.9 / CG: 86.80±6.5 (n=1 study record)	Confirmed (4 study records)	At risk (1 study record)	Lin 2021: 12 weeks; Bauer, 2015: 13 weeks; Rondanelli 2020: 6 weeks; Rondanelli 2016: 12 weeks	Abe 2016: 12 weeks	Lin, 2021: No; Bauer, 2015: No; Rondanelli, 2020: Personalized physical rehabilitation therapy 5 times a week, 20–30 minutes per session; Rondanelli, 2016: Structured training 5 times a week, 20 minutes each session	Abe, 2016: Regular Exercise Activities in Nursing Homes	Lin, 2021; Bauer, 2015; Rondanelli, 2020; Rondanelli, 2016	Abe, 2016
WP+VD+Leu vs Leu+VD+LCT	WP+VD+Leu	Leu+VD+LCT	IG: 72.50±5.57 / CG: 73.80±8.11; IG: 77.30±6.7 / CG:	LD+MCT: 85.50±3.7 / LD+LCT: 87.70±3.9 / CG: 86.80±6.5 (n=1 study	Confirmed (4 study records)	At risk (1 study record)	Lin 2021: 12 weeks; Bauer, 2015: 13 weeks; Rondanelli 2020: 6 weeks;	Abe 2016: 12 weeks	Lin, 2021: No; Bauer, 2015: No; Rondanelli, 2020: Personalized physical	Abe, 2016: Regular Exercise Activities in Nursing Homes	Lin, 2021; Bauer, 2015; Rondanelli, 2020; Rondanelli, 2016	Abe, 2016

Indirect comparison	Node A	Node B	Age (A)	Age (B)	Sarcopenia status (A)	Sarcopenia status (B)	Intervention duration (A)	Intervention duration (B)	Background exercise (A)	Background exercise (B)	Supporting studies (A)	Supporting studies (B)
			78.10±7.0; IG: 81±7 / CG: 82±5; IG: 80.77±6.29 / CG: 80.21±8.54 (n=4 study records)	record)			Rondanelli 2016: 12 weeks		rehabilitation therapy 5 times a week, 20–30 minutes per session; Rondanelli, 2016: Structured training 5 times a week, 20 minutes each session			
WP+VD+Leu vs Ca+HMB	WP+VD+Leu	Ca+HMB	IG: 72.50±5.57 / CG: 73.80±8.11; IG: 77.30±6.7 / CG: 78.10±7.0; IG: 81±7 / CG: 82±5; IG: 80.77±6.29 / CG: 80.21±8.54 (n=4 study records)	IG: 71.50±4.5 / CG: 71.60±4.2 (n=1 study record)	Confirmed (4 study records)	Confirmed (1 study record)	Lin 2021: 12 weeks; Bauer, 2015: 13 weeks; Rondanelli 2020: 6 weeks; Rondanelli 2016: 12 weeks	Osuka 2021: 12 weeks	Lin, 2021: No; Bauer, 2015: No; Rondanelli, 2020: Personalized physical rehabilitation therapy 5 times a week, 20–30 minutes per session; Rondanelli, 2016: Structured training 5 times a week, 20 minutes	Osuka, 2021: No	Lin, 2021; Bauer, 2015; Rondanelli, 2020; Rondanelli, 2016	Osuka, 2021

Indirect comparison	Node A	Node B	Age (A)	Age (B)	Sarcopenia status (A)	Sarcopenia status (B)	Intervention duration (A)	Intervention duration (B)	Background exercise (A)	Background exercise (B)	Supporting studies (A)	Supporting studies (B)
									each session			
WP+VD+Leu vs P	WP+ VD+ Leu	P	IG: 72.50±5. 57 / CG: 73.80±8. 11; IG: 77.30±6. 7 / CG: 78.10±7. 0; IG: 81±7 / CG: 82±5; IG: 80.77±6. 29 / CG: 80.21±8. 54 (n=4 study records)	WP: 83.60±4.7 / P: 84.0±3.9 / CG: 83.70±5.1; IG: 76.80±6.2 / CG: 77.10±4.7; IG: 75.40±5.0 / CG: 76.70±5.8; IG: 72.60±5.1 / CG: 72.30±5.2; WP: 76.80±3.70 / P: 77.30±3.67 / CG: 76.83±3.86 (n=5 study records)	Confirmed (4 study records)	Confirmed: 2; At risk: 3 study records	Lin 2021: 12 weeks; Bauer, 2015: 13 weeks; Rondanelli 2020: 6 weeks; Rondanelli 2016: 12 weeks	Björkman 2020: 48 weeks; Ottestad, 2017: 12 weeks; Alemán-Mateo, 2012: 12 weeks; Kao 2025: 12 weeks; Park, 2018: 12 weeks	Lin, 2021: No; Bauer, 2015: No; Rondanelli, 2020: Personalized physical rehabilitation therapy 5 times a week, 20–30 minutes per session; Rondanelli, 2016: Structured training 5 times a week, 20 minutes per session	Björkman, 2020: Low-Intensity Home Workouts; Ottestad, 2017: No; Alemán-Mateo, 2012: No; Kao, 2025: No; Park, 2018: No	Lin, 2021; Bauer, 2015; Rondanelli, 2020; Rondanelli, 2016	Björkman, 2020; Ottestad, 2017; Alemán-Mateo, 2012; Kao, 2025; Park, 2018
WP+VD+Leu vs P+Ca	WP+ VD+ Leu	P+Ca	IG: 72.50±5. 57 / CG: 73.80±8. 11; IG: 77.30±6.	OG: 71(67,73) / GG: 75(70,77) / CG: 73(67,76)	Confirmed (4 study records)	At risk (1 study record)	Lin 2021: 12 weeks; Bauer, 2015: 13 weeks; Rondanelli 2020: 6	Chen 2024: 12 weeks	Lin, 2021: No; Bauer, 2015: No; Rondanelli, 2020: Personalized	Chen, 2024: All were instructed to maintain their current level of	Lin, 2021; Bauer, 2015; Rondanelli, 2020; Rondanelli,	Chen, 2024

Indirect comparison	Node A	Node B	Age (A)	Age (B)	Sarcopenia status (A)	Sarcopenia status (B)	Intervention duration (A)	Intervention duration (B)	Background exercise (A)	Background exercise (B)	Supporting studies (A)	Supporting studies (B)
			7 / CG: 78.10±7.0; IG: 81±7 / CG: 82±5; IG: 80.77±6.29 / CG: 80.21±8.54 (n=4 study records)	(n=1 study record)			weeks; Rondanelli 2016: 12 weeks		physical rehabilitation therapy 5 times a week, 20–30 minutes per session; Rondanelli, 2016: Structured training 5 times a week, 20 minutes each session	physical activity	2016	
WP+VD+Leu vs WP+CaHMB	WP+VD+Leu	WP+CaHMB	IG: 72.50±5.57 / CG: 73.80±8.11; IG: 77.30±6.7 / CG: 78.10±7.0; IG: 81±7 / CG: 82±5; IG: 80.77±6.29 / CG: 80.21±8.54 (n=4 study records)	IG: 77(71,81) / CG: 77(71,81); IG: 77(71,81) / CG: 77(71,81) (n=2 study records)	Confirmed (4 study records)	Confirmed: 1; At risk: 1 study record	Lin 2021: 12 weeks; Bauer, 2015: 13 weeks; Rondanelli 2020: 6 weeks; Rondanelli 2016: 12 weeks	Cramer (sarcopenia subgroup) 2016: 24 weeks; Cramer (at-risk sarcopenia subgroup) 2016: 24 weeks	Lin, 2021: No; Bauer, 2015: No; Rondanelli, 2020: Personalized physical rehabilitation therapy 5 times a week, 20–30 minutes per session; Rondanelli, 2016: Structured training 5 times a week,	Cramer (sarcopenia subgroup), 2016: No; Cramer (at-risk sarcopenia subgroup), 2016: No	Lin, 2021; Bauer, 2015; Rondanelli, 2020; Rondanelli, 2016	Cramer (sarcopenia subgroup), 2016; Cramer (at-risk sarcopenia subgroup), 2016

Indirect comparison	Node A	Node B	Age (A)	Age (B)	Sarcopenia status (A)	Sarcopenia status (B)	Intervention duration (A)	Intervention duration (B)	Background exercise (A)	Background exercise (B)	Supporting studies (A)	Supporting studies (B)
									20 minutes each session			
WP+VD+VE vs WP+Leu	WP+VD+VE	WP+Leu	IG: 73.23±6.52 / CG: 74.83±5.94 (n=1 study record)	LEU-PRO+n-3: 73±6 / LEU-PRO: 70±5 / CG: 73±7; IG: 68.80±6.5 / CG: 69.70±7.9 (n=2 study records)	Confirmed (1 study record)	At risk: 1; Confirmed: 1 study record	Bo, 2019: 24 weeks	Murphy 2021: 24 weeks; Chung 2026: 12 weeks	Bo, 2019: No	Murphy, 2021: No; Chung, 2026: No	Bo, 2019	Murphy, 2021; Chung, 2026
WP+VD+VE vs WP+Leu+LCT	WP+VD+VE	WP+Leu+LCT	IG: 73.23±6.52 / CG: 74.83±5.94 (n=1 study record)	LEU-PRO+n-3: 73±6 / LEU-PRO: 70±5 / CG: 73±7 (n=1 study record)	Confirmed (1 study record)	At risk (1 study record)	Bo, 2019: 24 weeks	Murphy 2021: 24 weeks	Bo, 2019: No	Murphy, 2021: No	Bo, 2019	Murphy, 2021
WP+VD+VE vs Leu+VD+MCT	WP+VD+VE	Leu+VD+MCT	IG: 73.23±6.52 / CG: 74.83±5.94 (n=1 study record)	LD+MCT: 85.50±3.7 / LD+LCT: 87.70±3.9 / CG: 86.80±6.5 (n=1 study record)	Confirmed (1 study record)	At risk (1 study record)	Bo, 2019: 24 weeks	Abe, 2016: 12 weeks	Bo, 2019: No	Abe, 2016: Regular Exercise Activities in Nursing Homes	Bo, 2019	Abe, 2016
WP+VD+VE vs Leu+VD+LCT	WP+VD+VE	Leu+VD+LCT	IG: 73.23±6.52 / CG: 74.83±5.	LD+MCT: 85.50±3.7 / LD+LCT: 87.70±3.9 /	Confirmed (1 study record)	At risk (1 study record)	Bo, 2019: 24 weeks	Abe, 2016: 12 weeks	Bo, 2019: No	Abe, 2016: Regular Exercise Activities in	Bo, 2019	Abe, 2016

Indirect comparison	Node A	Node B	Age (A)	Age (B)	Sarcopenia status (A)	Sarcopenia status (B)	Intervention duration (A)	Intervention duration (B)	Background exercise (A)	Background exercise (B)	Supporting studies (A)	Supporting studies (B)
			94 (n=1 study record)	CG: 86.80±6.5 (n=1 study record)						Nursing Homes		
WP+VD+VE vs Ca+HMB	WP+ VD+ VE	Ca+H MB	IG: 73.23±6. 52 / CG: 74.83±5. 94 (n=1 study record)	IG: 71.50±4.5 / CG: 71.60±4.2 (n=1 study record)	Confirmed (1 study record)	Confirmed (1 study record)	Bo, 2019: 24 weeks	Osuka, 2021: 12 weeks	Bo, 2019: No	Osuka, 2021: No	Bo, 2019	Osuka, 2021
WP+VD+VE vs P	WP+ VD+ VE	P	IG: 73.23±6. 52 / CG: 74.83±5. 94 (n=1 study record)	WP: 83.60±4.7 / P: 84.0±3.9 / CG: 83.70±5.1; IG: 76.80±6.2 / CG: 77.10±4.7; IG: 75.40±5.0 / CG: 76.70±5.8; IG: 72.60±5.1 / CG: 72.30±5.2; WP: 76.80±3.70 / P: 77.30±3.67 /	Confirmed (1 study record)	Confirmed: 2; At risk: 3 study records	Bo, 2019: 24 weeks	Björkman 2020: 48 weeks; Ottestad, 2017: 12 weeks; Alemán-Mateo, 2012: 12 weeks; Kao 2025: 12 weeks; Park, 2018: 12 weeks	Bo, 2019: No	Björkman, 2020: Low-Intensity Home Workouts; Ottestad, 2017: No; Alemán-Mateo, 2012: No; Kao, 2025: No; Park, 2018: No	Bo, 2019	Björkman, 2020; Ottestad, 2017; Alemán-Mateo, 2012; Kao, 2025; Park, 2018

Indirect comparison	Node A	Node B	Age (A)	Age (B)	Sarcopenia status (A)	Sarcopenia status (B)	Intervention duration (A)	Intervention duration (B)	Background exercise (A)	Background exercise (B)	Supporting studies (A)	Supporting studies (B)
WP+VD+VE vs P+Ca	WP+ VD+ VE	P+Ca	IG: 73.23±6. 52 / CG: 74.83±5. 94 (n=1 study record)	CG: 76.83±3.86 (n=5 study records) OG: 71(67,73) / GG: 75(70,77) / CG: 73(67,76) (n=1 study record)	Confirmed (1 study record)	At risk (1 study record)	Bo, 2019: 24 weeks	Chen, 2024: 12 weeks	Bo, 2019: No	Chen, 2024: All were instructed to maintain their current level of physical activity	Bo, 2019	Chen, 2024
WP+VD+VE vs WP+CaHMB	WP+ VD+ VE	WP+C aHMB	IG: 73.23±6. 52 / CG: 74.83±5. 94 (n=1 study record)	IG: 77(71,81) / CG: 77(71,81); IG: 77(71,81) / CG: 77(71,81) (n=2 study records)	Confirmed (1 study record)	Confirmed: 1; At risk: 1 study record	Bo, 2019: 24 weeks	Cramer (sarcopenia subgroup) 2016: 24 weeks; Cramer (at-risk sarcopenia subgroup) 2016: 24 weeks	Bo, 2019: No	Cramer (sarcopenia subgroup), 2016: No; Cramer (at-risk sarcopenia subgroup), 2016: No	Bo, 2019	Cramer (sarcopenia subgroup), 2016; Cramer (at-risk sarcopenia subgroup), 2016
WP+Leu vs Leu+VD+MCT	WP+ Leu	Leu+V D+MC T	LEU-PRO+n-3: 73±6 / LEU-PRO: 70±5 / CG: 73±7; IG: 68.80±6.	LD+MCT: 85.50±3.7 / LD+LCT: 87.70±3.9 / CG: 86.80±6.5 (n=1 study record)	At risk: 1; Confirmed: 1 study record	At risk (1 study record)	Murphy 2021: 24 weeks; Chung 2026: 12 weeks	Abe 2016: 12 weeks	Murphy, 2021: No; Chung, 2026: No	Abe, 2016: Regular Exercise Activities in Nursing Homes	Murphy, 2021; Chung, 2026	Abe, 2016

Indirect comparison	Node A	Node B	Age (A)	Age (B)	Sarcopenia status (A)	Sarcopenia status (B)	Intervention duration (A)	Intervention duration (B)	Background exercise (A)	Background exercise (B)	Supporting studies (A)	Supporting studies (B)
			5 / CG: 69.70±7.9 (n=2 study records)									
WP+Leu vs Leu+VD+LCT	WP+Leu	Leu+VD+LCT	LEU-PRO+n-3: 73±6 / LEU-PRO: 70±5 / CG: 73±7; IG: 68.80±6.5 / CG: 69.70±7.9 (n=2 study records)	LD+MCT: 85.50±3.7 / LD+LCT: 87.70±3.9 / CG: 86.80±6.5 (n=1 study record)	At risk: 1; Confirmed: 1 study record	At risk (1 study record)	Murphy 2021: 24 weeks; Chung 2026: 12 weeks	Abe 2016: 12 weeks	Murphy, 2021: No; Chung, 2026: No	Abe, 2016: Regular Exercise Activities in Nursing Homes	Murphy, 2021; Chung, 2026	Abe, 2016
WP+Leu vs Ca+HMB	WP+Leu	Ca+HMB	LEU-PRO+n-3: 73±6 / LEU-PRO: 70±5 / CG: 73±7; IG: 68.80±6.5 / CG: 69.70±7.9 (n=2 study	IG: 71.50±4.5 / CG: 71.60±4.2 (n=1 study record)	At risk: 1; Confirmed: 1 study record	Confirmed (1 study record)	Murphy 2021: 24 weeks; Chung 2026: 12 weeks	Osuka 2021: 12 weeks	Murphy, 2021: No; Chung, 2026: No	Osuka, 2021: No	Murphy, 2021; Chung, 2026	Osuka, 2021

Indirect comparison	Node A	Node B	Age (A)	Age (B)	Sarcopenia status (A)	Sarcopenia status (B)	Intervention duration (A)	Intervention duration (B)	Background exercise (A)	Background exercise (B)	Supporting studies (A)	Supporting studies (B)
			records)									
WP+Leu vs P	WP+ Leu	P	LEU-PRO+n-3: 73±6 / LEU-PRO: 70±5 / CG: 73±7; IG: 68.80±6.5 / CG: 69.70±7.9 (n=2 study records)	WP: 83.60±4.7 / P: 84.0±3.9 / CG: 83.70±5.1; IG: 76.80±6.2 / CG: 77.10±4.7; IG: 75.40±5.0 / CG: 76.70±5.8; IG: 72.60±5.1 / CG: 72.30±5.2; WP: 76.80±3.70 / P: 77.30±3.67 / CG: 76.83±3.86 (n=5 study records)	At risk: 1; Confirmed: 1 study record	Confirmed: 2; At risk: 3 study records	Murphy 2021: 24 weeks; Chung 2026: 12 weeks	Björkman 2020: 48 weeks; Ottestad, 2017: 12 weeks; Alemán-Mateo, 2012: 12 weeks; Kao 2025: 12 weeks; Park, 2018: 12 weeks	Murphy, 2021: No; Chung, 2026: No	Björkman, 2020: Low-Intensity Home Workouts; Ottestad, 2017: No; Alemán-Mateo, 2012: No; Kao, 2025: No; Park, 2018: No	Murphy, 2021; Chung, 2026	Björkman, 2020; Ottestad, 2017; Alemán-Mateo, 2012; Kao, 2025; Park, 2018
WP+Leu vs P+Ca	WP+ Leu	P+Ca	LEU-PRO+n-3: 73±6 / LEU-PRO: 70±5 /	OG: 71(67,73) / GG: 75(70,77) / CG: 73(67,76)	At risk: 1; Confirmed: 1 study record	At risk (1 study record)	Murphy 2021: 24 weeks; Chung 2026: 12 weeks	Chen 2024: 12 weeks	Murphy, 2021: No; Chung, 2026: No	Chen, 2024: All were instructed to maintain their current level of	Murphy, 2021; Chung, 2026	Chen, 2024

Indirect comparison	Node A	Node B	Age (A)	Age (B)	Sarcopenia status (A)	Sarcopenia status (B)	Intervention duration (A)	Intervention duration (B)	Background exercise (A)	Background exercise (B)	Supporting studies (A)	Supporting studies (B)
			CG: 73±7; IG: 68.80±6.5 / CG: 69.70±7.9 (n=2 study records)	(n=1 study record)						physical activity		
WP+Leu vs WP+CaHMB	WP+Leu	WP+CaHMB	LEU-PRO+n-3: 73±6 / LEU-PRO: 70±5 / CG: 73±7; IG: 68.80±6.5 / CG: 69.70±7.9 (n=2 study records)	IG: 77(71,81) / CG: 77(71,81); IG: 77(71,81) / CG: 77(71,81) (n=2 study records)	At risk: 1; Confirmed: 1 study record	Confirmed: 1; At risk: 1 study record	Murphy 2021: 24 weeks; Chung 2026: 12 weeks	Cramer (sarcopenia subgroup) 2016: 24 weeks; Cramer (at-risk sarcopenia subgroup) 2016: 24 weeks	Murphy, 2021: No; Chung, 2026: No	Cramer (sarcopenia subgroup), 2016: No; Cramer (at-risk sarcopenia subgroup), 2016: No	Murphy, 2021; Chung, 2026	Cramer (sarcopenia subgroup), 2016; Cramer (at-risk sarcopenia subgroup), 2016
WP+Leu+LCT vs Leu+VD+MCT	WP+Leu+LCT	Leu+VD+MCT	LEU-PRO+n-3: 73±6 / LEU-PRO: 70±5 / CG: 73±7 (n=1 study	LD+MCT: 85.50±3.7 / LD+LCT: 87.70±3.9 / CG: 86.80±6.5 (n=1 study record)	At risk (1 study record)	At risk (1 study record)	Murphy 2021: 24 weeks	Abe 2016: 12 weeks	Murphy, 2021: No	Abe, 2016: Regular Exercise Activities in Nursing Homes	Murphy, 2021	Abe, 2016

Indirect comparison	Node A	Node B	Age (A)	Age (B)	Sarcopenia status (A)	Sarcopenia status (B)	Intervention duration (A)	Intervention duration (B)	Background exercise (A)	Background exercise (B)	Supporting studies (A)	Supporting studies (B)
			record)									
WP+Leu+LCT vs Leu+VD+LCT	WP+ Leu+ LCT	Leu+V D+LC T	LEU-PRO+n-3: 73±6 / LEU-PRO: 70±5 / CG: 73±7 (n=1 study record)	LD+MCT: 85.50±3.7 / LD+LCT: 87.70±3.9 / CG: 86.80±6.5 (n=1 study record)	At risk (1 study record)	At risk (1 study record)	Murphy 2021: 24 weeks	Abe 2016: 12 weeks	Murphy, 2021: No	Abe, 2016: Regular Exercise Activities in Nursing Homes	Murphy, 2021	Abe, 2016
WP+Leu+LCT vs Ca+HMB	WP+ Leu+ LCT	Ca+H MB	LEU-PRO+n-3: 73±6 / LEU-PRO: 70±5 / CG: 73±7 (n=1 study record)	IG: 71.50±4.5 / CG: 71.60±4.2 (n=1 study record)	At risk (1 study record)	Confirmed (1 study record)	Murphy 2021: 24 weeks	Osuka 2021: 12 weeks	Murphy, 2021: No	Osuka, 2021: No	Murphy, 2021	Osuka, 2021
WP+Leu+LCT vs P	WP+ Leu+ LCT	P	LEU-PRO+n-3: 73±6 / LEU-PRO: 70±5 / CG: 73±7 (n=1	WP: 83.60±4.7 / P: 84.0±3.9 / CG: 83.70±5.1; IG: 76.80±6.2 / CG: 77.10±4.7;	At risk (1 study record)	Confirmed: 2; At risk: 3 study records	Murphy 2021: 24 weeks	Björkman 2020: 48 weeks; Ottestad, 2017: 12 weeks; Alemán-Mateo, 2012: 12 weeks;	Murphy, 2021: No	Björkman, 2020: Low-Intensity Home Workouts; Ottestad, 2017: No; Alemán-Mateo, 2012:	Murphy, 2021	Björkman, 2020; Ottestad, 2017; Alemán-Mateo, 2012; Kao, 2025; Park, 2018

Indirect comparison	Node A	Node B	Age (A)	Age (B)	Sarcopenia status (A)	Sarcopenia status (B)	Intervention duration (A)	Intervention duration (B)	Background exercise (A)	Background exercise (B)	Supporting studies (A)	Supporting studies (B)
			study record)	IG: 75.40±5.0 / CG: 76.70±5.8; IG: 72.60±5.1 / CG: 72.30±5.2; WP: 76.80±3.70 / P: 77.30±3.67 / CG: 76.83±3.86 (n=5 study records)				Kao 2025: 12 weeks; Park, 2018: 12 weeks		No; Kao, 2025: No; Park, 2018: No		
WP+Leu+LCT vs P+Ca	WP+ Leu+ LCT	P+Ca	LEU-PRO+n-3: 73±6 / LEU-PRO: 70±5 / CG: 73±7 (n=1 study record)	OG: 71(67,73) / GG: 75(70,77) / CG: 73(67,76) (n=1 study record)	At risk (1 study record)	At risk (1 study record)	Murphy 2021: 24 weeks	Chen 2024: 12 weeks	Murphy, 2021: No	Chen, 2024: All were instructed to maintain their current level of physical activity	Murphy, 2021	Chen, 2024
WP+Leu+LCT vs WP+CaHMB	WP+ Leu+ LCT	WP+C aHMB	LEU-PRO+n-3: 73±6 / LEU-PRO:	IG: 77(71,81) / CG: 77(71,81); IG:	At risk (1 study record)	Confirmed: 1; At risk: 1 study record	Murphy 2021: 24 weeks	Cramer (sarcopenia subgroup) 2016: 24 weeks;	Murphy, 2021: No	Cramer (sarcopenia subgroup), 2016: No; Cramer (at-	Murphy, 2021	Cramer (sarcopenia subgroup), 2016; Cramer (at-

Indirect comparison	Node A	Node B	Age (A)	Age (B)	Sarcopenia status (A)	Sarcopenia status (B)	Intervention duration (A)	Intervention duration (B)	Background exercise (A)	Background exercise (B)	Supporting studies (A)	Supporting studies (B)
			70±5 / CG: 73±7 (n=1 study record)	77(71,81) / CG: 77(71,81) (n=2 study records)				Cramer (at-risk sarcopenia subgroup) 2016: 24 weeks		risk sarcopenia subgroup), 2016: No		risk sarcopenia subgroup), 2016
Leu+VD+MCT vs Ca+HMB	Leu+ VD+ MCT	Ca+H MB	LD+MCT: 85.50±3.7 / LD+LCT: 87.70±3.9 / CG: 86.80±6.5 (n=1 study record)	IG: 71.50±4.5 / CG: 71.60±4.2 (n=1 study record)	At risk (1 study record)	Confirmed (1 study record)	Abe 2016: 12 weeks	Osuka 2021: 12 weeks	Abe, 2016: Regular Exercise Activities in Nursing Homes	Osuka, 2021: No	Abe, 2016	Osuka, 2021
Leu+VD+MCT vs P	Leu+ VD+ MCT	P	LD+MCT: 85.50±3.7 / LD+LCT: 87.70±3.9 / CG: 86.80±6.5 (n=1 study record)	WP: 83.60±4.7 / P: 84.0±3.9 / CG: 83.70±5.1; IG: 76.80±6.2 / CG: 77.10±4.7; IG: 75.40±5.0 / CG: 76.70±5.8; IG:	At risk (1 study record)	Confirmed: 2; At risk: 3 study records	Abe 2016: 12 weeks	Björkman 2020: 48 weeks; Ottestad, 2017: 12 weeks; Alemán-Mateo, 2012: 12 weeks; Kao 2025: 12 weeks; Park, 2018: 12 weeks	Abe, 2016: Regular Exercise Activities in Nursing Homes	Björkman, 2020: Low-Intensity Home Workouts; Ottestad, 2017: No; Alemán-Mateo, 2012: No; Kao, 2025: No; Park, 2018: No	Abe, 2016	Björkman, 2020; Ottestad, 2017; Alemán-Mateo, 2012; Kao, 2025; Park, 2018

Indirect comparison	Node A	Node B	Age (A)	Age (B)	Sarcopenia status (A)	Sarcopenia status (B)	Intervention duration (A)	Intervention duration (B)	Background exercise (A)	Background exercise (B)	Supporting studies (A)	Supporting studies (B)
				72.60±5.1 / CG: 72.30±5.2; WP: 76.80±3.70 / P: 77.30±3.67 / CG: 76.83±3.86 (n=5 study records)								
Leu+VD+MCT vs P+Ca	Leu+ VD+ MCT	P+Ca	LD+MCT: 85.50±3.7 / LD+LCT: 87.70±3.9 / CG: 86.80±6.5 (n=1 study record)	OG: 71(67,73) / GG: 75(70,77) / CG: 73(67,76) (n=1 study record)	At risk (1 study record)	At risk (1 study record)	Abe 2016: 12 weeks	Chen 2024: 12 weeks	Abe, 2016: Regular Exercise Activities in Nursing Homes	Chen, 2024: All were instructed to maintain their current level of physical activity	Abe, 2016	Chen, 2024
Leu+VD+MCT vs WP+CaHMB	Leu+ VD+ MCT	WP+CaHMB	LD+MCT: 85.50±3.7 / LD+LCT: 87.70±3.9 / CG: 86.80±6.	IG: 77(71,81) / CG: 77(71,81); IG: 77(71,81) / CG: 77(71,81) (n=2 study	At risk (1 study record)	Confirmed: 1; At risk: 1 study record	Abe 2016: 12 weeks	Cramer (sarcopenia subgroup) 2016: 24 weeks; Cramer (at-risk sarcopenia subgroup)	Abe, 2016: Regular Exercise Activities in Nursing Homes	Cramer (sarcopenia subgroup), 2016: No; Cramer (at-risk sarcopenia subgroup), 2016: No	Abe, 2016	Cramer (sarcopenia subgroup), 2016; Cramer (at-risk sarcopenia subgroup), 2016

Indirect comparison	Node A	Node B	Age (A)	Age (B)	Sarcopenia status (A)	Sarcopenia status (B)	Intervention duration (A)	Intervention duration (B)	Background exercise (A)	Background exercise (B)	Supporting studies (A)	Supporting studies (B)
			5 (n=1 study record)	records)				2016: 24 weeks				
Leu+VD+LCT vs Ca+HMB	Leu+ VD+ LCT	Ca+H MB	LD+MCT: 85.50±3.7 / LD+LCT: 87.70±3.9 / CG: 86.80±6.5 (n=1 study record)	IG: 71.50±4.5 / CG: 71.60±4.2 (n=1 study record)	At risk (1 study record)	Confirmed (1 study record)	Abe 2016: 12 weeks	Osuka 2021: 12 weeks	Abe, 2016: Regular Exercise Activities in Nursing Homes	Osuka, 2021: No	Abe, 2016	Osuka, 2021
Leu+VD+LCT vs P	Leu+ VD+ LCT	P	LD+MCT: 85.50±3.7 / LD+LCT: 87.70±3.9 / CG: 86.80±6.5 (n=1 study record)	WP: 83.60±4.7 / P: 84.0±3.9 / CG: 83.70±5.1; IG: 76.80±6.2 / CG: 77.10±4.7; IG: 75.40±5.0 / CG: 76.70±5.8; IG: 72.60±5.1 / CG: 72.30±5.2;	At risk (1 study record)	Confirmed: 2; At risk: 3 study records	Abe 2016: 12 weeks	Björkman 2020: 48 weeks; Ottestad, 2017: 12 weeks; Alemán-Mateo, 2012: 12 weeks; Kao 2025: 12 weeks; Park, 2018: 12 weeks	Abe, 2016: Regular Exercise Activities in Nursing Homes	Björkman, 2020: Low-Intensity Home Workouts; Ottestad, 2017: No; Alemán-Mateo, 2012: No; Kao, 2025: No; Park, 2018: No	Abe, 2016	Björkman, 2020; Ottestad, 2017; Alemán-Mateo, 2012; Kao, 2025; Park, 2018

Indirect comparison	Node A	Node B	Age (A)	Age (B)	Sarcopenia status (A)	Sarcopenia status (B)	Intervention duration (A)	Intervention duration (B)	Background exercise (A)	Background exercise (B)	Supporting studies (A)	Supporting studies (B)
				WP: 76.80±3.70 / P: 77.30±3.67 / CG: 76.83±3.86 (n=5 study records)								
Leu+VD+LCT vs P+Ca	Leu+ VD+ LCT	P+Ca	LD+MCT: 85.50±3.7 / LD+LCT: 87.70±3.9 / CG: 86.80±6.5 (n=1 study record)	OG: 71(67,73) / GG: 75(70,77) / CG: 73(67,76) (n=1 study record)	At risk (1 study record)	At risk (1 study record)	Abe 2016: 12 weeks	Chen 2024: 12 weeks	Abe, 2016: Regular Exercise Activities in Nursing Homes	Chen, 2024: All were instructed to maintain their current level of physical activity	Abe, 2016	Chen, 2024
Leu+VD+LCT vs WP+CaHMB	Leu+ VD+ LCT	WP+CaHMB	LD+MCT: 85.50±3.7 / LD+LCT: 87.70±3.9 / CG: 86.80±6.5 (n=1 study record)	IG: 77(71,81) / CG: 77(71,81); IG: 77(71,81) / CG: 77(71,81) (n=2 study records)	At risk (1 study record)	Confirmed: 1; At risk: 1 study record	Abe 2016: 12 weeks	Cramer (sarcopenia subgroup) 2016: 24 weeks; Cramer (at-risk sarcopenia subgroup) 2016: 24 weeks	Abe, 2016: Regular Exercise Activities in Nursing Homes	Cramer (sarcopenia subgroup), 2016: No; Cramer (at-risk sarcopenia subgroup), 2016: No	Abe, 2016	Cramer (sarcopenia subgroup), 2016; Cramer (at-risk sarcopenia subgroup), 2016

Indirect comparison	Node A	Node B	Age (A)	Age (B)	Sarcopenia status (A)	Sarcopenia status (B)	Intervention duration (A)	Intervention duration (B)	Background exercise (A)	Background exercise (B)	Supporting studies (A)	Supporting studies (B)
Ca+HMB vs P	Ca+HMB	P	IG: 71.50±4.5 / CG: 71.60±4.2 (n=1 study record)	WP: 83.60±4.7 / P: 84.0±3.9 / CG: 83.70±5.1; IG: 76.80±6.2 / CG: 77.10±4.7; IG: 75.40±5.0 / CG: 76.70±5.8; IG: 72.60±5.1 / CG: 72.30±5.2; WP: 76.80±3.70 / P: 77.30±3.67 / CG: 76.83±3.86 (n=5 study records)	Confirmed (1 study record)	Confirmed: 2; At risk: 3 study records	Osuka 2021: 12 weeks	Björkman 2020: 48 weeks; Ottestad, 2017: 12 weeks; Alemán-Mateo, 2012: 12 weeks; Kao 2025: 12 weeks; Park, 2018: 12 weeks	Osuka, 2021: No	Björkman, 2020: Low-Intensity Home Workouts; Ottestad, 2017: No; Alemán-Mateo, 2012: No; Kao, 2025: No; Park, 2018: No	Osuka, 2021	Björkman, 2020; Ottestad, 2017; Alemán-Mateo, 2012; Kao, 2025; Park, 2018
Ca+HMB vs P+Ca	Ca+HMB	P+Ca	IG: 71.50±4.5 / CG: 71.60±4.2 (n=1 study record)	OG: 71(67,73) / GG: 75(70,77) / CG: 73(67,76) (n=1 study	Confirmed (1 study record)	At risk (1 study record)	Osuka 2021: 12 weeks	Chen 2024: 12 weeks	Osuka, 2021: No	Chen, 2024: All were instructed to maintain their current level of physical	Osuka, 2021	Chen, 2024

Indirect comparison	Node A	Node B	Age (A)	Age (B)	Sarcopenia status (A)	Sarcopenia status (B)	Intervention duration (A)	Intervention duration (B)	Background exercise (A)	Background exercise (B)	Supporting studies (A)	Supporting studies (B)
				record)						activity		
Ca+HMB vs WP+CaHMB	Ca+HMB	WP+CaHMB	IG: 71.50±4.5 / CG: 71.60±4.2 (n=1 study record)	IG: 77(71,81) / CG: 77(71,81); IG: 77(71,81) / CG: 77(71,81) (n=2 study records)	Confirmed (1 study record)	Confirmed: 1; At risk: 1 study record	Osuka 2021: 12 weeks	Cramer (sarcopenia subgroup) 2016: 24 weeks; Cramer (at-risk sarcopenia subgroup) 2016: 24 weeks	Osuka, 2021: No	Cramer (sarcopenia subgroup), 2016: No; Cramer (at-risk sarcopenia subgroup), 2016: No	Osuka, 2021	Cramer (sarcopenia subgroup), 2016; Cramer (at-risk sarcopenia subgroup), 2016
P vs P+Ca	P	P+Ca	WP: 83.60±4.7 / P: 84.0±3.9 / CG: 83.70±5.1; IG: 76.80±6.2 / CG: 77.10±4.7; IG: 75.40±5.0 / CG: 76.70±5.8; IG: 72.60±5.1 / CG: 72.30±5.2; WP: 76.80±3.	OG: 71(67,73) / GG: 75(70,77) / CG: 73(67,76) (n=1 study record)	Confirmed: 2; At risk: 3 study records	At risk (1 study record)	Björkman 2020: 48 weeks; Ottestad, 2017: 12 weeks; Alemán-Mateo, 2012: 12 weeks; Kao 2025: 12 weeks; Park, 2018: 12 weeks	Chen, 2024: 12 weeks	Björkman, 2020: Low-Intensity Home Workouts; Ottestad, 2017: No; Alemán-Mateo, 2012: No; Kao, 2025: No; Park, 2018: No	Chen, 2024: All were instructed to maintain their current level of physical activity	Björkman, 2020; Ottestad, 2017; Alemán-Mateo, 2012; Kao, 2025; Park, 2018	Chen, 2024

Indirect comparison	Node A	Node B	Age (A)	Age (B)	Sarcopenia status (A)	Sarcopenia status (B)	Intervention duration (A)	Intervention duration (B)	Background exercise (A)	Background exercise (B)	Supporting studies (A)	Supporting studies (B)
P vs WP+CaHMB	P	WP+CaHMB	70 / P: 77.30±3.67 / CG: 76.83±3.86 (n=5 study records) WP: 83.60±4.7 / P: 84.0±3.9 / CG: 83.70±5.1; IG: 76.80±6.2 / CG: 77.10±4.7; IG: 75.40±5.0 / CG: 76.70±5.8; IG: 72.60±5.1 / CG: 72.30±5.2; WP: 76.80±3.70 / P: 77.30±3.67 / CG: 76.83±3.86 (n=5	IG: 77(71,81) / CG: 77(71,81); IG: 77(71,81) / CG: 77(71,81) (n=2 study records)	Confirmed: 2; At risk: 3 study records	Confirmed: 1; At risk: 1 study record	Björkman 2020: 48 weeks; Ottestad, 2017: 12 weeks; Alemán-Mateo, 2012: 12 weeks; Kao 2025: 12 weeks; Park, 2018: 12 weeks	Cramer (sarcopenia subgroup) 2016: 24 weeks; Cramer (at-risk sarcopenia subgroup) 2016: 24 weeks	Björkman, 2020: Low-Intensity Home Workouts; Ottestad, 2017: No; Alemán-Mateo, 2012: No; Kao, 2025: No; Park, 2018: No	Cramer (sarcopenia subgroup), 2016: No; Cramer (at-risk sarcopenia subgroup), 2016: No	Björkman, 2020; Ottestad, 2017; Alemán-Mateo, 2012; Kao, 2025; Park, 2018	Cramer (sarcopenia subgroup), 2016; Cramer (at-risk sarcopenia subgroup), 2016

Indirect comparison	Node A	Node B	Age (A)	Age (B)	Sarcopenia status (A)	Sarcopenia status (B)	Intervention duration (A)	Intervention duration (B)	Background exercise (A)	Background exercise (B)	Supporting studies (A)	Supporting studies (B)
			study records)									
P+Ca vs WP+CaHMB	P+Ca	WP+CaHMB	OG: 71(67,73) / GG: 75(70,77) / CG: 73(67,76) (n=1 study record)	IG: 77(71,81) / CG: 77(71,81); IG: 77(71,81) / CG: 77(71,81) (n=2 study records)	At risk (1 study record)	Confirmed: 1; At risk: 1 study record	Chen 2024: 12 weeks	Cramer (sarcopenia subgroup) 2016: 24 weeks; Cramer (at-risk sarcopenia subgroup) 2016: 24 weeks	Chen, 2024: All were instructed to maintain their current level of physical activity	Cramer (sarcopenia subgroup), 2016: No; Cramer (at-risk sarcopenia subgroup), 2016: No	Chen, 2024	Cramer (sarcopenia subgroup), 2016; Cramer (at-risk sarcopenia subgroup), 2016

Supplementary Table S6. Distribution of potential effect modifiers across indirect comparisons for the five-times sit-to-stand test (5STS)

Indirect comparison	Node A	Node B	Age (A)	Age (B)	Sarcopenia status (A)	Sarcopenia status (B)	Intervention duration (A)	Intervention duration (B)	Background exercise (A)	Background exercise (B)	Supporting studies (A)	Supporting studies (B)
WP+VD+Leu vs WP+VD+VE	WP+VD+Leu	WP+VD+VE	IG: 72.50±5.57 / CG: 73.80±8.11; IG: 77.30±6.7 / CG: 78.10±7.0; IG: 81±7 / CG: 82±5; IG: 80.77±6.29 / CG: 80.21±8.54 (n=4 study records)	IG: 73.23±6.52 / CG: 74.83±5.94 (n=1 study record)	Confirmed (4 study records)	Confirmed (1 study record)	Lin 2021: 12 weeks; Bauer, 2015: 13 weeks; Rondanelli 2020: 6 weeks; Rondanelli 2016: 12 weeks	Bo, 2019: 24 weeks	Lin, 2021: No; Bauer, 2015: No; Rondanelli, 2020: Personalized physical rehabilitation therapy 5 times a week, 20–30 minutes per session; Rondanelli, 2016: Structured training 5 times a week, 20 minutes each session	Bo, 2019: No	Lin, 2021; Bauer, 2015; Rondanelli, 2020; Rondanelli, 2016	Bo, 2019
WP+VD+Leu vs P+Ca+CaP	WP+VD+Leu	P+Ca+CaP	IG: 72.50±5.57 / CG: 73.80±8.11; IG: 77.30±6.7 / CG: 78.10±7.	OG: 71(67,73) / GG: 75(70,77) / CG: 73(67,76) (n=1 study	Confirmed (4 study records)	At risk (1 study record)	Lin 2021: 12 weeks; Bauer, 2015: 13 weeks; Rondanelli 2020: 6 weeks; Rondanelli	Chen 2024: 12 weeks	Lin, 2021: No; Bauer, 2015: No; Rondanelli, 2020: Personalized physical rehabilitatio	Chen, 2024: All were instructed to maintain their current level of physical activity	Lin, 2021; Bauer, 2015; Rondanelli, 2020; Rondanelli, 2016	Chen, 2024

Indirect comparison	Node A	Node B	Age (A)	Age (B)	Sarcopenia status (A)	Sarcopenia status (B)	Intervention duration (A)	Intervention duration (B)	Background exercise (A)	Background exercise (B)	Supporting studies (A)	Supporting studies (B)
			0; IG: 81±7 / CG: 82±5; IG: 80.77±6.29 / CG: 80.21±8.54 (n=4 study records)	record)			2016: 12 weeks		n therapy 5 times a week, 20–30 minutes per session; Rondanelli, 2016: Structured training 5 times a week, 20 minutes each session			
WP+VD+Leu vs WP+Leu	WP+VD+Leu	WP+Leu	IG: 72.50±5.57 / CG: 73.80±8.11; IG: 77.30±6.7 / CG: 78.10±7.0; IG: 81±7 / CG: 82±5; IG: 80.77±6.29 / CG: 80.21±8.54 (n=4 study records)	LEU-PRO+n-3: 73±6 / LEU-PRO: 70±5 / CG: 73±7; IG: 68.80±6.5 / CG: 69.70±7.9 (n=2 study records)	Confirmed (4 study records)	At risk: 1; Confirmed: 1 study record	Lin 2021: 12 weeks; Bauer, 2015: 13 weeks; Rondanelli 2020: 6 weeks; Rondanelli 2016: 12 weeks	Murphy 2021: 24 weeks; Chung 2026: 12 weeks	Lin, 2021: No; Bauer, 2015: No; Rondanelli, 2020: Personalized physical rehabilitation therapy 5 times a week, 20–30 minutes per session; Rondanelli, 2016: Structured training 5 times a week, 20	Murphy, 2021: No; Chung, 2026: No	Lin, 2021; Bauer, 2015; Rondanelli, 2020; Rondanelli, 2016	Murphy, 2021; Chung, 2026

Indirect comparison	Node A	Node B	Age (A)	Age (B)	Sarcopenia status (A)	Sarcopenia status (B)	Intervention duration (A)	Intervention duration (B)	Background exercise (A)	Background exercise (B)	Supporting studies (A)	Supporting studies (B)
									minutes each session			
WP+VD+Leu vs WP+Leu+LCT	WP+VD+Leu	WP+Leu+LCT	IG: 72.50±5.57 / CG: 73.80±8.11; IG: 77.30±6.7 / CG: 78.10±7.0; IG: 81±7 / CG: 82±5; IG: 80.77±6.29 / CG: 80.21±8.54 (n=4 study records)	LEU-PRO+n-3: 73±6 / LEU-PRO: 70±5 / CG: 73±7 (n=1 study record)	Confirmed (4 study records)	At risk (1 study record)	Lin 2021: 12 weeks; Bauer, 2015: 13 weeks; Rondanelli 2020: 6 weeks; Rondanelli 2016: 12 weeks	Murphy 2021: 24 weeks	Lin, 2021: No; Bauer, 2015: No; Rondanelli, 2020: Personalized physical rehabilitation therapy 5 times a week, 20–30 minutes per session; Rondanelli, 2016: Structured training 5 times a week, 20 minutes per session	Murphy, 2021: No	Lin, 2021; Bauer, 2015; Rondanelli, 2020; Rondanelli, 2016	Murphy, 2021
WP+VD+Leu vs P	WP+VD+Leu	P	IG: 72.50±5.57 / CG: 73.80±8.11; IG: 77.30±6.7 / CG: 78.10±7.0; IG:	WP: 83.60±4.7 / P: 84.0±3.9 / CG: 83.70±5.1; IG: 76.80±6.2 / CG:	Confirmed (4 study records)	Confirmed: 2; At risk: 3 study records	Lin 2021: 12 weeks; Bauer, 2015: 13 weeks; Rondanelli 2020: 6 weeks; Rondanelli 2016: 12	Björkman 2020: 48 weeks; Ottestad, 2017: 12 weeks; Alemán-Mateo, 2012: 12 weeks;	Lin, 2021: No; Bauer, 2015: No; Rondanelli, 2020: Personalized physical rehabilitation therapy 5	Björkman, 2020: Low-Intensity Home Workouts; Ottestad, 2017: No; Alemán-Mateo, 2012:	Lin, 2021; Bauer, 2015; Rondanelli, 2020; Rondanelli, 2016	Björkman, 2020; Ottestad, 2017; Alemán-Mateo, 2012; Kao, 2025; Park, 2018

Indirect comparison	Node A	Node B	Age (A)	Age (B)	Sarcopenia status (A)	Sarcopenia status (B)	Intervention duration (A)	Intervention duration (B)	Background exercise (A)	Background exercise (B)	Supporting studies (A)	Supporting studies (B)
			81±7 / CG: 82±5; IG: 80.77±6.29 / CG: 80.21±8.54 (n=4 study records)	77.10±4.7; IG: 75.40±5.0 / CG: 76.70±5.8; IG: 72.60±5.1 / CG: 72.30±5.2; WP: 76.80±3.70 / P: 77.30±3.67 / CG: 76.83±3.86 (n=5 study records)			weeks	Kao 2025: 12 weeks; Park, 2018: 12 weeks	times a week, 20–30 minutes per session; Rondanelli, 2016: Structured training 5 times a week, 20 minutes per session	No; Kao, 2025: No; Park, 2018: No		
WP+VD+Leu vs P+Ca	WP+VD+Leu	P+Ca	IG: 72.50±5.57 / CG: 73.80±8.11; IG: 77.30±6.7 / CG: 78.10±7.0; IG: 81±7 / CG: 82±5; IG: 80.77±6.	OG: 71(67,73) / GG: 75(70,77) / CG: 73(67,76) (n=1 study record)	Confirmed (4 study records)	At risk (1 study record)	Lin 2021: 12 weeks; Bauer, 2015: 13 weeks; Rondanelli 2020: 6 weeks; Rondanelli 2016: 12 weeks	Chen 2024: 12 weeks	Lin, 2021: No; Bauer, 2015: No; Rondanelli, 2020: Personalized physical rehabilitation therapy 5 times a week, 20–30 minutes per session; Rondanelli,	Chen, 2024: All were instructed to maintain their current level of physical activity	Lin, 2021; Bauer, 2015; Rondanelli, 2020; Rondanelli, 2016	Chen, 2024

Indirect comparison	Node A	Node B	Age (A)	Age (B)	Sarcopenia status (A)	Sarcopenia status (B)	Intervention duration (A)	Intervention duration (B)	Background exercise (A)	Background exercise (B)	Supporting studies (A)	Supporting studies (B)
			29 / CG: 80.21±8.54 (n=4 study records)						2016: Structured training 5 times a week, 20 minutes each session			
WP+VD+VE vs P+Ca+CaP	WP+VD+VE	P+Ca+CaP	IG: 73.23±6.52 / CG: 74.83±5.94 (n=1 study record)	OG: 71(67,73) / GG: 75(70,77) / CG: 73(67,76) (n=1 study record)	Confirmed (1 study record)	At risk (1 study record)	Bo, 2019: 24 weeks	Chen, 2024: 12 weeks	Bo, 2019: No	Chen, 2024: All were instructed to maintain their current level of physical activity	Bo, 2019	Chen, 2024
WP+VD+VE vs WP+Leu	WP+VD+VE	WP+Leu	IG: 73.23±6.52 / CG: 74.83±5.94 (n=1 study record)	LEU-PRO+n-3: 73±6 / LEU-PRO: 70±5 / CG: 73±7; IG: 68.80±6.5 / CG: 69.70±7.9 (n=2 study records)	Confirmed (1 study record)	At risk: 1; Confirmed: 1 study record	Bo, 2019: 24 weeks	Murphy 2021: 24 weeks; Chung 2026: 12 weeks	Bo, 2019: No	Murphy, 2021: No; Chung, 2026: No	Bo, 2019	Murphy, 2021; Chung, 2026
WP+VD+VE	WP+V	WP+L	IG:	LEU-	Confirmed	At risk (1	Bo, 2019: 24	Murphy	Bo, 2019: No	Murphy,	Bo, 2019	Murphy,

Indirect comparison	Node A	Node B	Age (A)	Age (B)	Sarcopenia status (A)	Sarcopenia status (B)	Intervention duration (A)	Intervention duration (B)	Background exercise (A)	Background exercise (B)	Supporting studies (A)	Supporting studies (B)
vs WP+Leu+LCT	D+VE	eu+LCT	73.23±6.52 / CG: 74.83±5.94 (n=1 study record)	PRO+n-3: 73±6 / LEU-PRO: 70±5 / CG: 73±7 (n=1 study record)	(1 study record)	study record)	weeks	2021: 24 weeks		2021: No		2021
WP+VD+VE vs P	WP+VD+VE	P	IG: 73.23±6.52 / CG: 74.83±5.94 (n=1 study record)	WP: 83.60±4.7 / P: 84.0±3.9 / CG: 83.70±5.1; IG: 76.80±6.2 / CG: 77.10±4.7; IG: 75.40±5.0 / CG: 76.70±5.8; IG: 72.60±5.1 / CG: 72.30±5.2; WP: 76.80±3.70 / P: 77.30±3.	Confirmed (1 study record)	Confirmed: 2; At risk: 3 study records	Bo, 2019: 24 weeks	Björkman 2020: 48 weeks; Ottestad, 2017: 12 weeks; Alemán-Mateo, 2012: 12 weeks; Kao 2025: 12 weeks; Park, 2018: 12 weeks	Bo, 2019: No	Björkman, 2020: Low-Intensity Home Workouts; Ottestad, 2017: No; Alemán-Mateo, 2012: No; Kao, 2025: No; Park, 2018: No	Bo, 2019	Björkman, 2020; Ottestad, 2017; Alemán-Mateo, 2012; Kao, 2025; Park, 2018

Indirect comparison	Node A	Node B	Age (A)	Age (B)	Sarcopenia status (A)	Sarcopenia status (B)	Intervention duration (A)	Intervention duration (B)	Background exercise (A)	Background exercise (B)	Supporting studies (A)	Supporting studies (B)
WP+VD+VE vs P+Ca	WP+VD+VE	P+Ca	IG: 73.23±6.52 / CG: 74.83±5.94 (n=1 study record)	67 / CG: 76.83±3.86 (n=5 study records) OG: 71(67,73) / GG: 75(70,77) / CG: 73(67,76) (n=1 study record)	Confirmed (1 study record)	At risk (1 study record)	Bo, 2019: 24 weeks	Chen, 2024: 12 weeks	Bo, 2019: No	Chen, 2024: All were instructed to maintain their current level of physical activity	Bo, 2019	Chen, 2024
P+Ca+CaP vs WP+Leu	P+Ca+CaP	WP+Leu	OG: 71(67,73) / GG: 75(70,77) / CG: 73(67,76) (n=1 study record)	LEU-PRO+n-3: 73±6 / LEU-PRO: 70±5 / CG: 73±7; IG: 68.80±6.5 / CG: 69.70±7.9 (n=2 study records)	At risk (1 study record)	At risk: 1; Confirmed: 1 study record	Chen 2024: 12 weeks	Murphy 2021: 24 weeks; Chung 2026: 12 weeks	Chen, 2024: All were instructed to maintain their current level of physical activity	Murphy, 2021: No; Chung, 2026: No	Chen, 2024	Murphy, 2021; Chung, 2026
P+Ca+CaP vs WP+Leu+LCT	P+Ca+CaP	WP+Leu+LCT	OG: 71(67,73) / GG:	LEU-PRO+n-3: 73±6 /	At risk (1 study record)	At risk (1 study record)	Chen 2024: 12 weeks	Murphy 2021: 24 weeks	Chen, 2024: All were instructed to	Murphy, 2021: No	Chen, 2024	Murphy, 2021

Indirect comparison	Node A	Node B	Age (A)	Age (B)	Sarcopenia status (A)	Sarcopenia status (B)	Intervention duration (A)	Intervention duration (B)	Background exercise (A)	Background exercise (B)	Supporting studies (A)	Supporting studies (B)
			75(70,77) / CG: 73(67,76) (n=1 study record)	LEU-PRO: 70±5 / CG: 73±7 (n=1 study record)					maintain their current level of physical activity			
P+Ca+CaP vs P	P+Ca+CaP	P	OG: 71(67,73) / GG: 75(70,77) / CG: 73(67,76) (n=1 study record)	WP: 83.60±4.7 / P: 84.0±3.9 / CG: 83.70±5.1; IG: 76.80±6.2 / CG: 77.10±4.7; IG: 75.40±5.0 / CG: 76.70±5.8; IG: 72.60±5.1 / CG: 72.30±5.2; WP: 76.80±3.70 / P: 77.30±3.67 / CG: 76.83±3.	At risk (1 study record)	Confirmed: 2; At risk: 3 study records	Chen 2024: 12 weeks	Björkman 2020: 48 weeks; Ottestad, 2017: 12 weeks; Alemán-Mateo, 2012: 12 weeks; Kao 2025: 12 weeks; Park, 2018: 12 weeks	Chen, 2024: All were instructed to maintain their current level of physical activity	Björkman, 2020: Low-Intensity Home Workouts; Ottestad, 2017: No; Alemán-Mateo, 2012: No; Kao, 2025: No; Park, 2018: No	Chen, 2024	Björkman, 2020; Ottestad, 2017; Alemán-Mateo, 2012; Kao, 2025; Park, 2018

Indirect comparison	Node A	Node B	Age (A)	Age (B)	Sarcopenia status (A)	Sarcopenia status (B)	Intervention duration (A)	Intervention duration (B)	Background exercise (A)	Background exercise (B)	Supporting studies (A)	Supporting studies (B)
				86 (n=5 study records)								
WP+Leu vs P+Ca	WP+Leu	P+Ca	LEU-PRO+n-3: 73±6 / LEU-PRO: 70±5 / CG: 73±7; IG: 68.80±6.5 / CG: 69.70±7.9 (n=2 study records)	OG: 71(67,73) / GG: 75(70,77) / CG: 73(67,76) (n=1 study record)	At risk: 1; Confirmed: 1 study record	At risk (1 study record)	Murphy 2021: 24 weeks; Chung 2026: 12 weeks	Chen 2024: 12 weeks	Murphy, 2021: No; Chung, 2026: No	Chen, 2024: All were instructed to maintain their current level of physical activity	Murphy, 2021; Chung, 2026	Chen, 2024
WP+Leu+LCT vs P	WP+Leu+LCT	P	LEU-PRO+n-3: 73±6 / LEU-PRO: 70±5 / CG: 73±7 (n=1 study record)	WP: 83.60±4.7 / P: 84.0±3.9 / CG: 83.70±5.1; IG: 76.80±6.2 / CG: 77.10±4.7; IG: 75.40±5.0 / CG: 76.70±5.	At risk (1 study record)	Confirmed: 2; At risk: 3 study records	Murphy 2021: 24 weeks	Björkman 2020: 48 weeks; Ottestad, 2017: 12 weeks; Alemán-Mateo, 2012: 12 weeks; Kao 2025: 12 weeks; Park, 2018: 12 weeks	Murphy, 2021: No	Björkman, 2020: Low-Intensity Home Workouts; Ottestad, 2017: No; Alemán-Mateo, 2012: No; Kao, 2025: No; Park, 2018: No	Murphy, 2021	Björkman, 2020; Ottestad, 2017; Alemán-Mateo, 2012; Kao, 2025; Park, 2018

Indirect comparison	Node A	Node B	Age (A)	Age (B)	Sarcopenia status (A)	Sarcopenia status (B)	Intervention duration (A)	Intervention duration (B)	Background exercise (A)	Background exercise (B)	Supporting studies (A)	Supporting studies (B)
				8; IG: 72.60±5.1 / CG: 72.30±5.2; WP: 76.80±3.70 / P: 77.30±3.67 / CG: 76.83±3.86 (n=5 study records)								
WP+Leu+LCT vs P+Ca	WP+Leu+LCT	P+Ca	LEU-PRO+n-3: 73±6 / LEU-PRO: 70±5 / CG: 73±7 (n=1 study record)	OG: 71(67,73) / GG: 75(70,77) / CG: 73(67,76) (n=1 study record)	At risk (1 study record)	At risk (1 study record)	Murphy 2021: 24 weeks	Chen 2024: 12 weeks	Murphy, 2021: No	Chen, 2024: All were instructed to maintain their current level of physical activity	Murphy, 2021	Chen, 2024
P vs P+Ca	P	P+Ca	WP: 83.60±4.7 / P: 84.0±3.9 / CG: 83.70±5.1; IG: 76.80±6.	OG: 71(67,73) / GG: 75(70,77) / CG: 73(67,76) (n=1 study	Confirmed: 2; At risk: 3 study records	At risk (1 study record)	Björkman 2020: 48 weeks; Ottestad, 2017: 12 weeks; Alemán-Mateo, 2012:	Chen, 2024: 12 weeks	Björkman, 2020: Low-Intensity Home Workouts; Ottestad, 2017: No; Alemán-	Chen, 2024: All were instructed to maintain their current level of physical activity	Björkman, 2020; Ottestad, 2017; Alemán-Mateo, 2012; Kao, 2025; Park, 2018	Chen, 2024

Indirect comparison	Node A	Node B	Age (A)	Age (B)	Sarcopenia status (A)	Sarcopenia status (B)	Intervention duration (A)	Intervention duration (B)	Background exercise (A)	Background exercise (B)	Supporting studies (A)	Supporting studies (B)
			2 / CG: 77.10±4.7; IG: 75.40±5.0 / CG: 76.70±5.8; IG: 72.60±5.1 / CG: 72.30±5.2; WP: 76.80±3.70 / P: 77.30±3.67 / CG: 76.83±3.86 (n=5 study records)	record)			12 weeks; Kao 2025: 12 weeks; Park, 2018: 12 weeks		Mateo, 2012: No; Kao, 2025: No; Park, 2018: No			

Supplementary Table S7. Meta-regression analysis of the effect of intervention duration on grip strength (Pairwise meta-analysis)

Covariate	β regression coefficient	standard error	t	P>｜t｜	95%CI
Intervention duration	-0.0216184	0.011327	-1.91	0.072	(-0.0453259, 0.0020892)

Supplementary Table S8. Meta-regression analysis of the effect of intervention duration on gait speed (Pairwise meta-analysis)

Covariate	β regression coefficient	standard error	t	P>｜t｜	95%CI
Intervention duration	-0.0149045	0.0167049	-0.89	0.388	(-0.0509933, 0.0211843)

Supplementary Table S9. Leave-one-out sensitivity analysis of grip strength (pairwise meta-analysis)

Excluded comparison	Pooled SMD (95%CI)	P (Total)	I^2 (%)	P (I^2)
Original merge (unfiltered)	0.42(0.15, 0.68)	0.002	85%	＜0.00001
Murphy 2021 (WP+Leu)	0.43(0.15, 0.71)	0.003	85%	＜0.00001
Murphy 2021 (WP+Leu+LCT)	0.44(0.17, 0.72)	0.002	85%	＜0.00001
Lin 2021	0.44(0.16, 0.72)	0.002	85%	＜0.00001
El Hajj 2018	0.43(0.15, 0.72)	0.003	85%	＜0.00001
Martínez-Arnau 2020	0.43(0.15, 0.71)	0.002	85%	＜0.00001
Alemán-Mateo 2012	0.43(0.15, 0.71)	0.002	85%	＜0.00001
Chen 2024 (GG)	0.41(0.13, 0.69)	0.004	85%	＜0.00001
Chen 2024 (OG)	0.44(0.16, 0.71)	0.002	85%	＜0.00001
Bauer 2015	0.44(0.15, 0.73)	0.003	85%	＜0.00001
Rondanelli 2022	0.35(0.09, 0.61)	0.008	85%	＜0.00001
Rondanelli 2020	0.31(0.10, 0.52)	0.003	72%	＜0.00001
Rondanelli 2016	0.38(0.11, 0.64)	0.006	83%	＜0.00001
Björkman 2020 (WP)	0.44(0.16, 0.72)	0.002	85%	＜0.00001
Björkman 2020 (P)	0.44(0.16, 0.72)	0.002	85%	＜0.00001
Ottestad 2017	0.44(0.17, 0.72)	0.002	85%	＜0.00001
Flakoll 2004	0.40(0.13, 0.68)	0.004	85%	＜0.00001
Abe 2016 (Leu+VD+MCT)	0.38(0.11, 0.65)	0.006	85%	＜0.00001
Abe 2016 (Leu+VD+LCT)	0.41(0.14, 0.69)	0.003	85%	＜0.00001
Kao 2025	0.44(0.16, 0.72)	0.002	85%	＜0.00001
Bo 2019	0.40(0.12, 0.68)	0.005	85%	＜0.00001
Osuka 2021	0.43(0.15, 0.71)	0.003	85%	＜0.00001

Supplementary Table S10. Leave-one-out sensitivity analysis of gait speed (pairwise meta-analysis)

Excluded comparison	Pooled SMD (95%CI)	P (Total)	I^2 (%)	P (I^2)
Original merge (unfiltered)	0.27(0.08, 0.46)	0.006	65%	0.0002
Murphy 2021 (WP+Leu)	0.26(0.06, 0.46)	0.01	67%	＜0.0001
Murphy 2021 (WP+Leu+LCT)	0.25(0.05, 0.45)	0.01	66%	0.0002
Lin 2021	0.27(0.07, 0.47)	0.01	68%	＜0.0001
Cramer 2016 (sarcopenia subgroup)	0.31(0.12, 0.50)	0.001	60%	0.001
Cramer 2016 (at-risk sarcopenia subgroup)	0.27(0.05, 0.48)	0.01	67%	＜0.0001
Chen 2024 (GG)	0.28(0.08, 0.48)	0.006	67%	＜0.0001
Chen 2024 (OG)	0.28(0.08, 0.48)	0.006	67%	＜0.0001
Bauer 2015	0.28(0.06, 0.50)	0.01	67%	0.0001
Rondanelli 2020	0.16(0.05, 0.27)	**0.004**	**0%**	**0.46**
Abe 2016 (Leu+VD+MCT)	0.25(0.06, 0.44)	0.01	66%	0.0002
Abe 2016 (Leu+VD+LCT)	0.27(0.07, 0.47)	0.008	68%	＜0.0001
Kao 2025	0.28(0.07, 0.48)	0.008	67%	＜0.0001
Bo 2019	0.27(0.06, 0.47)	0.01	68%	＜0.0001
Park 2018 (WP)	0.28(0.08, 0.49)	0.007	67%	＜0.0001
Park 2018 (P)	0.29(0.09, 0.49)	0.004	66%	0.0002
Osuka 2021	0.28(0.08, 0.49)	0.006	67%	0.0001

Supplementary Table S11. Leave-one-out sensitivity analysis of 5STS (pairwise meta-analysis)

Excluded comparison	Pooled SMD (95%CI)	P (Total)	I^2 (%)	P (I^2)
Original merge (unfiltered)	-0.31(-0.81, 0.20)	0.23	91%	＜0.00001
Murphy 2021 (WP+Leu)	-0.38(-0.92, 0.16)	0.17	92%	＜0.00001
Murphy 2021 (WP+Leu+LCT)	-0.40(-0.93, 0.13)	0.14	91%	＜0.00001
Chen 2024 (GG)	-0.31(-0.86, 0.25)	0.28	92%	＜0.00001
Chen 2024 (OG)	-0.34(-0.89, 0.21)	0.23	92%	＜0.00001
Bauer 2015	-0.29(-0.93, 0.34)	0.37	92%	＜0.00001
Rondanelli 2020	-0.08(-0.32, 0.15)	**0.48**	**51%**	**0.04**
Ottestad 2017	-0.34(-0.89, 0.20)	0.22	92%	＜0.00001
Kao 2025	-0.35(-0.92, 0.22)	0.22	92%	＜0.00001
Chung 2026	-0.29(-0.85, 0.26)	0.30	92%	＜0.00001
Bo 2019	-0.31(-0.87, 0.26)	0.28	92%	＜0.00001

Supplementary Table S12. Comparison of pooled effect estimates between the primary analysis and the broader-node sensitivity analysis (pairwise meta-analysis)

Outcomes	Primary Analysis		Broader-node sensitivity analysis	
	SMD (95% CI)	I^2(%)	SMD (95% CI)	I^2(%)
ASMI	0.25(0.10, 0.40)	0%	0.25(0.10, 0.41)	0%
Grip strength	0.42(0.15, 0.68)	85%	0.47(0.15, 0.80)	87.7%
Gait speed	0.27(0.08, 0.46)	65%	0.19(0.05, 0.33)	7%
5STS	-0.31(-0.81, 0.20)	91%	-0.41(-1.06, 0.24)	93.7%

Supplementary Table S13. Population-restriction sensitivity analysis of pairwise meta-analysis restricted to participants with confirmed sarcopenia (pairwise meta-analysis)

Outcome	Analysis	SMD (95%CI)	P	I^2 (%)
ASMI	Primary analysis	0.25(0.10, 0.40)	0.001	0
	Confirmed sarcopenia only	0.31(0.13, 0.49)	< 0.001	0
Grip strength	Primary analysis	0.42(0.15, 0.68)	0.002	85%
	Confirmed sarcopenia only	0.54(0.11, 0.96)	0.01	91%
Gait speed	Primary analysis	0.27(0.08, 0.46)	0.006	65%
	Confirmed sarcopenia only	0.30(-0.11, 0.71)	0.15	86%

5STS	Primary analysis	-0.31(-0.81, 0.20)	0.23	91%
	Confirmed sarcopenia only	-0.90(-1.88, 0.07)	0.07	95%

Notes: The population-restriction sensitivity analysis included only studies or study subgroups enrolling participants with confirmed sarcopenia and excluded populations identified only as having probable sarcopenia, low muscle mass, or another high-risk condition without a confirmed diagnosis. Effect estimates were calculated using the same random-effects models as in the primary pairwise meta-analyses. For ASMI, grip strength, and gait speed, a positive SMD indicates a favorable effect of nutritional supplementation; for 5STS, a negative SMD indicates a favorable effect. ASMI, appendicular skeletal muscle mass index; 5STS, five-times sit-to-stand test; SMD, standardized mean difference; CI, confidence interval.

Supplementary Table S14. Sensitivity analysis excluding single-study intervention nodes for appendicular skeletal muscle mass index (ASMI)

Remaining interventions	Before removal		After removal	
	SMD (95%CI)	SUCRA	SMD (95%CI)	SUCRA
WP+VD+Leu	0.29(0.06, 0.52)	60.8%	0.29(0.05, 0.54)	89.7%
P	0.14(-0.19, 0.47)	38.5%	0.14(-0.26, 0.54)	50.2%

Supplementary Table S15. Sensitivity analysis excluding single-study intervention nodes for grip strength

Remaining interventions	Before removal		After removal	
	SMD (95%CI)	SUCRA	SMD (95%CI)	SUCRA
WP+VD+Leu	0.82(0.13, 1.50)	69.9%	0.83(0.08, 1.58)	94.2%
P	0.16(-0.47, 0.80)	39.1%	0.22(-0.56, 0.99)	40.5%

Supplementary Table S16. Sensitivity analysis excluding single-study intervention nodes for gait speed

Remaining interventions	Before removal		After removal	
	SMD (95%CI)	SUCRA	SMD (95%CI)	SUCRA
WP+VD+Leu	0.17(-0.25, 0.60)	48.7%	0.17(-0.51, 0.84)	61.5%
WP+CaHMB	0.16(-0.53, 0.86)	47.4%	0.17(-0.75,1.09)	59.7%
WP	0.11(-0.47, 0.69)	46.2%	0.11(-0.96, 1.17)	34.5%

Supplementary Table S17. Sensitivity analysis excluding single-study intervention nodes for five-times sit-to-stand test (5STS)

Remaining interventions	Before removal		After removal	
	SMD (95%CI)	SUCRA	SMD (95%CI)	SUCRA
WP+VD+Leu	-1.41(-2.63, -0.18)	91.6%	-1.41(-2.64, -0.18)	96.6%
WP+Leu	0.04(-1.35, 1.42)	43.3%	0.04(-1.35, 1.42)	37.1%
P	0.19(-0.94, 1.31)	34.6%	0.19(-0.94, 1.31)	27.4%

Supplementary Table S18. Trial-level leave-one-out network meta-analysis for appendicular skeletal muscle mass index (ASMI)

WP+VD+Leu VS. Control		
**Excluded comparison**	**Pooled SMD (95%CI)**	**P**
Original NMA	0.29(0.06, 0.52)	0.013
Lin 2021	0.29(0.05, 0.54)	0.020
Rondanelli 2020	0.20(-0.11, 0.51)	0.201
Rondanelli 2016	0.39(0.08, 0.70)	0.014

P VS. Control		
**Excluded comparison**	**Pooled SMD (95%CI)**	**P**
Original NMA	0.14(-0.19, 0.47)	0.398
Lin 2021	0.14(-0.26, 0.54)	0.486
Chung 2026	0.14(-0.19, 0.47)	0.398
Kao 2025	0.14(-0.49, 0.72)	0.632

Supplementary Table S19. Trial-level leave-one-out network meta-analysis for grip strength

WP+VD+Leu VS. Control		
**Excluded comparison**	**Pooled SMD (95%CI)**	**P**
Original NMA	0.82(0.13, 1.50)	0.020
Lin 2021	1.03(0.31, 1.76)	0.005
Bauer 2015	1.11(0.37, 1.84)	0.003
Rondanelli 2020	0.40(-0.08, 0.88)	0.106
Rondanelli 2016	0.72(-0.14, 1.59)	0.102

P VS. Control		
**Excluded comparison**	**Pooled SMD (95%CI)**	**P**
Original NMA	0.16(-0.47, 0.80)	0.618
Lin 2021	-0.02(-0.68, 0.64)	0.959
Björkman 2020	0.20(-0.59, 0.98)	0.624
Ottestad 2017	0.25(-0.50, 1.00)	0.510
Alemán-Mateo 2012	0.17(-0.60, 0.94)	0.662
Kao 2025	0.22(-0.56, 0.99)	0.586

Supplementary Table S20. Trial-level leave-one-out network meta-analysis for gait speed

WP+VD+Leu VS. Control		
**Excluded comparison**	**Pooled SMD (95%CI)**	**P**
Original NMA	0.17(-0.25, 0.60)	0.424
Lin 2021	0.17(-0.51, 0.84)	0.628
Bauer 2015	0.20(-0.92, 1.33)	0.723

P VS. Control

Excluded comparison	Pooled SMD (95%CI)	P
Original NMA	-0.08(-0.58, 0.41)	0.746
Lin 2021	-0.07(-0.84, 0.70)	0.862
Park 2018	—	—

WP+CaHMB VS. Control

Excluded comparison	Pooled SMD (95%CI)	P
Original NMA	0.16(-0.53, 0.86)	0.636
Cramer 2016	—	—

WP VS. Control

Excluded comparison	Pooled SMD (95%CI)	P
Original NMA	0.11(-0.47, 0.69)	0.706
Cramer 2016	—	—
Park 2018	—	—

Supplementary Table S21. Trial-level leave-one-out network meta-analysis for five-times sit-to-stand test (5STS)

WP+VD+Leu VS. Control

Excluded comparison	Pooled SMD (95%CI)	P
Original NMA	-1.41(-2.63, -0.18)	0.025
Bauer 2015	-2.45(-3.00, -1.91)	< 0.001
Rondanelli 2020	-0.41(−0.79, −0.03)	0.033

WP+Leu VS. Control

Excluded comparison	Pooled SMD (95%CI)	P
Original NMA	0.04(−1.35, 1.42)	0.985
Murphy 2021	−0.40(−2.93, 2.13)	0.755
Chung 2026	0.33(−1.73, 2.40)	0.756

P VS. Control

Excluded comparison	Pooled SMD (95%CI)	P
Original NMA	0.19(−0.94, 1.31)	0.744
Ottestad 2017	0.29(−1.43, 2.02)	0.737
Chung 2026	0.04(−1.41, 1.50)	0.954
Kao 2025	0.28(−1.49, 2.04)	0.761

Supplementary Table S22. Alternative classification of intervention nodes used in the sensitivity analysis

Broader node	Original formulation-specific intervention nodes
Control	nodes assigned to the Control node in the primary analysis
Protein-based interventions	P, WP, P+Ca, P+Ca+CaP, WP+Leu, WP+Leu+LCT, WP + CaHMB
Protein + vitamin D-based interventions	WP+VD+Leu, WP+VD+VE
Other nutritional interventions	Leu+VD+MCT, Leu+VD+LCT, HMB+ARG+LYS, Ca+HMB, ω-3+Leu+PS23, Leu, VD

Supplementary Table S23. Network meta-analysis results using the alternative node classification

Outcome	Comparison	SMD (95% CI)
ASMI	Protein-based interventions vs. Control	0.16 (−0.17, 0.48)
	Protein + vitamin D-based interventions vs. Control	**0.33 (0.12, 0.54)**
	Other nutritional interventions vs. Control	0.27 (−0.04, 0.57)
	Protein + vitamin D-based interventions vs. Protein-based interventions	0.17 (−0.17, 0.52)
	Other nutritional interventions vs. Protein-based interventions	0.11 (−0.34, 0.56)
	Protein + vitamin D-based interventions vs. Other nutritional interventions	0.06 (−0.31, 0.43)
Grip strength	Protein-based interventions vs. Control	0.14 (−0.32, 0.60)
	Protein + vitamin D-based interventions vs. Control	**0.80 (0.27, 1.32)**
	Other nutritional interventions vs. Control	**0.62 (0.11, 1.13)**
	Protein + vitamin D-based interventions vs. Protein-based interventions	**0.65 (0.02, 1.29)**
	Other nutritional interventions vs. Protein-based interventions	0.48 (−0.21, 1.16)
	Protein + vitamin D-based interventions vs. Other nutritional interventions	0.18 (−0.55, 0.91)
Gait speed	Protein-based interventions vs. Control	0.13 (−0.11, 0.36)
	Protein + vitamin D-based interventions vs. Control	**0.22 (0.02, 0.41)**
	Other nutritional interventions vs. Control	0.20 (−0.21, 0.60)
	Protein + vitamin D-based interventions vs. Protein-based interventions	0.09 (−0.19, 0.37)
	Other nutritional interventions vs. Protein-based interventions	0.07 (−0.40, 0.54)
	Protein + vitamin D-based	0.02 (−0.43, 0.47)

interventions vs. Other nutritional interventions

Supplementary Table S24. Population-restriction sensitivity analysis of the network meta-analysis for ASMI

Comparison	Primary NMA SMD (95% CI)	Confirmed-sarcopenia-only NMA SMD (95% CI)
P vs Control	0.14 (−0.19, 0.47)	0.14 (−0.44, 0.72)
Ca+HMB vs Control	0.07 (−0.39, 0.52)	0.07 (−0.39, 0.52)
Ca+HMB vs P	−0.08 (−0.64, 0.48)	−0.08 (−0.81, 0.66)
WP+Leu vs Control	0.14 (−0.52, 0.80)	0.14 (−0.67, 0.96)
WP+Leu vs P	0.00 (−0.57, 0.57)	0.00 (−0.57, 0.57)
WP+Leu vs Ca+HMB	0.08 (−0.73, 0.88)	0.08 (−0.86, 1.01)
Leu vs Control	0.18 (−0.42, 0.78)	0.18 (−0.42, 0.78)
Leu vs P	0.04 (−0.65, 0.72)	0.04 (−0.80, 0.87)
Leu vs Ca+HMB	0.11 (−0.64, 0.86)	0.11 (−0.64, 0.86)
Leu vs WP+Leu	0.04 (−0.85, 0.93)	0.04 (−0.97, 1.05)
WP+VD+Leu vs Control	**0.29 (0.06, 0.52)**	**0.29 (0.05, 0.54)**
WP+VD+Leu vs P	0.15 (−0.20, 0.50)	0.15 (−0.37, 0.68)
WP+VD+Leu vs Ca+HMB	0.23 (−0.28, 0.74)	0.23 (−0.29, 0.74)
WP+VD+Leu vs WP+Leu	0.15 (−0.52, 0.82)	0.15 (−0.63, 0.93)
WP+VD+Leu vs Leu	0.11 (−0.53, 0.76)	0.11 (−0.53, 0.76)
ω-3+Leu+PS23 vs Control	**0.67 (0.10, 1.25)**	**0.67 (0.10, 1.25)**
ω-3+Leu+PS23 vs P	0.53 (−0.13, 1.19)	0.53 (−0.28, 1.35)
ω-3+Leu+PS23 vs Ca+HMB	0.61 (−0.12, 1.34)	0.61 (−0.12, 1.34)
ω-3+Leu+PS23 vs WP+Leu	0.53 (−0.34, 1.41)	0.53 (−0.47, 1.53)
ω-3+Leu+PS23 vs Leu	0.49 (−0.34, 1.32)	0.49 (−0.34, 1.32)
ω-3+Leu+PS23 vs WP+VD+Leu	0.38 (−0.24, 1.00)	0.38 (−0.24, 1.01)
WP+VD+VE vs Control	0.52 (0.00, 1.03)	0.52 (0.00, 1.03)
WP+VD+VE vs P	0.37 (−0.24, 0.98)	0.37 (−0.40, 1.15)
WP+VD+VE vs Ca+HMB	0.45 (−0.24, 1.14)	0.45 (−0.24, 1.13)
WP+VD+VE vs WP+Leu	0.37 (−0.46, 1.21)	0.37 (−0.59, 1.34)
WP+VD+VE vs Leu	0.34 (−0.45, 1.13)	0.34 (−0.45, 1.13)
WP+VD+VE vs WP+VD+Leu	0.22 (−0.34, 0.79)	0.22 (−0.35, 0.79)
WP+VD+VE vs ω-3+Leu+PS23	−0.16 (−0.93, 0.61)	−0.16 (−0.93, 0.61)

Supplementary Table S25. Population-restriction sensitivity analysis of the network meta-analysis for grip strength

Comparison	Primary NMA SMD (95% CI)	Confirmed-sarcopenia-only NMA SMD (95% CI)
WP+VD+VE vs Control	0.73 (−0.65, 2.12)	0.73 (−1.03, 2.50)
ω-3+Leu+PS23 vs Control	**1.84 (0.39, 3.28)**	**1.84 (0.02, 3.65)**
WP+VD+Leu vs Control	**0.82 (0.13, 1.50)**	0.91 (−0.01, 1.84)
WP vs Control	0.06 (−1.14, 1.25)	0.49 (−1.70, 2.67)
Leu vs Control	0.11 (−1.31, 1.52)	0.11 (−1.68, 1.90)
Ca+HMB vs Control	0.17 (−1.19, 1.53)	0.17 (−1.58, 1.91)
P vs Control	0.16 (−0.47, 0.80)	0.55 (−0.79, 1.90)
ω-3+Leu+PS23 vs WP+VD+VE	1.11 (−0.90, 3.11)	1.11 (−1.43, 3.64)
WP+VD+Leu vs WP+VD+VE	0.08 (−1.46, 1.63)	0.18 (−1.81, 2.17)
WP vs WP+VD+VE	−0.67 (−2.50, 1.15)	−0.24 (−3.05, 2.57)
Leu vs WP+VD+VE	−0.63 (−2.61, 1.35)	−0.63 (−3.14, 1.89)
Ca+HMB vs WP+VD+VE	−0.57 (−2.51, 1.37)	−0.57 (−3.05, 1.92)
P vs WP+VD+VE	−0.57 (−2.09, 0.95)	−0.18 (−2.40, 2.04)

WP+VD+Leu vs ω-3+Leu+PS23	−1.02 (−2.62, 0.58)	−0.92 (−2.96, 1.11)
WP vs ω-3+Leu+PS23	−1.78 (−3.66, 0.10)	−1.35 (−4.19, 1.49)
Leu vs ω-3+Leu+PS23	−1.73 (−3.75, 0.29)	−1.73 (−4.28, 0.82)
Ca+HMB vs ω-3+Leu+PS23	−1.67 (−3.66, 0.31)	−1.67 (−4.19, 0.85)
P vs ω-3+Leu+PS23	**−1.68 (−3.26, −0.10)**	−1.28 (−3.54, 0.98)
WP vs WP+VD+Leu	−0.76 (−2.10, 0.58)	−0.43 (−2.61, 1.76)
Leu vs WP+VD+Leu	−0.71 (−2.28, 0.06)	−0.81 (−2.82, 1.21)
Ca+HMB vs WP+VD+Leu	−0.65 (−2.17, 0.87)	−0.75 (−2.72, 1.23)
P vs WP+VD+Leu	−0.66 (−1.48, 0.17)	−0.36 (−1.70, 0.98)
Leu vs WP	0.05 (−1.80, 1.90)	−0.38 (−3.21, 2.44)
Ca+HMB vs WP	0.11 (−1.70, 1.92)	−0.32 (−3.12, 2.48)
P vs WP	0.10 (−1.09, 1.30)	0.07 (−1.66, 1.79)
Ca+HMB vs Leu	0.06 (−1.90, 2.02)	0.06 (−2.44, 2.56)
P vs Leu	0.06 (−1.49, 1.61)	0.45 (−1.79, 2.69)
P vs Ca+HMB	−0.01 (−1.50, 1.49)	0.39 (−1.82, 2.59)

Supplementary Table S26. Population-restriction sensitivity analysis of the network meta-analysis for gait speed

Comparison	Primary NMA SMD (95% CI)	Confirmed-sarcopenia-only NMA SMD (95% CI)
P vs Control	−0.08 (−0.58, 0.41)	−0.11 (−0.68, 0.47)
Ca+HMB vs Control	0.06 (−0.56, 0.68)	0.06 (−0.39, 0.51)
Ca+HMB vs P	0.14 (−0.65, 0.94)	0.17 (−0.56, 0.90)
WP+VD+Leu vs Control	0.17 (−0.25, 0.60)	0.17 (−0.06, 0.39)
WP+VD+Leu vs P	0.26 (−0.26, 0.77)	0.27 (−0.25, 0.80)
WP+VD+Leu vs Ca+HMB	0.11 (−0.64, 0.87)	0.11 (−0.40, 0.61)
WP+VD+VE vs Control	0.30 (−0.37, 0.96)	0.30 (−0.21, 0.80)
WP+VD+VE vs P	0.38 (−0.45, 1.21)	0.40 (−0.36, 1.17)
WP+VD+VE vs Ca+HMB	0.24 (−0.67, 1.15)	0.24 (−0.45, 0.92)
WP+VD+VE vs WP+VD+Leu	0.12 (−0.67, 0.91)	0.13 (−0.43, 0.69)

Supplementary Table S27. Population-restriction sensitivity analysis of the network meta-analysis for 5STS

Comparison	Primary NMA SMD (95% CI)	Confirmed-sarcopenia-only NMA SMD (95% CI)
WP+VD+VE vs Control	−0.30 (−2.07, 1.47)	−0.30 (−3.15, 2.55)
WP+VD+Leu vs Control	**−1.41 (−2.63, −0.18)**	−1.42 (−3.42, 0.58)
WP+VD+Leu vs WP+VD+VE	−1.10 (−3.26, 1.05)	−1.12 (−4.60, 2.36)
WP+Leu vs P	−0.15 (−1.54, 1.23)	−0.44 (−3.31, 2.42)

Supplementary Table S28. Summary of findings and certainty of evidence for principal intervention–outcome comparisons

Outcome	Comparison	NMA SMD (95% CI)	Evidence base	Within-study bias	Reporting bias	Indirectness	Imprecision	Heterogeneity	Incoherence	Overall confidence
ASMI	WP+VD+Leu vs. Control	0.29 (0.06, 0.52)	3 studies	Some concerns	No concerns	No concerns	No concerns	Some concerns	No concerns	Low
ASMI	ω-3+Leu+PS23 vs. Control	0.67 (0.10, 1.25)	1 study (single-study node)	Some concerns	No concerns	No concerns	No concerns	Major concerns	No concerns	Very low
Grip strength	WP+VD+Leu vs. Control	0.82 (0.13, 1.50)	4 studies	Some concerns	Some concerns	No concerns	No concerns	Some concerns	No concerns	Very low
Grip strength	ω-3+Leu+PS23 vs. Control	1.84 (0.39, 3.28)	1 study (single-study node)	Some concerns	No concerns	No concerns	No concerns	Major concerns	No concerns	Very low
Gait speed	Leu+VD+MCT vs. Control	1.28 (0.13, 2.43)	1 study (single-study node)	Some concerns	No concerns	No concerns	Major concerns	No concerns	No concerns	Very low
Gait speed	Leu+VD+MCT vs. P	1.37 (0.11, 2.62)	1 study (single-study node)	Some concerns	No concerns	No concerns	Major concerns	No concerns	No concerns	Very low
5STS	WP+VD+Leu vs. Control	−1.41 (−2.63, −0.18)	2 studies	Some concerns	No concerns	No concerns	No concerns	Some concerns	No concerns	Low

Notes: Effect estimates are presented as standardized mean differences (SMDs) with 95% confidence intervals (CIs). Positive SMDs indicate improvement in ASMI, grip strength, and gait speed, whereas negative SMDs indicate improvement in 5STS performance. Certainty of evidence was assessed using the CINeMA framework across six domains: within-study bias, reporting bias, indirectness, imprecision, heterogeneity, and incoherence. The table summarizes principal intervention–outcome comparisons, whereas complete CINeMA assessments for all network comparisons are provided in the Supplementary Material. Single-study nodes comparisons are explicitly identified to distinguish findings based on limited evidence from those supported by replicated evidence.

Supplementary Table S29. Certainty of evidence for each pairwise comparison of nutritional strategies on appendicular skeletal muscle mass index (ASMI) in older adults using the CINeMA framework

Comparison	Quality assessment						Confidence rating	Reason(s) for downgrading
	Within-study bias	Reporting bias	Indirectness	Imprecision	Heterogeneity	Incoherence		
Control VS WP+VD+VE	Some concerns	No concerns	No concerns	No concerns	Major concerns	No concerns	Very low	①⑤
Control VS ω-3+Leu+PS23	Some concerns	No concerns	No concerns	No concerns	Major concerns	No concerns	Very low	①⑤
Control VS WP+VD+Leu	Some concerns	No concerns	No concerns	No concerns	Some concerns	No concerns	Low	①⑤
Control VS Leu	No concerns	No concerns	No concerns	Major concerns	No concerns	No concerns	Low	④
Control VS Ca+HMB	No concerns	No concerns	No concerns	Major concerns	No concerns	No concerns	Low	④
Control VS P	Some concerns	No concerns	No concerns	Some concerns	Some concerns	No concerns	Very low	①④⑤
P VS WP+VD+Leu	Some concerns	No concerns	No concerns	Some concerns	Some concerns	No concerns	Very low	①④⑤
P VS WP+Leu	Some concerns	No concerns	No concerns	Major concerns	No concerns	No concerns	Very low	①④
Control VS WP+Leu	Some concerns	No concerns	No concerns	Major concerns	No concerns	No concerns	Very low	①④

Comparison	Quality assessment						Confidence rating	Reason(s) for downgrading
	Within-study bias	Reporting bias	Indirectness	Imprecision	Heterogeneity	Incoherence		
WP+VD+VE VS ω-3+Leu+PS23	Some concerns	No concerns	No concerns	Major concerns	No concerns	No concerns	Very low	①④
WP+VD+VE VS WP+VD+Leu	Some concerns	No concerns	No concerns	Major concerns	No concerns	No concerns	Very low	①④
WP+VD+VE VS Leu	Some concerns	No concerns	No concerns	Major concerns	No concerns	No concerns	Very low	①④
WP+VD+VE VS WP+Leu	Some concerns	No concerns	No concerns	Major concerns	No concerns	No concerns	Very low	①④
Ca+HMB VS WP+VD+VE	Some concerns	No concerns	No concerns	Some concerns	Some concerns	No concerns	Very low	①④⑤
P VS WP+VD+VE	Some concerns	No concerns	No concerns	Some concerns	Some concerns	No concerns	Very low	①④⑤
ω-3+Leu+PS23 VS WP+VD+Leu	Some concerns	No concerns	Some concerns	Some concerns	Some concerns	No concerns	Very low	①③④⑤
ω-3+Leu+PS23 VS Leu	Some concerns	No concerns	No concerns	Major concerns	No concerns	No concerns	Very low	①④
ω-3+Leu+PS23 VS WP+Leu	Some concerns	No concerns	No concerns	Major concerns	No concerns	No concerns	Very low	①④
Ca+HMB VS ω-3+Leu+PS23	Some concerns	No concerns	No concerns	Some concerns	Some concerns	No concerns	Very low	①④⑤
P	Some concerns	No concerns	Some concerns	Some concerns	Some concerns	No concerns	Very low	①③④⑤

Comparison	Quality assessment						Confidence rating	Reason(s) for downgrading
	Within-study bias	Reporting bias	Indirectness	Imprecision	Heterogeneity	Incoherence		
VS ω-3+Leu+PS23								
WP+VD+Leu VS Leu	No concerns	No concerns	No concerns	Major concerns	No concerns	No concerns	Low	④
WP+VD+Leu VS WP+Leu	Some concerns	No concerns	No concerns	Major concerns	No concerns	No concerns	Very low	①④
Ca+HMB VS WP+VD+Leu	No concerns	No concerns	No concerns	Some concerns	Some concerns	No concerns	Low	④⑤
Leu VS WP+Leu	Some concerns	No concerns	No concerns	Major concerns	No concerns	No concerns	Very low	①④
Ca+HMB VS Leu	No concerns	No concerns	No concerns	Major concerns	No concerns	No concerns	Low	④
P VS Leu	Some concerns	No concerns	No concerns	Major concerns	No concerns	No concerns	Very low	①④
Ca+HMB VS WP+Leu	Some concerns	No concerns	No concerns	Major concerns	No concerns	No concerns	Very low	①④
Ca+HMB VS P	Some concerns	No concerns	No concerns	Major concerns	No concerns	No concerns	Very low	①④

Notes: ① within-study bias; ② reporting bias; ③ indirectness; ④ imprecision; ⑤ heterogeneity; ⑥ incoherence.

Supplementary Table S30. Certainty of evidence for each pairwise comparison of nutritional strategies on grip strength in older adults using the CINeMA framework

Comparison	Quality assessment						Confidence rating	Reason(s) for downgrading
	Within-study bias	Reporting bias	Indirectness	Imprecision	Heterogeneity	Incoherence		
Control VS WP+VD+VE	Some concerns	No concerns	No concerns	No concerns	Major concerns	No concerns	Very low	①⑤
Control VS ω-3+Leu+PS23	Some concerns	No concerns	No concerns	No concerns	Major concerns	No concerns	Very low	①⑤
Control VS WP+VD+Leu	Some concerns	Some concerns	No concerns	No concerns	Some concerns	No concerns	Very low	①②⑤
Control VS WP	Some concerns	No concerns	No concerns	Major concerns	No concerns	No concerns	Very low	①④
Control VS Leu	No concerns	No concerns	No concerns	Major concerns	No concerns	No concerns	Low	④
Control VS VD	Some concerns	No concerns	No concerns	No concerns	Major concerns	No concerns	Very low	①⑤
Control VS Ca+HMB	No concerns	No concerns	No concerns	Major concerns	No concerns	No concerns	Low	④
Control VS P+Ca+CaP	Some concerns	No concerns	No concerns	No concerns	Major concerns	No concerns	Very low	①⑤
Control VS WP+Leu	Some concerns	No concerns	No concerns	Some concerns	Some concerns	No concerns	Very low	①④⑤

Comparison	Quality assessment						Confidence rating	Reason(s) for downgrading
	Within-study bias	Reporting bias	Indirectness	Imprecision	Heterogeneity	Incoherence		
Control VS WP+Leu+LCT	Some concerns	No concerns	No concerns	Some concerns	Some concerns	No concerns	Very low	①④⑤
Control VS Leu+VD+MCT	Some concerns	No concerns	No concerns	Major concerns	No concerns	No concerns	Very low	①④
Control VS Leu+VD+ LCT	Some concerns	No concerns	No concerns	Major concerns	No concerns	No concerns	Very low	①④
Control VS HMB+ARG+LYS	Some concerns	No concerns	No concerns	Major concerns	Some concerns	No concerns	Very low	①④⑤
Control VS P	Some concerns	No concerns	No concerns	Major concerns	No concerns	No concerns	Very low	①④
Control VS P+Ca	Some concerns	No concerns	No concerns	Major concerns	No concerns	No concerns	Very low	①④
WP+VD+VE VS ω-3+Leu+PS23	Some concerns	No concerns	No concerns	Some concerns	Some concerns	No concerns	Very low	①④⑤
WP+VD+VE VS WP+VD+Leu	Some concerns	No concerns	No concerns	Some concerns	Some concerns	No concerns	Very low	①④⑤
WP+VD+VE VS WP	Some concerns	No concerns	Some concerns	Some concerns	Some concerns	No concerns	Very low	①③④⑤
WP+VD+VE VS Leu	Some concerns	No concerns	No concerns	Major concerns	No concerns	No concerns	Very low	①④
WP+VD+VE VS	Some concerns	No concerns	No concerns	Major concerns	No concerns	No concerns	Very low	①④

Comparison	Quality assessment						Confidence rating	Reason(s) for downgrading
	Within-study bias	Reporting bias	Indirectness	Imprecision	Heterogeneity	Incoherence		
VD WP+VD+VE VS P+Ca+CaP	Some concerns	No concerns	No concerns	Some concerns	Some concerns	No concerns	Very low	①④⑤
WP+VD+VE VS WP+Leu	Some concerns	No concerns	Some concerns	Some concerns	Some concerns	No concerns	Very low	①③④⑤
WP+VD+VE VS WP+Leu+LCT	Some concerns	No concerns	No concerns	Major concerns	No concerns	No concerns	Very low	①④
WP+VD+VE VS Leu+VD+ MCT	Some concerns	No concerns	No concerns	Major concerns	No concerns	No concerns	Very low	①④
WP+VD+VE VS Leu+VD+ LCT	Some concerns	No concerns	No concerns	Major concerns	Some concerns	No concerns	Very low	①④⑤
WP+VD+VE VS HMB+ARG+LYS	Some concerns	No concerns	No concerns	Major concerns	No concerns	No concerns	Very low	①④
WP+VD+VE VS Ca+HMB	Some concerns	No concerns	No concerns	Major concerns	No concerns	No concerns	Very low	①④
WP+VD+VE VS P	Some concerns	No concerns	No concerns	Major concerns	No concerns	No concerns	Very low	①④
WP+VD+VE VS P+Ca	Some concerns	No concerns	No concerns	Major concerns	No concerns	No concerns	Very low	①④
ω-3+Leu+PS23 VS WP+VD+Leu	Some concerns	No concerns	No concerns	Major concerns	No concerns	No concerns	Very low	①④

Comparison	Quality assessment						Confidence rating	Reason(s) for downgrading
	Within-study bias	Reporting bias	Indirectness	Imprecision	Heterogeneity	Incoherence		
ω-3+Leu+PS23 VS WP	Some concerns	No concerns	Some concerns	Some concerns	Some concerns	No concerns	Very low	①③④⑤
ω-3+Leu+PS23 VS Leu	Some concerns	No concerns	Some concerns	Some concerns	Some concerns	No concerns	Very low	①③④⑤
ω-3+Leu+PS23 VS VD	Some concerns	No concerns	Some concerns	Some concerns	Some concerns	No concerns	Very low	①③④⑤
ω-3+Leu+PS23 VS P+Ca+CaP	Some concerns	No concerns	No concerns	Major concerns	No concerns	No concerns	Very low	①④
ω-3+Leu+PS23 VS WP+Leu	Some concerns	No concerns	No concerns	Major concerns	No concerns	No concerns	Very low	①④
ω-3+Leu+PS23 VS WP+Leu+LCT	Some concerns	No concerns	No concerns	Major concerns	No concerns	No concerns	Very low	①④
ω-3+Leu+PS23 VS Leu+VD+ MCT	Some concerns	No concerns	No concerns	Major concerns	No concerns	No concerns	Very low	①④
ω-3+Leu+PS23 VS Leu+VD+ LCT	Some concerns	No concerns	No concerns	Major concerns	No concerns	No concerns	Very low	①④
ω-3+Leu+PS23 VS HMB+ARG+LYS	Some concerns	No concerns	No concerns	Major concerns	No concerns	No concerns	Very low	①④
ω-3+Leu+PS23 VS Ca+HMB	Some concerns	No concerns	No concerns	Major concerns	No concerns	No concerns	Very low	①④
ω-3+Leu+PS23 VS	Some concerns	No concerns	No concerns	Some concerns	Some concerns	No concerns	Very low	①④⑤

Comparison	Quality assessment						Confidence rating	Reason(s) for downgrading
	Within-study bias	Reporting bias	Indirectness	Imprecision	Heterogeneity	Incoherence		
P ω-3+Leu+PS23 VS P+Ca	Some concerns	No concerns	No concerns	Some concerns	Some concerns	No concerns	Very low	①④⑤
WP+VD+Leu VS WP	Some concerns	No concerns	Some concerns	Some concerns	Some concerns	No concerns	Very low	①③④⑤
WP+VD+Leu VS Leu	Some concerns	No concerns	No concerns	Major concerns	No concerns	No concerns	Very low	①④
WP+VD+Leu VS VD	Some concerns	No concerns	No concerns	Major concerns	No concerns	No concerns	Very low	①④
WP+VD+Leu VS WP+Leu	No concerns	No concerns	No concerns	Major concerns	No concerns	No concerns	Low	④
WP+VD+Leu VS P+Ca+CaP	Some concerns	No concerns	No concerns	Some concerns	Some concerns	No concerns	Very low	①④⑤
WP+VD+Leu VS Leu+VD+ MCT	Some concerns	No concerns	Some concerns	Some concerns	Some concerns	No concerns	Very low	①③④⑤
WP+VD+Leu VS WP+Leu+LCT	Some concerns	No concerns	No concerns	Major concerns	No concerns	No concerns	Very low	①④
WP+VD+Leu VS HMB+ARG+LYS	Some concerns	No concerns	No concerns	Major concerns	No concerns	No concerns	Very low	①④
WP+VD+Leu VS Leu+VD+ LCT	Some concerns	No concerns	Some concerns	Some concerns	Some concerns	No concerns	Very low	①③④⑤

Comparison	Quality assessment						Confidence rating	Reason(s) for downgrading
	Within-study bias	Reporting bias	Indirectness	Imprecision	Heterogeneity	Incoherence		
WP+VD+Leu VS P	Some concerns	No concerns	No concerns	Major concerns	No concerns	No concerns	Very low	①④
WP+VD+Leu VS Ca+HMB	No concerns	No concerns	No concerns	Major concerns	No concerns	No concerns	Low	④
WP VS Leu	Some concerns	No concerns	Some concerns	Some concerns	Some concerns	No concerns	Very low	①③④⑤
WP+VD+Leu VS P+Ca	Some concerns	No concerns	Some concerns	Some concerns	Some concerns	No concerns	Very low	①③④⑤
WP VS P+Ca+CaP	Some concerns	No concerns	Some concerns	Some concerns	No concerns	No concerns	Very low	①③④
WP VS VD	Some concerns	No concerns	No concerns	Some concerns	Some concerns	No concerns	Very low	①④⑤
WP VS WP+Leu+LCT	Some concerns	No concerns	No concerns	Major concerns	No concerns	No concerns	Very low	①④
WP VS WP+Leu	Some concerns	No concerns	No concerns	Major concerns	No concerns	No concerns	Very low	①④
WP VS Leu+VD+ LCT	Some concerns	No concerns	No concerns	Major concerns	Some concerns	No concerns	Very low	①④⑤
WP VS Leu+VD+ MCT	Some concerns	No concerns	No concerns	Major concerns	No concerns	No concerns	Very low	①④
WP VS	Some concerns	No concerns	No concerns	Major concerns	No concerns	No concerns	Very low	①④

Comparison	Quality assessment						Confidence rating	Reason(s) for downgrading
	Within-study bias	Reporting bias	Indirectness	Imprecision	Heterogeneity	Incoherence		
Ca+HMB WP VS HMB+ARG+LYS	Some concerns	No concerns	No concerns	Major concerns	Some concerns	No concerns	Very low	①④⑤
WP VS P+Ca	Some concerns	No concerns	No concerns	Major concerns	No concerns	No concerns	Very low	①④
WP VS P	Some concerns	No concerns	Some concerns	Some concerns	Some concerns	No concerns	Very low	①③④⑤
Leu VS P+Ca+CaP	Some concerns	No concerns	No concerns	Major concerns	No concerns	No concerns	Very low	①④
Leu VS VD	Some concerns	No concerns	No concerns	Major concerns	No concerns	No concerns	Very low	①④
Leu VS WP+Leu+LCT	Some concerns	No concerns	No concerns	Major concerns	No concerns	No concerns	Very low	①④
Leu VS WP+Leu	Some concerns	No concerns	Some concerns	Some concerns	Some concerns	No concerns	Very low	①③④⑤
Leu VS Leu+VD+ LCT	Some concerns	No concerns	No concerns	Major concerns	No concerns	No concerns	Very low	①④
Leu VS Leu+VD+ MCT	Some concerns	No concerns	No concerns	Major concerns	No concerns	No concerns	Very low	①④
Leu VS Ca+HMB	No concerns	No concerns	No concerns	Major concerns	No concerns	No concerns	Low	④
Leu	Some concerns	No concerns	No concerns	Major concerns	No concerns	No concerns	Very low	①④

Comparison	Quality assessment						Confidence rating	Reason(s) for downgrading
	Within-study bias	Reporting bias	Indirectness	Imprecision	Heterogeneity	Incoherence		
VS HMB+ARG+LYS								
Leu VS P+Ca	Some concerns	No concerns	No concerns	Some concerns	Some concerns	No concerns	Very low	①④⑤
Leu VS P	Some concerns	No concerns	Some concerns	Some concerns	Some concerns	No concerns	Very low	①③④⑤
VD VS WP+Leu	Some concerns	No concerns	No concerns	Major concerns	No concerns	No concerns	Very low	①④
VD VS P+Ca+CaP	Some concerns	No concerns	No concerns	Major concerns	No concerns	No concerns	Very low	①④
VD VS Leu+VD+ MCT	Some concerns	No concerns	No concerns	Some concerns	Some concerns	No concerns	Very low	①④⑤
VD VS Leu+WP+ LCT	Some concerns	No concerns	No concerns	Some concerns	Some concerns	No concerns	Very low	①④⑤
VD VS HMB+ARG+LYS	Some concerns	No concerns	Some concerns	Some concerns	No concerns	No concerns	Very low	①③④
VD VS VD+Leu+LCT	Some concerns	No concerns	No concerns	Major concerns	No concerns	No concerns	Very low	①④
VD VS P	Some concerns	No concerns	No concerns	Major concerns	No concerns	No concerns	Very low	①④
VD VS Ca+HMB	Some concerns	No concerns	Some concerns	Some concerns	Some concerns	No concerns	Very low	①③④⑤

Comparison	Quality assessment						Confidence rating	Reason(s) for downgrading
	Within-study bias	Reporting bias	Indirectness	Imprecision	Heterogeneity	Incoherence		
P+Ca+CaP VS WP+Leu	Some concerns	No concerns	No concerns	Major concerns	No concerns	No concerns	Very low	①④
VD VS P+Ca	Some concerns	No concerns	No concerns	Some concerns	Some concerns	No concerns	Very low	①④⑤
P+Ca+CaP VS VD+Leu+MCT	Some concerns	No concerns	Some concerns	Some concerns	Some concerns	No concerns	Very low	①③④⑤
P+Ca+CaP VS WP+Leu+LCT	Some concerns	No concerns	No concerns	Major concerns	No concerns	No concerns	Very low	①④
P+Ca+CaP VS HMB+ARG+LYS	Some concerns	No concerns	No concerns	Some concerns	Some concerns	No concerns	Very low	①④⑤
P+Ca+CaP VS VD+Leu+LCT	Some concerns	No concerns	Some concerns	Some concerns	Some concerns	No concerns	Very low	①③④⑤
P+Ca+CaP VS P	Some concerns	No concerns	No concerns	Major concerns	No concerns	No concerns	Very low	①④
P+Ca+CaP VS Ca+HMB	Some concerns	No concerns	No concerns	Major concerns	No concerns	No concerns	Very low	①④
WP+Leu VS WP+Leu+LCT	Some concerns	No concerns	Some concerns	Some concerns	No concerns	No concerns	Very low	①③④
P+Ca+CaP VS P+Ca	Some concerns	No concerns	No concerns	Major concerns	No concerns	No concerns	Very low	①④
WP+Leu VS VD+Leu+LCT	Some concerns	No concerns	No concerns	Some concerns	Some concerns	No concerns	Very low	①④⑤

Comparison	Quality assessment						Confidence rating	Reason(s) for downgrading
	Within-study bias	Reporting bias	Indirectness	Imprecision	Heterogeneity	Incoherence		
WP+Leu VS VD+Leu+MCT	Some concerns	No concerns	Some concerns	Some concerns	Some concerns	No concerns	Very low	①③④⑤
WP+Leu VS Ca+HMB	Some concerns	No concerns	No concerns	Major concerns	No concerns	No concerns	Very low	①④
WP+Leu VS HMB+ARG+LYS	Some concerns	No concerns	No concerns	Major concerns	No concerns	No concerns	Very low	①④
WP+Leu VS P+Ca	Some concerns	No concerns	No concerns	Some concerns	Some concerns	No concerns	Very low	①④⑤
WP+Leu VS P	Some concerns	No concerns	No concerns	Major concerns	No concerns	No concerns	Very low	①④
WP+Leu+LCT VS Leu+VD+ LCT	Some concerns	No concerns	No concerns	Some concerns	Some concerns	No concerns	Very low	①④⑤
WP+Leu+LCT VS Leu+VD+ MCT	Some concerns	No concerns	Some concerns	Some concerns	Some concerns	No concerns	Very low	①③④⑤
WP+Leu+LCT VS Ca+HMB	Some concerns	No concerns	No concerns	Major concerns	No concerns	No concerns	Very low	①④
WP+Leu+LCT VS HMB+ARG+LYS	Some concerns	No concerns	No concerns	Major concerns	No concerns	No concerns	Very low	①④
WP+Leu+LCT VS P+Ca	Some concerns	No concerns	No concerns	Major concerns	Some concerns	No concerns	Very low	①④⑤
WP+Leu+LCT VS P	Some concerns	No concerns	No concerns	Major concerns	No concerns	No concerns	Very low	①④
Leu+VD+ MCT VS HMB+ARG+LYS	Some concerns	No concerns	No concerns	Major concerns	No concerns	No concerns	Very low	①④

Comparison	Quality assessment						Confidence rating	Reason(s) for downgrading
	Within-study bias	Reporting bias	Indirectness	Imprecision	Heterogeneity	Incoherence		
Leu+VD+ MCT VS Leu+VD+ LCT	Some concerns	No concerns	No concerns	Major concerns	No concerns	No concerns	Very low	①④
Leu+VD+ MCT VS P	Some concerns	No concerns	No concerns	Major concerns	No concerns	No concerns	Very low	①④
Leu+VD+ MCT VS Ca+HMB	Some concerns	No concerns	No concerns	Major concerns	No concerns	No concerns	Very low	①④
Leu+VD+ LCT VS HMB+ARG+LYS	Some concerns	No concerns	No concerns	Some concerns	Some concerns	No concerns	Very low	①④⑤
Leu+VD+ MCT VS P+Ca	Some concerns	No concerns	No concerns	Some concerns	Some concerns	No concerns	Very low	①④⑤
Leu+VD+ LCT VS P	Some concerns	No concerns	Some concerns	Some concerns	No concerns	No concerns	Very low	①③④
Leu+VD+ LCT VS Ca+HMB	Some concerns	No concerns	No concerns	Major concerns	No concerns	No concerns	Very low	①④
HMB+ARG+LYS VS Ca+HMB	Some concerns	No concerns	No concerns	Major concerns	No concerns	No concerns	Very low	①④
Leu+VD+ LCT VS P+Ca	Some concerns	No concerns	No concerns	Major concerns	Some concerns	No concerns	Very low	①④
HMB+ARG+LYS VS P+Ca	Some concerns	No concerns	No concerns	Major concerns	No concerns	No concerns	Very low	①④

Comparison	Quality assessment						Confidence rating	Reason(s) for downgrading
	Within-study bias	Reporting bias	Indirectness	Imprecision	Heterogeneity	Incoherence		
HMB+ARG+LYS VS P	Some concerns	No concerns	No concerns	Major concerns	No concerns	No concerns	Very low	①④
Ca+HMB VS P+Ca	Some concerns	No concerns	No concerns	Major concerns	No concerns	No concerns	Very low	①④
Ca+HMB VS P	Some concerns	No concerns	No concerns	Major concerns	No concerns	No concerns	Very low	①④
P VS P+Ca	Some concerns	No concerns	No concerns	Major concerns	No concerns	No concerns	Very low	①④

Notes: Downgrading codes: ① within-study bias; ② reporting bias; ③ indirectness; ④ imprecision; ⑤ heterogeneity; ⑥ incoherence.

Supplementary Table S31. Certainty of evidence for each pairwise comparison of nutritional strategies on gait speed in older adults using the CINeMA framework

Comparison	Quality assessment						Confidence rating	Reason(s) for downgrading
	Within-study bias	Reporting bias	Indirectness	Imprecision	Heterogeneity	Incoherence		
Control VS WP+VD+VE	Some concerns	No concerns	No concerns	No concerns	Major concerns	No concerns	Very low	①⑤
Control VS WP+VD+Leu	Some concerns	No concerns	No concerns	No concerns	Some concerns	No concerns	Low	①⑤
Control VS WP	Some concerns	No concerns	No concerns	Major concerns	No concerns	No concerns	Very low	①④
Control VS P+Ca+CaP	Some concerns	No concerns	No concerns	No concerns	Major concerns	No concerns	Very low	①⑤
Control VS WP+Leu	Some concerns	No concerns	No concerns	Some concerns	Some concerns	No concerns	Very low	①④⑤
Control VS WP+Leu+LCT	Some concerns	No concerns	No concerns	Some concerns	Some concerns	No concerns	Very low	①④⑤
Control VS Leu+VD+MCT	Some concerns	No concerns	No concerns	Major concerns	No concerns	No concerns	Very low	①④
Control VS Leu+VD+LCT	Some concerns	No concerns	No concerns	Major concerns	No concerns	No concerns	Very low	①④
Control VS Ca+HMB	No concerns	No concerns	No concerns	Major concerns	No concerns	No concerns	Low	④

Comparison	Quality assessment						Confidence rating	Reason(s) for downgrading
	Within-study bias	Reporting bias	Indirectness	Imprecision	Heterogeneity	Incoherence		
Control VS P	Some concerns	No concerns	No concerns	Major concerns	No concerns	No concerns	Very low	①④
Control VS P+Ca	Some concerns	No concerns	No concerns	Major concerns	No concerns	No concerns	Very low	①④
Control VS WP+CaHMB	Some concerns	No concerns	No concerns	Major concerns	No concerns	No concerns	Very low	①④
WP+VD+VE VS WP+VD+Leu	Some concerns	No concerns	No concerns	Some concerns	Some concerns	No concerns	Very low	①④⑤
WP+VD+VE VS WP	Some concerns	No concerns	Some concerns	Some concerns	Some concerns	No concerns	Very low	①③④⑤
WP+VD+VE VS P+Ca+CaP	Some concerns	No concerns	No concerns	Some concerns	Some concerns	No concerns	Very low	①④⑤
WP+VD+VE VS WP+Leu	Some concerns	No concerns	Some concerns	Some concerns	Some concerns	No concerns	Very low	①③④⑤
WP+VD+VE VS WP+Leu+LCT	Some concerns	No concerns	No concerns	Major concerns	No concerns	No concerns	Very low	①④
WP+VD+VE VS Leu+VD+MCT	Some concerns	No concerns	No concerns	Major concerns	No concerns	No concerns	Very low	①④
WP+VD+VE VS Leu+VD+ LCT	Some concerns	No concerns	No concerns	Major concerns	Some concerns	No concerns	Very low	①④⑤

Comparison	Quality assessment						Confidence rating	Reason(s) for downgrading
	Within-study bias	Reporting bias	Indirectness	Imprecision	Heterogeneity	Incoherence		
WP+VD+VE VS Ca+HMB	Some concerns	No concerns	No concerns	Major concerns	No concerns	No concerns	Very low	①④
WP+VD+VE VS P	Some concerns	No concerns	No concerns	Major concerns	No concerns	No concerns	Very low	①④
WP+VD+VE VS P+Ca	Some concerns	No concerns	No concerns	Major concerns	No concerns	No concerns	Very low	①④
WP+VD+VE VS WP+CaHMB	Some concerns	No concerns	No concerns	Major concerns	No concerns	No concerns	Very low	①④
WP+VD+Leu VS WP	Some concerns	No concerns	No concerns	Major concerns	No concerns	No concerns	Very low	①④
WP+VD+Leu VS P+Ca+CaP	Some concerns	No concerns	No concerns	Major concerns	No concerns	No concerns	Very low	①④
WP+VD+Leu VS WP+Leu	Some concerns	No concerns	No concerns	Major concerns	No concerns	No concerns	Very low	①④
WP+VD+Leu VS WP+Leu+LCT	Some concerns	No concerns	No concerns	Major concerns	No concerns	No concerns	Very low	①④
WP+VD+Leu VS Leu+VD+MCT	Some concerns	No concerns	No concerns	Major concerns	No concerns	No concerns	Very low	①④
WP+VD+Leu VS Leu+VD+ LCT	Some concerns	No concerns	No concerns	Major concerns	No concerns	No concerns	Very low	①④

Comparison	Quality assessment						Confidence rating	Reason(s) for downgrading
	Within-study bias	Reporting bias	Indirectness	Imprecision	Heterogeneity	Incoherence		
WP+VD+Leu VS Ca+HMB	No concerns	No concerns	No concerns	Major concerns	No concerns	No concerns	Low	④
WP+VD+Leu VS P	Some concerns	No concerns	No concerns	Some concerns	Some concerns	No concerns	Very low	①④⑤
WP+VD+Leu VS P+Ca	Some concerns	No concerns	Some concerns	Some concerns	Some concerns	No concerns	Very low	①③④⑤
WP+VD+Leu VS WP+CaHMB	Some concerns	No concerns	No concerns	Major concerns	No concerns	No concerns	Very low	①④
WP VS P+Ca+CaP	Some concerns	No concerns	No concerns	Some concerns	Some concerns	No concerns	Very low	①④⑤
WP VS WP+Leu	Some concerns	No concerns	Some concerns	Some concerns	Some concerns	No concerns	Very low	①③④⑤
WP VS WP+Leu+LCT	Some concerns	No concerns	No concerns	Major concerns	No concerns	No concerns	Very low	①④
WP VS Leu+VD+MCT	Some concerns	No concerns	No concerns	Major concerns	No concerns	No concerns	Very low	①④
WP VS Leu+VD+ LCT	Some concerns	No concerns	Some concerns	Some concerns	Some concerns	No concerns	Very low	①③④⑤
WP VS Ca+HMB	Some concerns	No concerns	No concerns	Some concerns	Some concerns	No concerns	Very low	①④⑤
WP	Some concerns	No concerns	No concerns	Some concerns	Some concerns	No concerns	Very low	①④⑤

Comparison	Quality assessment						Confidence rating	Reason(s) for downgrading
	Within-study bias	Reporting bias	Indirectness	Imprecision	Heterogeneity	Incoherence		
VS<br>P								
WP<br>VS<br>P+Ca	Some concerns	No concerns	Some concerns	Some concerns	Some concerns	No concerns	Very low	①③④⑤
WP<br>VS<br>WP+CaHMB	Some concerns	No concerns	No concerns	Major concerns	No concerns	No concerns	Very low	①④
P+Ca+CaP<br>VS<br>WP+Leu	Some concerns	No concerns	No concerns	Major concerns	No concerns	No concerns	Very low	①④
P+Ca+CaP<br>VS<br>WP+Leu+LCT	Some concerns	No concerns	Some concerns	Some concerns	Some concerns	No concerns	Very low	①③④⑤
P+Ca+CaP<br>VS<br>Leu+VD+MCT	Some concerns	No concerns	Some concerns	Some concerns	Some concerns	No concerns	Very low	①③④⑤
P+Ca+CaP<br>VS<br>Leu+VD+ LCT	Some concerns	No concerns	No concerns	Major concerns	No concerns	No concerns	Very low	①④
P+Ca+CaP<br>VS<br>Ca+HMB	Some concerns	No concerns	No concerns	Some concerns	Some concerns	No concerns	Very low	①④⑤
P+Ca+CaP<br>VS<br>P	Some concerns	No concerns	Some concerns	Some concerns	Some concerns	No concerns	Very low	①③④⑤
P+Ca+CaP<br>VS<br>P+Ca	Some concerns	No concerns	No concerns	Major concerns	No concerns	No concerns	Very low	①④
P+Ca+CaP<br>VS	Some concerns	No concerns	No concerns	Major concerns	No concerns	No concerns	Very low	①④

Comparison	Quality assessment						Confidence rating	Reason(s) for downgrading
	Within-study bias	Reporting bias	Indirectness	Imprecision	Heterogeneity	Incoherence		
WP+CaHMB<br>WP+Leu<br>VS<br>WP+Leu+LCT	Some concerns	No concerns	Some concerns	Some concerns	Some concerns	No concerns	Very low	①③④⑤
WP+Leu<br>VS<br>Leu+VD+MCT	Some concerns	No concerns	No concerns	Major concerns	No concerns	No concerns	Very low	①④
WP+Leu<br>VS<br>Leu+VD+ LCT	Some concerns	No concerns	No concerns	Some concerns	Some concerns	No concerns	Very low	①④⑤
WP+Leu<br>VS<br>Ca+HMB	Some concerns	No concerns	Some concerns	Some concerns	Some concerns	No concerns	Very low	①③④⑤
WP+Leu<br>VS<br>P	Some concerns	No concerns	No concerns	Major concerns	No concerns	No concerns	Very low	①④
WP+Leu<br>VS<br>P+Ca	Some concerns	No concerns	No concerns	Some concerns	Some concerns	No concerns	Very low	①④⑤
WP+Leu+LCT<br>VS<br>VD+Leu+MCT	Some concerns	No concerns	No concerns	Some concerns	Some concerns	No concerns	Very low	①④⑤
WP+Leu+LCT<br>VS<br>VD+Leu+LCT	Some concerns	No concerns	Some concerns	Some concerns	Some concerns	No concerns	Very low	①③④⑤
WP+Leu+LCT<br>VS<br>Ca+HMB	Some concerns	No concerns	No concerns	Major concerns	No concerns	No concerns	Very low	①④
WP+Leu+LCT<br>VS<br>P	Some concerns	No concerns	No concerns	Major concerns	No concerns	No concerns	Very low	①④

Comparison	Quality assessment						Confidence rating	Reason(s) for downgrading
	Within-study bias	Reporting bias	Indirectness	Imprecision	Heterogeneity	Incoherence		
WP+Leu+LCT VS P+Ca	Some concerns	No concerns	No concerns	Major concerns	Some concerns	No concerns	Very low	①④⑤
WP+Leu+LCT VS WP+CaHMB	Some concerns	No concerns	No concerns	Major concerns	No concerns	No concerns	Very low	①④
Leu+VD+ MCT VS VD+Leu+LCT	Some concerns	No concerns	No concerns	Major concerns	No concerns	No concerns	Very low	①④
Leu+VD+ MCT VS Ca+HMB	Some concerns	No concerns	No concerns	Major concerns	No concerns	No concerns	Very low	①④
Leu+VD+ MCT VS P	Some concerns	No concerns	No concerns	Major concerns	No concerns	No concerns	Very low	①④
Leu+VD+ MCT VS P+Ca	Some concerns	No concerns	No concerns	Major concerns	No concerns	No concerns	Very low	①④
Leu+VD+ MCT VS WP+CaHMB	Some concerns	No concerns	Some concerns	Some concerns	Some concerns	No concerns	Very low	①③④⑤
Leu+VD+ LCT VS Ca+HMB	Some concerns	No concerns	No concerns	Some concerns	Some concerns	No concerns	Very low	①④⑤
Leu+VD+ LCT VS P	Some concerns	No concerns	Some concerns	Some concerns	Some concerns	No concerns	Very low	①③④⑤
Leu+VD+ LCT VS P+Ca	Some concerns	No concerns	No concerns	Major concerns	No concerns	No concerns	Very low	①④

Comparison	Quality assessment						Confidence rating	Reason(s) for downgrading
	Within-study bias	Reporting bias	Indirectness	Imprecision	Heterogeneity	Incoherence		
Leu+VD+ LCT VS WP+CaHMB	Some concerns	No concerns	No concerns	Major concerns	No concerns	No concerns	Very low	①④
Ca+HMB VS P	Some concerns	No concerns	No concerns	Major concerns	No concerns	No concerns	Very low	①④
Ca+HMB VS P+Ca	Some concerns	No concerns	No concerns	Major concerns	No concerns	No concerns	Very low	①④
Ca+HMB VS WP+CaHMB	Some concerns	No concerns	No concerns	Major concerns	No concerns	No concerns	Very low	①④
P VS P+Ca	Some concerns	No concerns	No concerns	Major concerns	No concerns	No concerns	Very low	①④
P VS WP+CaHMB	Some concerns	No concerns	No concerns	Major concerns	No concerns	No concerns	Very low	①④
P+Ca VS WP+CaHMB	Some concerns	No concerns	No concerns	Major concerns	No concerns	No concerns	Very low	①④

Notes: Downgrading codes: ① within-study bias; ② reporting bias; ③ indirectness; ④ imprecision; ⑤ heterogeneity; ⑥ incoherence.

Supplementary Table S32. Certainty of evidence for each pairwise comparison of nutritional strategies on five-times sit-to-stand test (5STS) in older adults using the CINeMA framework

**Comparison**	**Quality assessment**						**Confidence rating**	**Reason(s) for downgrading**
	**Within-study bias**	**Reporting bias**	**Indirectness**	**Imprecision**	**Heterogeneity**	**Incoherence**		
Control VS WP+VD+VE	Some concerns	No concerns	No concerns	No concerns	Major concerns	No concerns	Very low	①⑤
Control VS WP+VD+Leu	Some concerns	No concerns	No concerns	No concerns	Some concerns	No concerns	Low	①⑤
Control VS P+Ca+CaP	Some concerns	No concerns	No concerns	No concerns	Major concerns	No concerns	Very low	①⑤
Control VS WP+Leu	Some concerns	No concerns	No concerns	Some concerns	Some concerns	No concerns	Very low	①④⑤
Control VS WP+Leu+LCT	Some concerns	No concerns	No concerns	Some concerns	Some concerns	No concerns	Very low	①④⑤
Control VS P	Some concerns	No concerns	No concerns	Major concerns	No concerns	No concerns	Very low	①④
Control VS P+Ca	Some concerns	No concerns	No concerns	Major concerns	No concerns	No concerns	Very low	①④
WP+VD+VE VS WP+VD+Leu	Some concerns	No concerns	No concerns	Some concerns	Some concerns	No concerns	Very low	①④⑤
WP+VD+VE VS P+Ca+CaP	Some concerns	No concerns	No concerns	Some concerns	Some concerns	No concerns	Very low	①④⑤
WP+VD+VE	Some concerns	No concerns	Some concerns	Some concerns	Some concerns	No concerns	Very low	①③④⑤

Comparison	Quality assessment						Confidence rating	Reason(s) for downgrading
	Within-study bias	Reporting bias	Indirectness	Imprecision	Heterogeneity	Incoherence		
VS<br>WP+Leu								
WP+VD+VE<br>VS<br>WP+Leu+LCT	Some concerns	No concerns	No concerns	Major concerns	No concerns	No concerns	Very low	①④
WP+VD+VE<br>VS<br>P	Some concerns	No concerns	No concerns	Major concerns	No concerns	No concerns	Very low	①④
WP+VD+VE<br>VS<br>P+Ca	Some concerns	No concerns	No concerns	Major concerns	No concerns	No concerns	Very low	①④
WP+VD+Leu<br>VS<br>P+Ca+CaP	Some concerns	No concerns	No concerns	Major concerns	No concerns	No concerns	Very low	①④
WP+VD+Leu<br>VS<br>WP+Leu	Some concerns	No concerns	Some concerns	Some concerns	Some concerns	No concerns	Very low	①③④⑤
WP+VD+Leu<br>VS<br>WP+Leu+LCT	Some concerns	No concerns	Some concerns	Some concerns	Some concerns	No concerns	Very low	①③④⑤
WP+VD+Leu<br>VS<br>P	Some concerns	No concerns	No concerns	Major concerns	No concerns	No concerns	Very low	①④
WP+VD+Leu<br>VS<br>P+Ca	Some concerns	No concerns	Some concerns	Some concerns	Some concerns	No concerns	Very low	①③④⑤
P+Ca+CaP<br>VS<br>WP+Leu	Some concerns	No concerns	No concerns	Some concerns	Some concerns	No concerns	Very low	①④⑤
P+Ca+CaP	Some concerns	No concerns	Some concerns	Some concerns	Some concerns	No concerns	Very low	①③④⑤

Comparison	Quality assessment						Confidence rating	Reason(s) for downgrading
	Within-study bias	Reporting bias	Indirectness	Imprecision	Heterogeneity	Incoherence		
VS<br>WP+Leu+LCT								
P+Ca+CaP<br>VS<br>P	Some concerns	No concerns	No concerns	Major concerns	No concerns	No concerns	Very low	①④
P+Ca+CaP<br>VS<br>P+Ca	Some concerns	No concerns	Some concerns	Some concerns	Some concerns	No concerns	Very low	①③④⑤
WP+Leu<br>VS<br>WP+Leu+LCT	Some concerns	No concerns	No concerns	Major concerns	No concerns	No concerns	Very low	①④
WP+Leu<br>VS<br>P	Some concerns	No concerns	No concerns	Major concerns	No concerns	No concerns	Very low	①④
WP+Leu<br>VS<br>P+Ca	Some concerns	No concerns	Some concerns	Some concerns	Some concerns	No concerns	Very low	①③④⑤
WP+Leu+LCT<br>VS<br>P	Some concerns	No concerns	No concerns	Major concerns	No concerns	No concerns	Very low	①④
WP+Leu+LCT<br>VS<br>P+Ca	Some concerns	No concerns	No concerns	Some concerns	Some concerns	No concerns	Very low	①④⑤
P<br>VS<br>P+Ca	Some concerns	No concerns	Some concerns	Some concerns	Some concerns	No concerns	Very low	①③④⑤

Notes: Downgrading codes: ① within-study bias; ② reporting bias; ③ indirectness; ④ imprecision; ⑤ heterogeneity; ⑥ incoherence.

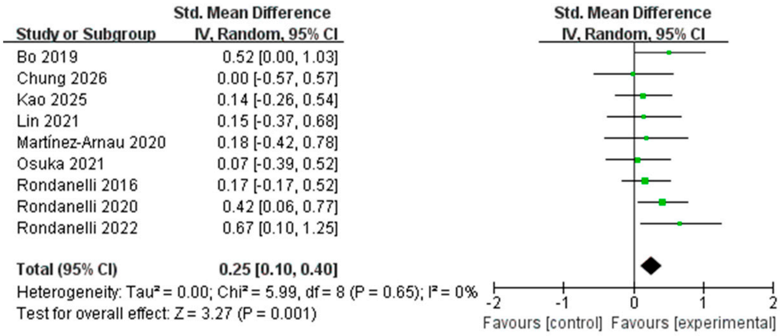

Supplementary Figure S1. Forest plot of pairwise meta-analysis for appendicular skeletal muscle mass index (ASMI)

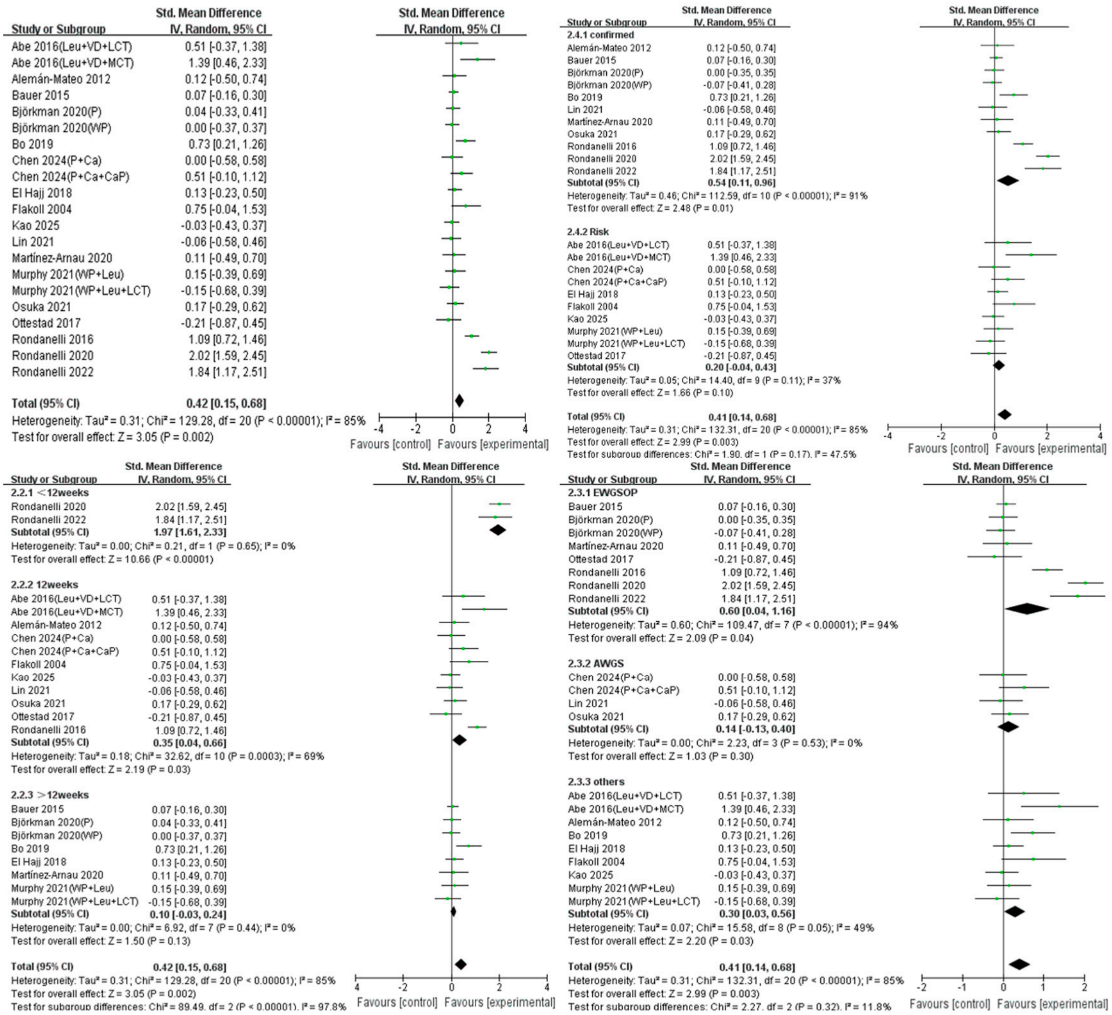

Supplementary Figure S2. Forest plot of pairwise meta-analysis for grip strength, with subgroup analyses

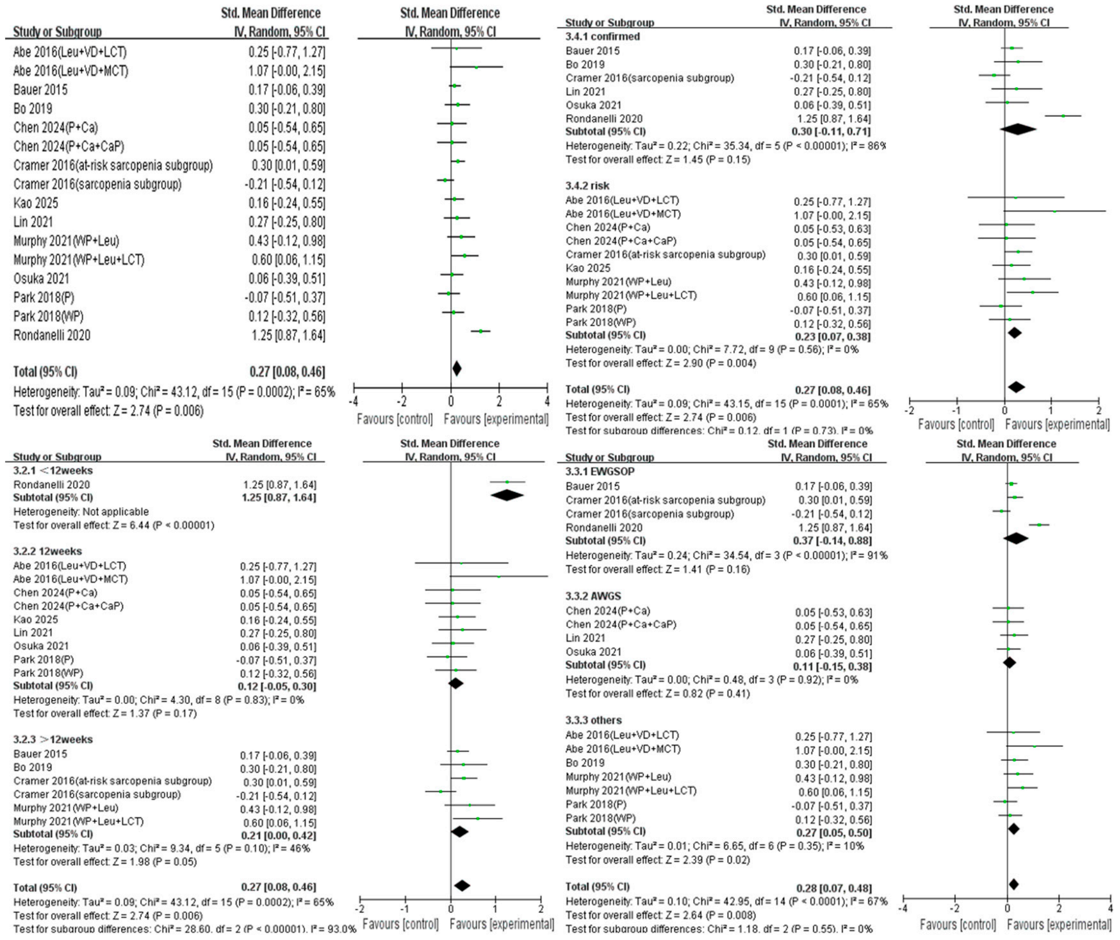

Supplementary Figure S3. Forest plot of pairwise meta-analysis for gait speed, with subgroup analyses

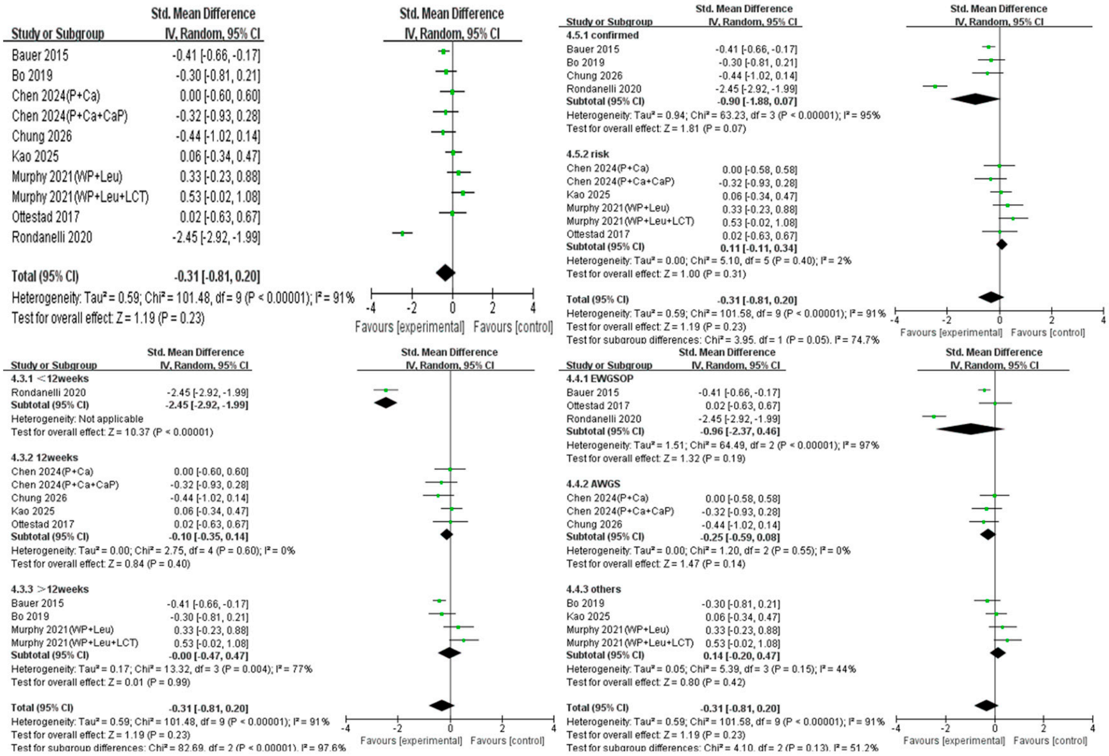

Supplementary Figure S4. Forest plot of pairwise meta-analysis for five-times sit-to-stand test (5STS), with subgroup analyses

Interventions | effect size(95%CI)

Control							
-0.14 (-0.47,0.19)	P						
-0.07 (-0.52,0.39)	0.08 (-0.48,0.64)	Ca+HMB					
-0.14 (-0.80,0.52)	0.00 (-0.57,0.57)	-0.08 (-0.88,0.73)	WP+Leu				
-0.18 (-0.78,0.42)	-0.04 (-0.72,0.65)	-0.11 (-0.86,0.64)	-0.04 (-0.93,0.85)	Leu			
-0.29 (-0.52,-0.06)	-0.15 (-0.50,0.20)	-0.23 (-0.74,0.28)	-0.15 (-0.82,0.52)	-0.11 (-0.76,0.53)	WP+VD+Leu		
-0.67 (-1.25,-0.10)	-0.53 (-1.19,0.13)	-0.61 (-1.34,0.12)	-0.53 (-1.41,0.34)	-0.49 (-1.32,0.34)	-0.38 (-1.00,0.24)	ω-3+Leu+PS23	
-0.52 (-1.03,-0.00)	-0.37 (-0.98,0.24)	-0.45 (-1.14,0.24)	-0.37 (-1.21,0.46)	-0.34 (-1.13,0.45)	-0.22 (-0.79,0.34)	0.16 (-0.61,0.93)	WP+VD+VE

Supplementary Figure S5. League table of network meta-analysis for appendicular skeletal muscle mass index (ASMI)

Interventions | effect size(95%CI)

Control															
0.00 (-1.41,1.41)	P+Ca														
-0.16 (-0.80,0.47)	-0.16 (-1.70,1.38)	P													
-0.17 (-1.53,1.19)	-0.17 (-2.12,1.79)	-0.01 (-1.50,1.49)	Ca+HMB												
-0.75 (-2.25,0.75)	-0.75 (-2.81,1.31)	-0.59 (-2.22,1.04)	-0.58 (-2.61,1.44)	HMB+ARG+LYS											
-0.60 (-2.15,0.95)	-0.60 (-2.69,1.49)	-0.44 (-2.11,1.24)	-0.43 (-2.49,1.63)	0.15 (-2.01,2.31)	Leu+VD+LCT										
-1.39 (-2.96,0.17)	-1.39 (-3.50,0.71)	-1.23 (-2.92,0.46)	-1.23 (-3.30,0.85)	-0.64 (-2.81,1.52)	-0.80 (-2.32,0.73)	Leu+VD+MCT									
0.15 (-1.24,1.54)	0.15 (-1.82,2.12)	0.31 (-1.21,1.84)	0.32 (-1.62,2.26)	0.90 (-1.14,2.94)	0.75 (-1.33,2.83)	1.54 (-0.55,3.63)	WP+Leu+LCT								
-0.15 (-1.54,1.24)	-0.15 (-2.13,1.83)	0.01 (-1.52,1.54)	0.02 (-1.93,1.96)	0.60 (-1.45,2.65)	0.45 (-1.63,2.53)	1.24 (-0.85,3.34)	-0.30 (-1.68,1.08)	WP+Leu							
-0.51 (-1.93,0.90)	-0.51 (-1.92,0.89)	-0.35 (-1.90,1.20)	-0.35 (-2.31,1.62)	0.24 (-1.83,2.30)	0.08 (-2.01,2.18)	0.88 (-1.23,2.99)	-0.66 (-2.65,1.32)	-0.36 (-2.35,1.62)	P+Ca+CaP						
-0.13 (-1.46,1.20)	-0.13 (-2.07,1.80)	0.03 (-1.45,1.50)	0.03 (-1.87,1.94)	0.62 (-1.39,2.62)	0.46 (-1.58,2.51)	1.26 (-0.79,3.32)	-0.28 (-2.21,1.64)	0.02 (-1.91,1.94)	0.38 (-1.56,2.33)	VD					
-0.11 (-1.52,1.31)	-0.11 (-2.10,1.89)	0.06 (-1.49,1.61)	0.06 (-1.90,2.02)	0.64 (-1.42,2.71)	0.49 (-1.61,2.59)	1.29 (-0.82,3.40)	-0.26 (-2.24,1.72)	0.04 (-1.94,2.03)	0.41 (-1.59,2.41)	0.03 (-1.92,1.97)	Leu				
-0.06 (-1.25,1.14)	-0.06 (-1.90,1.79)	0.10 (-1.09,1.30)	0.11 (-1.70,1.92)	0.69 (-1.23,2.61)	0.54 (-1.42,2.49)	1.33 (-0.63,3.30)	-0.21 (-2.04,1.62)	0.09 (-1.74,1.92)	0.45 (-1.40,2.31)	0.07 (-1.72,1.86)	0.05 (-1.80,1.90)	WP			
-0.82 (-1.50,-0.13)	-0.82 (-2.38,0.75)	-0.66 (-1.48,0.17)	-0.65 (-2.17,0.87)	-0.07 (-1.72,1.58)	-0.22 (-1.91,1.47)	0.58 (-1.13,2.29)	-0.97 (-2.51,0.58)	-0.67 (-2.22,0.88)	-0.30 (-1.88,1.27)	-0.68 (-2.18,0.81)	-0.71 (-2.28,0.86)	-0.76 (-2.10,0.58)	WP+VD+Leu		
-1.84 (-3.28,-0.39)	-1.84 (-3.86,0.18)	-1.68 (-3.26,-0.10)	-1.67 (-3.66,0.31)	-1.09 (-3.17,1.00)	-1.24 (-3.36,0.88)	-0.44 (-2.58,1.69)	-1.99 (-3.99,0.02)	-1.69 (-3.69,0.32)	-1.32 (-3.35,0.70)	-1.71 (-3.67,0.26)	-1.73 (-3.75,0.29)	-1.78 (-3.66,0.10)	-1.02 (-2.62,0.58)	ω-3+Leu+PS23	
-0.73 (-2.12,0.65)	-0.73 (-2.71,1.24)	-0.57 (-2.09,0.95)	-0.57 (-2.51,1.37)	0.02 (-2.03,2.06)	-0.14 (-2.21,1.94)	0.66 (-1.43,2.75)	-0.88 (-2.84,1.08)	-0.58 (-2.54,1.38)	-0.22 (-2.20,1.76)	-0.60 (-2.52,1.32)	-0.63 (-2.61,1.35)	-0.67 (-2.50,1.15)	0.08 (-1.46,1.63)	1.11 (-0.90,3.11)	WP+VD+VE

Supplementary Figure S6. League table of network meta-analysis for grip strength

Control													
-0.16 (-0.86,0.53)	WP＋CaHMB												
-0.05 (-0.77,0.67)	0.11 (-0.89,1.11)	P+Ca											
0.08 (-0.41,0.58)	0.25 (-0.45,0.94)	0.14 (-0.74,1.01)	P										
-0.06 (-0.68,0.56)	0.10 (-0.83,1.04)	-0.01 (-0.96,0.95)	-0.14 (-0.94,0.65)	Ca+HMB									
-0.29 (-1.39,0.82)	-0.12 (-1.42,1.18)	-0.23 (-1.55,1.09)	-0.37 (-1.58,0.84)	-0.23 (-1.49,1.04)	Leu+VD+LCT								
-1.28 (-2.43,-0.13)	-1.12 (-2.46,0.23)	-1.23 (-2.59,0.13)	-1.37 (-2.62,-0.11)	-1.22 (-2.53,0.08)	-1.00 (-2.09,0.09)	Leu+VD+MCT							
-0.60 (-1.29,0.09)	-0.44 (-1.42,0.54)	-0.55 (-1.55,0.45)	-0.68 (-1.53,0.16)	-0.54 (-1.47,0.39)	-0.32 (-1.62,0.99)	0.68 (-0.66,2.02)	WP+Leu+LCT						
-0.40 (-1.10,0.29)	-0.24 (-1.22,0.75)	-0.35 (-1.35,0.65)	-0.48 (-1.34,0.37)	-0.34 (-1.27,0.59)	-0.12 (-1.42,1.19)	0.88 (-0.46,2.23)	0.20 (-0.47,0.87)	WP+Leu					
-0.05 (-0.79,0.68)	0.11 (-0.90,1.12)	-0.00 (-0.71,0.71)	-0.14 (-1.02,0.75)	0.01 (-0.96,0.97)	0.23 (-1.10,1.56)	1.23 (-0.14,2.59)	0.55 (-0.46,1.56)	0.35 (-0.66,1.36)	P+Ca+CaP				
-0.11 (-0.69,0.47)	0.05 (-0.32,0.43)	-0.06 (-0.98,0.87)	-0.19 (-0.78,0.39)	-0.05 (-0.90,0.80)	0.17 (-1.07,1.42)	1.17 (-0.12,2.46)	0.49 (-0.41,1.40)	0.29 (-0.62,1.20)	-0.06 (-1.00,0.88)	WP			
-0.17 (-0.60,0.25)	-0.01 (-0.77,0.75)	-0.12 (-0.96,0.72)	-0.26 (-0.77,0.26)	-0.11 (-0.87,0.64)	0.11 (-1.07,1.30)	1.11 (-0.12,2.34)	0.43 (-0.38,1.24)	0.23 (-0.59,1.04)	-0.12 (-0.97,0.73)	-0.06 (-0.73,0.60)	WP+VD+Leu		
-0.30 (-0.96,0.37)	-0.13 (-1.09,0.83)	-0.24 (-1.22,0.74)	-0.38 (-1.21,0.45)	-0.24 (-1.15,0.67)	-0.01 (-1.30,1.28)	0.99 (-0.34,2.32)	0.31 (-0.65,1.26)	0.11 (-0.86,1.07)	-0.24 (-1.23,0.75)	-0.19 (-1.07,0.70)	-0.12 (-0.91,0.67)	WP+VD+VE	

Supplementary Figure S7. League table of network meta-analysis for gait speed

Interventions effect size(95%CI)

Control							
0.00 (-1.79,1.79)	P+Ca						
-0.19 (-1.31,0.94)	-0.19 (-2.30,1.93)	P					
-0.34 (-2.03,1.35)	-0.34 (-2.80,2.12)	-0.15 (-2.02,1.71)	WP+Leu+LCT				
-0.04 (-1.42,1.35)	-0.04 (-2.30,2.23)	0.15 (-1.23,1.54)	0.31 (-1.38,1.99)	WP+Leu			
0.34 (-1.46,2.14)	0.34 (-1.45,2.14)	0.53 (-1.59,2.65)	0.68 (-1.78,3.15)	0.38 (-1.89,2.65)	P+Ca+CaP		
1.41 (0.18,2.63)	1.41 (-0.77,3.58)	1.59 (-0.07,3.26)	1.75 (-0.34,3.83)	1.44 (-0.41,3.29)	1.06 (-1.12,3.24)	WP+VD+Leu	
0.30 (-1.47,2.07)	0.30 (-2.22,2.82)	0.49 (-1.61,2.59)	0.64 (-1.80,3.09)	0.34 (-1.91,2.58)	-0.04 (-2.57,2.48)	-1.10 (-3.26,1.05)	WP+VD+VE

Supplementary Figure S8. League table of network meta-analysis for five-times sit-to-stand test (5STS)

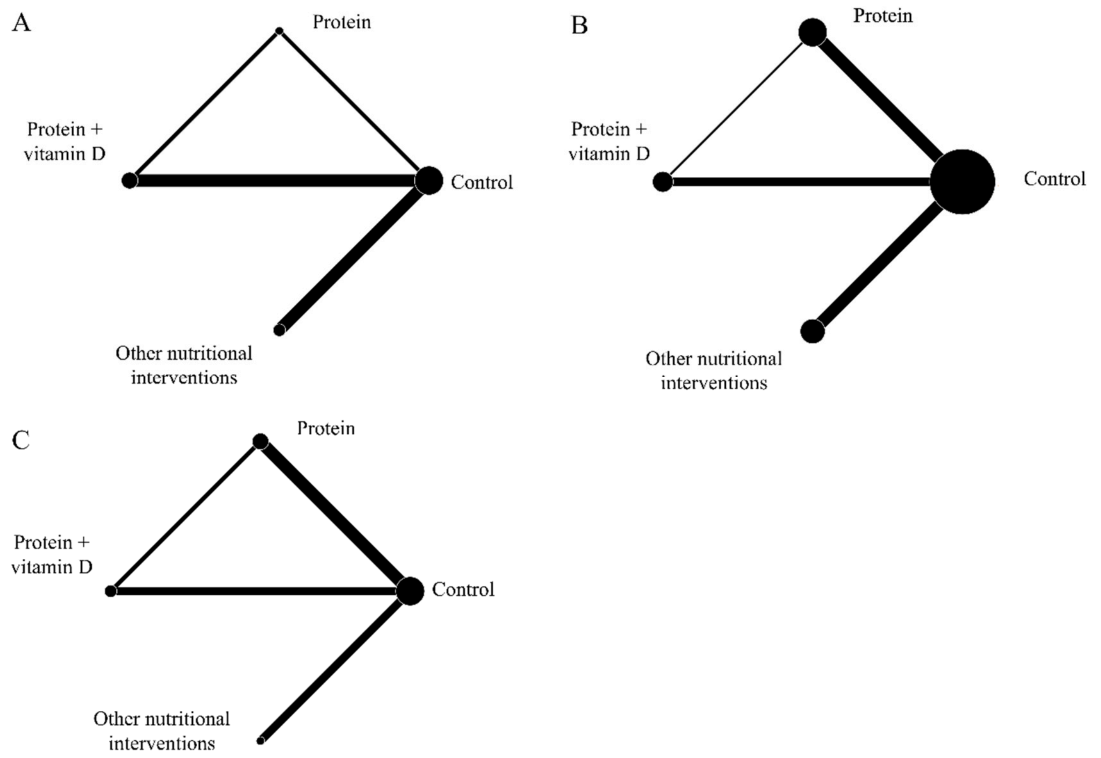

Supplementary Figure S9. Network plots based on the alternative node classification

Notes: Panel A represents appendicular skeletal muscle mass index (ASMI), Panel B represents grip strength, and Panel C represents gait speed. A = Control; B = Protein-based interventions; C = Protein + vitamin D-based interventions; D = Other nutritional interventions.
